# ﻿A revision of *Centrioncus* Speiser (Diptera, Diopsidae, Centrioncinae) with descriptions of new species from Angola, Burundi, and Kenya

**DOI:** 10.3897/zookeys.1144.95619

**Published:** 2023-02-01

**Authors:** Hans R. Feijen, Cobi Feijen

**Affiliations:** 1 Naturalis Biodiversity Center, P. O. Box 9517, 2300 RA Leiden, Netherlands Naturalis Biodiversity Center Leiden Netherlands

**Keywords:** Afromontane forest flies, biogeography, Centrioncinae, diagnoses, key, stalk-eyed flies

## Abstract

A diagnosis is presented for the Centrioncinae, the Afromontane Forest Flies or stalkless Diopsidae, while its taxonomic position within the Diopsidae is discussed. Arguments are presented for an eventual raising of the Centrioncinae to family level. The differential characters for its two genera, *Centrioncus* Speiser and *Teloglabrus* Feijen, are tabulated. The diagnosis for *Centrioncus* is updated and a key to the ten species now recognised (including three new species) is provided. *Centrioncuscrassifemur***sp. nov.** is described from a single female from Angola. This greatly extends the distribution range for the genus. *Centrioncusbururiensis***sp. nov.** is described from Burundi, while *Centrioncuscopelandi***sp. nov.** originates from the Kasigau Massif of Kenya. Diagnoses, descriptive updates, illustrations and notes are presented for all other *Centrioncus*. *Centrioncusaberrans* Feijen, described from Uganda, is now also recorded for western Kenya, Rwanda, and possibly eastern DR Congo. This wide range of *C.aberrans* is unusual for the Centrioncinae species which have allopatric and usually very restricted distribution ranges. Defining characters of *C.aberrans* from the various regions were examined in detail, but only minor differences were found. *Centrioncusdecoronotus* Feijen, described from Kenya, is now recorded for several other places in Kenya. A distribution map is given for the Eastern African *Centrioncus* species. The eastern branch of the Great Rift Valley appears to form a barrier between *C.aberrans* and *C.decoronotus*. The type species of the genus, *C.prodiopsis* Speiser from the Kilimanjaro in Tanzania, was only known from the 1905–1906 type series. After more than 100 years it has now been found again on the Kenya side of Kilimanjaro. Various differential characters of *Centrioncus* and Diopsidae are discussed, while brief discussions on sex ratio and fungal parasites are given. *Centrioncus* are known to occur on low shrubs and herbaceous plants in rain forests. Now, the possibility is indicated that they also might occur higher up in the tree canopies.

## ﻿Introduction

Up till 1983, *Centrioncus* Speiser was known as a monotypic genus, all specimens collected being referred to *Centrioncusprodiopsis* Speiser (Shillito 1950; Hennig 1958, 1965; Smithers 1958; van Bruggen 1961; Steyskal 1970; Griffiths 1972). Although *Centrioncus* was originally placed in the Sepsidae by Speiser (1910), this view was not followed by Frey (1925) who did not include it in his key to the Sepsidae genera. Duda (1925, 1926), in a monograph of the Sepsidae, expressed ambiguous views. Duda (1925) had not seen specimens and found Speiser’s description insufficient. Based on the description, Duda thought that *Centrioncus* could be closer to the Diopsidae than the Sepsidae, while indicating the possibility that it belonged to the Chloropidae. Duda discussed *Centrioncus* in his key to the Sepsidae genera but stated that it did not belong to the Sepsidae: “keinesfalls zu den *Sepsidae* gehörig” [by no means belonging to the Sepsidae]. In 1926, Duda listed *C.prodiopsis* as “sp. dubia” for the Sepsidae.

*Centrioncus* was transferred to the Diopsidae by Shillito (1950), which was accepted by Hennig (1958, 1965). Griffiths (1972) grouped the Diopsidae, including *Centrioncus*, together with the small Neotropical family Syringogastridae in the prefamily Diopsioinea of the Muscoidea. Feijen (1983) proposed the family Centrioncidae, adding five species to *Centrioncus* and 15 species to the new genus *Teloglabrus* Feijen. As vernacular name, the name Afromontane Forest Flies was introduced. The reasons for erecting a new family were based on major morphological differences with the stalk-eyed Diopsidae and on assuming a sister-group relationship between Centrioncidae and the Neotropical Syringogastridae. These two families together were considered a possible sister-group of the Diopsidae. Feijen (1989) updated the diagnosis for the Centrioncidae.

Later studies, based on comparative morphology (McAlpine 1997), on egg morphology (Meier and Hilger 2000) and on morphological and molecular data sets (Meier and Baker 2002; Marshall et al. 2009), disagreed with the separation of Centrioncidae from the Diopsidae. Meier and Baker (2002) assumed a sister group relation between Syringogastridae and Diopsidae s.l. (i.e., including the stalkless genera). Marshall et al. (2009) referred to an unpublished analysis of Diopsoidea family relationships that “confirms Diopsidae (including Centrioncinae) as the sister-group of Syringogastridae”. However, they also stated “The interesting question of diopsid monophyly with respect to *Centrioncus* cannot be tested without adding additional outgroups to this analysis. Our data are equivocal as to whether *Centrioncus* or Diopsidae s.l. is the sister group to *Syringogaster*.” The unpublished analysis mentioned in Marshall et al. (2009) was published by Lonsdale (2020) in a review of the superfamily Diopsoidea, based on a morphological phylogenetic analysis. Lonsdale indicated a monophyletic core group of families: Diopsidae, Megamerinidae and Syringogastridae. The three families were indicated as a robustly supported clade defined by external and genitalic synapomorphies. Diopsidae (including Centrioncinae) were placed as sister-group to the Syringogastridae and these two families together as sister-group of the Megamerinidae.

It appears that the last word on the position of the Diopsidae within Diopsoidea or Acalyptratae has not yet been published. A rather different view on the position of Diopsidae and Syringogastridae was, for instance, given by Wiegmann et al. (2011) in a phylogenomic estimate of fly relationships especially based on molecules from 149 of 157 families. They placed the Diopsidae as the sister group of the Marginidae + Nannodastiidae + Canacidae, while the Syringogastridae were placed remarkably distant and as sister group of the Psilidae. These views were repeated by Wiegmann and Yeates (2017) in a review of phylogeny of Diptera. However, the results of Bayless et al. (2021) were different. Their results were based on analysis of protein encoding sequence data from transcriptomes, including 3145 genes from 70 species, representing all schizophoran superfamilies. They recovered Diopsidae as the sister group to the Syringogastridae, but beyond that, their relationships were unresolved. An overview of publications on the taxonomic position of the Diopsidae is given by Feijen and Feijen (2021). This latter paper also presents a diagnosis for the Diopsidae including the Centrioncinae.

McAlpine (1997) discussed several newly distinguished differential characters for the Centrioncinae, such as the supra-alar carina, pitting along the median ventral suture of the sternopleura, and the sawlines on various tarsal segments. In 2004, De Meyer described as new species *Centrioncusbytebieri* from the Taita hills in Kenya and discussed the stalkless Diopsidae. McAlpine (2011) described and illustrated the antenna of *C.decoronotus*.

The diagnoses for Centrioncinae and *Centrioncus* will now be updated. We will describe *C.crassifemur* sp. nov. from Angola. Furthermore, *C.bururiensis* sp. nov. will be described from Burundi and *C.copelandi* sp. nov. from Kenya. For Eastern Africa, new distribution records will be given for *C.aberrans* Feijen, *C.bytebieri* De Meyer, *C.decoronotus* Feijen and *C.prodiopsis* Speiser, while additional morphological and biometrical data for these species are presented. A distribution map is drawn for the seven *Centrioncus* species now known to occur in Eastern Africa. For the other *Centrioncus*, additional data are given, while photographic illustrations are presented for most species. A key to the ten *Centrioncus* species, now distinguished, will be given. The taxonomic position of the stalkless Diopsidae will be discussed. The relationship between *Centrioncus* and *Teloglabrus* will be considered as will be the intrageneric relationships in *Centrioncus*. Furthermore, various *Centrioncus* characters, sex ratio and fungal parasites will be discussed.

## ﻿Materials and methods

Details on procedures for preparing genitalia slides and procedures for taking measurements are given in Feijen et al. (2018). For information on morphological terminology the reader is referred to the same source. For focus stacking photography of specimens, a Zeiss Stereomicroscope SteREO Discovery.V20 was used. Wings were photographed while mounted in slides. As useful differential character, the ratio width/length of the anterior sclerite of female sternite 7 is now added to the *Centrioncus* descriptions. For this ratio, the width is measured halfway along the length of the sclerite and the length is measured on the meson. The distribution map was built using the online version of SimpleMappr (Shorthouse 2010).

The morphological terminology for the abdomen of Centrioncinae broadly follows the one used by Feijen (1983). However, the sclerite(s) erroneously identified by Feijen (1983) as female sternite 8, were later identified as posterior sclerite(s) of sternite 7, while tergite 9 and sternite 9 in Feijen (1983) were subsequently referred to as tergite 8 and sternite 8 (see Feijen 1987). The aedeagus is now referred to as the phallus. For the femora, the indications outer side and inner side are used. Outer side stands for the side in lateral view (as in Fig. 117) and inner side for the reversed side. For legs, anterior and posterior side are also used (Cumming and Wood 2017), but that can create some confusion. For the fore femora, the outer side represents the posterior side and the inner side the anterior side, while for the mid and hind femora the outer side is the anterior side and the inner side the posterior side. For the bulbous posterior section of the pleura, the term pleurotergal dome is used. Likewise, for the spine on the posterior section of the pleura of stalk-eyed Diopsidae the term pleurotergal spine is used. However, Lonsdale (2020) places these structures more precisely on the katatergite, the ventral section of the pleurotergite.

Feijen (1983) used as terminology for the male genitalia outer and inner surstyli (as telomeres) and interparameral sclerite. Lonsdale (2020) described the inner surstylus as “a lobe of the subepandrial sclerite”. Feijen (1983) assumed the inner surstylus to be “probably of interparameral origin”, so also as part of the subepandrial sclerite (sternite 10). The use of the terms “inner surstylus” or “medial surstylus” was discussed by Norrbom et al. (2019) and Sueyoshi (2005), who also considered it as a “lobe connected basally to the subepandrial sclerite and usually closely associated with the lateral surstylus”. However, the use of the terms outer and inner surstyli for Centrioncinae is now discontinued. The outer surstylus becomes simply the surstylus. The inner surstylus is indicated as the clasper-like lobe of the subepandrial sclerite, for which as short form the term subepandrial clasper will be used. The interparameral sclerite is now indicated as subepandrial sclerite, while the mesal plate-like section will be referred to as subepandrial plate. The epandrial sclerite is not plate-like or even almost absent in stalk-eyed diopsids, but can also be present in the form of a slender rod, referred to as processus longi. The following institutional codens and abbreviations are used:

**BMSA**National Museum Bloemfontein, Bloemfontein, South Africa;

**CNC**Canadian National Collection of Insects, Ottawa, Ontario, Canada;

**FBUB**Biological Collection, Universität Bielefeld, Bielefeld, Germany;

**ICIPE** International Centre of Insect Physiology and Ecology, Nairobi, Kenya;

**MRAC**Musée Royal de l’Afrique Centrale, Tervuren, Belgium;

**NHMUK**Natural History Museum, London, United Kingdom;

**NHRS**Naturhistoriska Riksmuseet, Stockholm, Sweden;

**NMKE**National Museum of Kenya, Nairobi, Kenya;

**NMSA**KwaZulu-Natal Museum, Pietermaritzburg, South Africa;

**RMNH**Naturalis Biodiversity Center (formerly Rijksmuseum van Natuurlijke Historie), Leiden, The Netherlands;

**ZMHB**Museum für Naturkunde der Humboldt-Universität, Berlin, Germany;

**ZMUM**Zoological Museum, Moscow State University, Moscow, Russia.

**ap. seta** (scutellar) apical seta

**F1** fore femur

**F3** hind femur

**FOS** fronto-orbital seta

**l.** length

**l/w** (ratio) length/width

**OVS** outer vertical seta

**sc.** scutellum

**sc. sp.** scutellar spine

**SE** standard error

**Sp.** spiracle

**St.** sternite

**T.** tergite

**w.** width

**w/l** (ratio) width/length

## ﻿Taxonomy

### ﻿Family Diopsidae

Taxon classificationAnimaliaDipteraDiopsidae

Billberg, 1820

79322DE5-1208-521C-96B2-C06E93B24056

Diopsidae : Billberg 1820: 115 (as Natio Diopsides).

#### Type genus.

*Diopsis* Linnaeus, 1775: 5.

### ﻿Subfamily Centrioncinae

Taxon classificationAnimaliaDipteraDiopsidae

Hennig, 1965

59930BB6-F858-501A-BFC1-693ADF28C4C1

Centrioncinae : Hennig, 1965: 62; Shillito 1971: 288; Steyskal 1972: 1; Feijen 1983: 67 (as Centrioncidae); Feijen 1989: 115 [as Centrioncidae]; McAlpine 1997: 170; Hilger 2000: 335; Meier and Hilger 2000: 1, 6; Baker et al. 2001: 89; Meier and Baker 2002: 332; Nartshuk 2003: 246 [as Centrioncidae]; De Meyer 2004: 25; Kotrba and Balke 2006: 843; Kotrba et al. 2010: 299; McAlpine 2011: 150; Feijen and Feijen 2021: 1523.

#### Type genus.

*Centrioncus* Speiser, 1910: 190.

#### Diagnosis of Centrioncinae.

**Figures 1–4.**
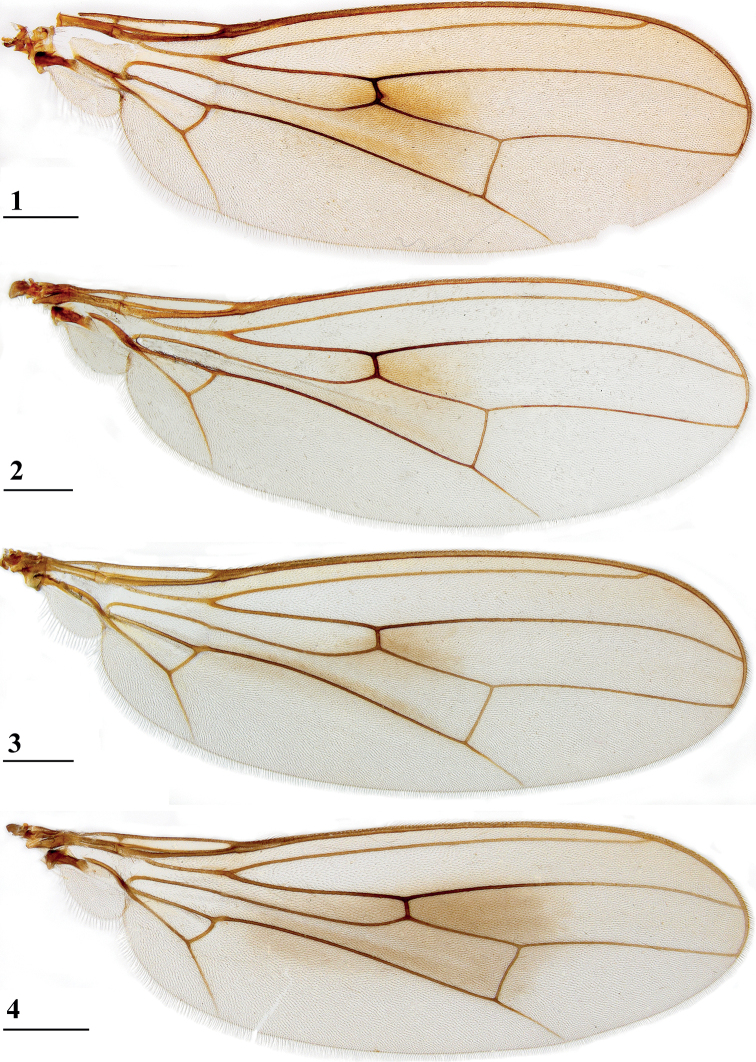
*Centrioncus*, dorsal view of wings **1** ♂, *C.aberrans*, Timboroa Forest **2** ♀, paratype, *C.bururiensis* sp. nov., Bururi Nat. Forest **3** ♂ *C.bytebieri*, Ngangao Forest **4** ♀, paratype, *C.copelandi* sp. nov., Kasigau Mtn. Scale bars: 0.5 mm.

**Figures 5–8.**
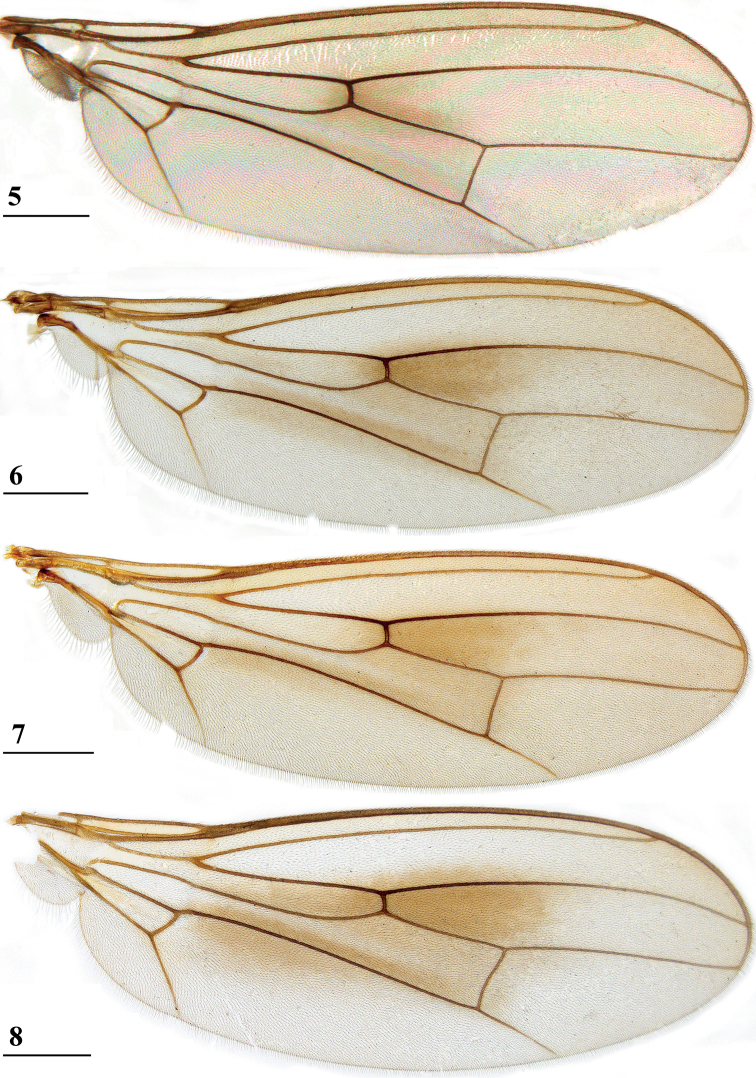
*Centrioncus*, dorsal view of wings **5** ♀, holotype, *C.crassifemur* sp. nov., N’dalatando **6** ♂, *C.decoronotus* Njuki-Ini Forest **7** ♂, *C.jacobae* Mount Soche **8** ♀, *C.prodiopsis*, Oloitokitok. Scale bars: 0.5 mm.

Updated version of the diagnosis by Feijen (1989) for Centrioncidae. Small (4–7 mm), slender flies; head rounded (Fig. 11); eyes not stalked; pedicellus without cleft and in lateral view strongly asymmetrical; funiculus (=first flagellomere) ventrally extended within sagittal plane ~ 1.7× the width of pedicellus, funiculus with a large basal hollow on dorsal half, into which the strongly asymmetrical pedicellar conus is inserted; tripartite pubescent arista; one pair of outer vertical setae and one pair of fronto-orbital setae; lanceolate basiliform prosternum (Fig. 129); supra-alar ridge (carina) present; no pleurotergal spines, three pairs of scutal setae, one pair of scutellar spines with apical setae (Fig. 117), apical setae varying from 20% shorter to 25% longer than spines; costa unbroken, alula present (Figs 1–8), veins CuA+CuP and M4 reaching margin, impression of crossvein bm-cu visible, wings transparent with or without vague spots; incrassate fore femora with double series of ventral tubercles and spinous setae; slender hind femora with distal set of small tubercles; no apical spurs on mid and hind femora; syntergite consisting of tergites 1 and 2; ♀ segment 7 sclerotised basally in complete ring, ♀ sternite 7 transversely divided, ♀ tergite 8 and sternite 8 each consisting of two sclerites; ♂ tergite 6 half as long as tergite 5, main part of ♂ sternite 7 located on left side, very large inverted ♂ sternite 8 (Fig. 136) on both sides fused to sternite 7 which forms a complete ventral band of sclerotisation (Feijen and Feijen 2021: fig. 38); abdominal spiracles 1–6 in membrane, ♀ 7^th^ spiracles in tergite or not, ♂ left 7^th^ spiracle in laterally located sternite 7 and ♂ right 7^th^ spiracle in ventral band of sclerotisation; three (2 + 1) spermathecae; elongate, striated eggs; articulated three-lobed surstylus with tubercles and often spinous setae; subepandrial sclerite with clasper-like ventral lobes and large mesal plate; membranous ♂ cercus; small phallapodeme with posterior two-thirds fused to hypandrium, hypandrial clasper usually present; postgonites and epiphallus present, phallus a short solid structure with a complex distal section; ejaculatory apodeme + sac present, seminal ducts without distinct reticulated transverse structures.

### ﻿Genus Centrioncus

Taxon classificationAnimaliaDipteraDiopsidae

Speiser, 1910

1B759F9F-D11E-53F5-9C6A-02E5D6785342

Centrioncus Speiser, 1910: 190; Frey 1925: 69; Duda 1925: 22, 24; Duda 1926: 110; Shillito 1950: 109; Smithers 1958: 25; van Bruggen 1961: 422; Steyskal 1970: 325; Griffiths 1972: 167; Feijen 1983: 4; Feijen 1989: 115; McAlpine 1997: 175, figs 21, 22, 35, 42; De Meyer 2004: 25; McAlpine 2011: 150, figs 122, 124; Feijen and Feijen 2021: 1535.

#### Type species.

*Centrioncusprodiopsis* Speiser, 1910: 191, by monotypy.

#### Remark.

Various papers from between 1925 and 1983 can refer to the second Centrioncinae genus *Teloglabrus* or a mixture of the two genera (see Feijen 1983).

#### Diagnosis of *Centrioncus*.

Updated version of the diagnosis by Feijen (1983). Centrioncinae with dark maxillary palps; dark section of funiculus limited to around base of arista; central region behind ocellar tubercle usually dark; wing with cell c partly or wholly glabrous, distal section of vein M4 gradually thinner, central wing spot present (Figs 1–8); on average 7–10 spinous setae per fore femur; basal ring of ♀ segment 7 with or without sutures; ♀ tergite 7 well sclerotised and with lateral edges curved under, ♀ sternite 7 split into a broad rectangular anterior plate and a curved or rectangular posterior plate (Figs 14, 104, 133, 148); ♀ 7^th^ spiracles in tergite or membrane; subanal plate large; epandrium broad and rounded with ratio width/length 1.4–1.5; inner arm of surstylus quite detached from common base of outer and median arms; surstylus in dorsal to dorsolateral position (due to inversion appearing ventrally); outer and median arms with patches of microtrichia on outer side; connection between surstylus and subepandrial clasper short; subepandrial clasper glabrous, without ridges, with 1–3 long setae and 2–8 short setae; inner posterior corner of epandrium without mesad extension for articulation with subepandrial clasper; articulation between subepandrial clasper and cercus via 1–2 small mesad sclerites, the sclerites of both sides linked via a membranous connection; ♂ cercus with distally a broad lateral extension or cercus slender; subepandrial sclerite anteriorly with large lateral extensions, w/l ratio 2.0–2.5; epandrial fold broad and short or epandrial sclerite present; hypandrial clasper with three terminal setae or absent; epiphallus well denticulated, lateral sides of phallophore distally acuminate, phallus rather broad, distal phallic sclerites large and U-shaped; ejaculatory apodeme + sac very large (9.2–16.3% of body length), proximal section of ejaculatory duct at right angles to ejaculatory apodeme.

**Table 1.** Differential character states for the genera *Centrioncus* and *Teloglabrus* (major differences indicated with an asterisk – *).

	Character	*Centrioncus*	*Teloglabrus*
	maxillary palpi	usually dark	usually yellowish
	dark apical section of funiculus	limited to around base of arista	≥ dorsal quarter of lateral side
*	central wing spot	present	usually absent
*	anterior sclerite of ♀ sternite 7	1 large rectangular to trapezoidal plate	2 narrow elongate plates anteriorly connected on meson
*	posterior sclerite of ♀ sternite 7	1 curved (U-shaped) or rectangular plate	2 small sclerites or absent
*	♀ spiracle 7	in tergum (9 of 10 species, 1 species in membrane)	all species in membrane
	♀ basal ring of segment 7	with or without suture	without suture
*	epandrium	broad and rounded	rather narrow and bell-shaped
	epandrium: ratio width/length	1.3–1.5	1.0–1.1, in few species 1.2–1.3
	surstylus	dorsal to dorsolateral position (due to inversion “ventrally”)	lateral position
*	outer and median arms of surstylus	pruinose areas on outer side	no microtrichia
	connection between surstylus and subepandrial clasper	short	usually long
*	subepandrial clasper	glabrous, no ridges, with 1–3 long setulae and 2–8 short setulae	with microtrichia, usually with ridges, no long setulae and 12–35 short setulae
	subepandrial plate	Anteriorly with large lateral extensions, w/l ratio: 2.0–2.5	small or no lateral extensions, w/l ratio: 1.0–1.8
*	ejaculatory apodeme + sac	very large (9.3–16.5% of body length)	normal-sized (5.6–8.6% of body length) – 14 of 16 species are between 5.6–7.3%
*	proximal section of ejaculatory duct	perpendicular to apodeme	in line with apodeme
	hypandrial clasper	with 3 terminal setulae or absent	with 1 or 2 terminal setulae

#### Remark.

*Centrioncus* species are known from Angola, West Africa, and eastern Africa north of the Zambesi (north of 16°S latitude).

### ﻿Key to the genera of Centrioncinae and species of *Centrioncus*

Although several *Centrioncus* and *Teloglabrus* have striking colour patterns of black and reddish brown on the thorax, this pattern is often not visible in preserved specimens, while it can sometimes also not be discerned in photographs of live flies. Wing patterns of central and apical wing spots are subtle and often cannot be distinguished in mounted flies or photographs of live flies. Mounting a detached wing in a slide is the best way to view the pattern. For confirmation of identifications, study of female and/or male genitalia is required. A problem for the *Centrioncus* key is that only females are known for three species (*C.angusticercus* Feijen, *C.decellei* Feijen, and *C.crassifemur* sp. nov.), so corroborating male characters cannot be verified. The unique holotypes for *C.angusticercus* and *C.decellei* were not available for study, so newly used characters, such as ratios involving the scutellar spine and the state of sternites 4, 5, and 6, could not be verified. Given the usually restricted distribution ranges of the allopatric species, the location of origin forms an important indication of the species involved. Only *C.aberrans* and, to some extent *C.decoronotus*, are known to have a broader distribution range.

The first couplet of the key separates the genera *Centrioncus* and *Teloglabrus*. This issue is also treated in Table 1 where the major differences between these genera are listed. In addition, the differences are considered in the discussion section under “Distinctive characters for *Centrioncus* and *Teloglabrus*”.

**Table:** 

1	Anterior sclerite of ♀ sternite 7 single, rectangular, posterior sclerite of ♀ sternite 7 single, strongly curved or rectangular (Figs 14, 104, 133, 148); epandrium broad and rounded with w/l ratio: 1.3–1.5; ejaculatory apodeme + sac very large (9.3–16.3% of body length); ejaculatory duct near sac perpendicular to apodeme; surstylus pruinose on outer side; maxillary palpi usually dark; central wing spot (Figs 1–8). Distribution: Angola, West Africa and eastern Africa north of Zambezi (north of 16°S latitude)	***Centrioncus*** (**2)**
–	Anterior section of ♀ sternite 7 divided into two sclerites anteriorly narrowly connected on meson, posterior section of ♀ sternite 7 divided into two small sclerites or absent; epandrium rather narrow and bell-shaped, usually with w/l ratio: 1.0–1.1 (1.2–1.3 in some species); ejaculatory apodeme + sac 5.6–8.4% of body length; ejaculatory duct near sac more or less aligned with apodeme; surstylus glabrous on outer side; maxillary palpi usually yellow; usually without central wing spot. Distribution: south of Zambezi (south of 19°S latitude)	***Teloglabrus***
2	Fore femur on distal third of both sides dark brown (outer side with distal spot in *C.angusticercus*); w/l ratio of anterior sclerite of ♀ sternite 7: ≥ 4.4; posterior sclerite of ♀ sternite 7 more or less rectangular (Figs 14, 32, 104), not curved U-shaped	**3**
–	Fore femur only on inner side with dark brown distal stripe; w/l ratio of anterior sclerite of ♀ sternite 7: < 3.0 (3.9 in *C.decoronotus*); posterior sclerite of ♀ sternite 7 broad inverted U-shaped (Figs 133, 148)	**5**
3	Fore femur with distal third on inner side dark brown and with small dark spot apically on outer side; small central wing spot around crossvein r-m not reaching vein R4+5; w/l ratio of anterior sclerite of ♀ sternite 7: ~ 4.4; posterior sclerite of ♀ sternite 7 well sclerotised, trapezoidal and short with weakly sclerotised posterolateral extensions (Fig. 32); ♀ cercus with l/w ratio: 5.4. Distribution: South Sudan	***Centrioncusangusticercus***
–	Fore femur with distal third on both sides dark brown; small central wing spot around crossvein r-m reaching vein R4+5 in cells br and r4+5; w/l ratio of anterior sclerite of ♀ sternite 7: > 5; posterior sclerite of ♀ sternite 7 weakly sclerotised, trapezoidal to rectangular with more sclerotised anterolateral areas (Figs 14, 102, 104); ♀ cercus with l/w ratio: 4.3 or 5.1	**4**
4	Collar glossy; incrassate fore femur (mean l/w ratio: 2.75, range 2.68–2.84); fore femur with ~ 35 tubercles (range 29–41); vein CuA+CuP curving downward to wing margin (Fig. 1); apical seta 10% longer than scutellar spine; anterior sclerite of ♀ sternite 7 with w/l ratio: ~ 5.4 (Fig. 14); posterior sclerite of ♀ sternite 7 with two large more sclerotised sections touching on meson (Figs 13, 14); ♀ sternite 8 consisting of two oblong sclerites separated on meson; spermathecae spherical with small apical dimple (Fig. 15). Distribution: Uganda, Rwanda, Kenya (west of Great Rift Valley)	***Centrioncusaberrans***
–	Collar mainly pruinose; strongly incrassate fore femur (l/w ratio: 2.36); fore femur with ~ 42 tubercles (range 41–42); vein CuA+CuP extending to wing margin in almost straight line (Fig. 5); apical seta 20% shorter than scutellar spine; anterior sclerite of ♀ sternite 7 with w/l ratio: ~ 8.6 (Fig. 104); membranous posterior sclerite of ♀ sternite 7 with two tiny more sclerotised sections in anterolateral corners; ♀ sternite 8 consisting of two sclerites broadening posteriorly and posteriorly linked on meson; spermathecae flattened with large apical dimple and central pinnacle (Figs 106, 107). Distribution: Angola	***Centrioncuscrassifemur* sp. nov.**
5	Frons flat and wholly pruinose; fore femur on distal half of inner side dark brown; posterior sclerite of ♀ sternite 7 U-shaped, with short arms and 3-lobed apices (Fig. 111); subanal plate comprising equilateral triangle with slightly convex sides and pointed apex. Distribution: Ivory Coast	***Centrioncusdecellei***
–	Frons mesally depressed, usually with glossy spots laterally of ocellar tubercle (only *C.bururiensis* sp. nov. without glossy spots); fore femur with dark brown distal stripe on inner side (only *C.bururiensis* sp. nov. without stripe but femur dark brown on distal third of inner side); posterior sclerite of ♀ sternite 7 U-shaped with longer arms and tapering, rounded or broadening apices (Figs 43, 62, 81, 119, 133, 148); subanal plate usually pentagonal (only *C.bytebieri* with plate comprising isosceles triangle, with longer base and rounded apex)	**6**
6	Frons wholly pruinose; fore femur usually with 38 or 39 tubercles (range 35–43); fore femur on distal third of inner side dark brown; female 7^th^ spiracle in membrane; U-shaped female posterior sclerite of sternite 7 with apices tapering and posteriorly directed (Fig. 43); outer arm of surstylus triangular with base much wider than apex (Fig. 51); median arm of surstylus very broad and parallel-sided (Fig. 51), with 11 spinous setae; male cercus without distal lateral extension (Fig. 54). Distribution: Burundi	***Centrioncusbururiensis* sp. nov.**
–	Frons with pair of glossy spots lateral to ocellar tubercle; fore femur usually with 31 to 35 tubercles (range 26–40); fore femur with dark brown stripe on distal third to distal three-fifths of inner side; female 7^th^ spiracle in tergite; apices of U-shaped posterior sclerite of sternite 7 not tapering, or if slightly tapering laterally directed (Figs 62, 81, 119, 133, 148); outer arm of outer surstylus not triangular and constricted at base (Figs 69, 88); median arm of surstylus slender or club-shaped (Figs 69, 88, 138), with 0–5 spinous setae; male cercus with distal lateral extension (Figs 71, 89, 123)	**7**
7	Fore femur usually with 31 or 32 tubercles (range 28–33); central wing spot very large, extending into apical third of cell br; wing spot in cell r4+5 distally extending well past crossvein dm-m (Figs 4, 8); tergites uniformly blackish brown; sternite 5 anteriorly strongly invaginated mesally (Figs 80, 146); female cercus with l/w ratio: 4.0–4.4; outer arm of surstylus apically with 16 or 17 tubercles; median arm of surstylus slightly shorter than outer arm, with some long, normal apical setae only (Fig. 150); subepandrial clasper elongate	**8**
–	Fore femur usually with 32 to 35 tubercles (range 26–40); central wing spot small to large, wing spot only in apex of cell br and in cell r4+5 extending to proximal of, or to crossvein dm-m (Figs 3, 6, 7); tergites (especially 2 and 3) with pale posterolateral spots (Figs 60, 135); sternite 5 anteriorly straight (Fig. 132); female cercus with l/w ratio: 2.3–3.6; outer arm of surstylus apically with 4–9 tubercles; median arm of surstylus longer than outer arm, with 3–6 spinous apical setae (Figs 69, 122, 138); subepandrial clasper trapezoidal to triangular	**9**
8	Pleura blackish brown; fore femur with l/w ratio: 2.65; anterior edge of sternite 4 straight; posterior sclerite of sternite 7 truncated U-shaped, with straight basal and lateral edges (Fig. 82); spermathecae with small dimple; distinct constriction in spermathecal ducts near spermathecae (Fig. 85); outer arm of surstylus with concave sides, apically 3× as wide as at base; inner arm of surstylus longer than median arm; subepandrial clasper apically strongly convex, with apical corners angular (Fig. 90). Distribution: Kenya, Kasigau Mtn	***Centrioncuscopelandi* sp. nov.**
–	Pleura blackish brown with brown markings; fore femur with l/w ratio: 2.92; anterior edge of sternite 4 strongly invaginated mesally (Fig. 146); posterior sclerite of sternite 7 smoothly rounded U-shaped (Fig. 148); spermathecae cup-shaped due to very large dimple; spermathecal ducts without constriction near spermathecae; outer arm of surstylus with straight sides, apically 2× as wide as at base; inner arm of surstylus half-length of median arm; subepandrial clasper apically hardly convex, with apical corners rounded (Fig. 151). Distribution: Kenya, Tanzania (around Kilimanjaro)	***Centrioncusprodiopsis***
9	Scutum blackish brown, humeral calli blackish brown; fore femora with average l/w ratio: 3.30 (range 3.17–3.43); central wing spot small (Fig. 3), wing spot in base of cell r4+5 not extending to crossvein dm-m; sternite 4 trapezoidal, sternites 4 and 5 without heavily sclerotised areas; posterior sclerite of female sternite 7 with large posterolateral extensions (Fig. 62); outer arm of surstylus gradually tapering towards base (Fig. 69); subepandrial clasper basally somewhat constricted (Fig. 70). Distribution: Kenya, Dabida Massif of Taita Hills	***Centrioncusbytebieri***
–	Scutum (in prime specimens) with configuration of blackish brown and brown (Figs 112, 127), humeral calli brown; fore femora with average l/w ratio: 2.78–2.89 (range 2.64–3.05); central wing spot large (Figs 6, 7), in base of cell r4+5 extending to crossvein dm-m, sternite 4 rectangular; sternites 4 and 5 with small, heavily sclerotised areas (Figs 118, 132); posterior sclerite of female sternite 7 without posterolateral extensions (Fig. 133); outer arm of surstylus rounded (Figs 122, 138); subepandrial clasper basally strongly constricted (Figs 124, 138)	**10**
10	Pleura blackish brown with chestnut brown anterodorsal anepisternum, greater ampulla and posterior anepimeron (Fig. 113); scutellar spine/scutellum ratio: 0.90; apical seta/scutellar spine ratio: 0.91; tergite 2 with small pale posterolateral corners; small, heavily sclerotised areas on sternites 4–6; anterior sclerite of female sternite 7 with w/l ratio: 3.9; apex of subanal plate with acuminate extension; common base of outer and median arms of surstylus long and slender (Fig. 122); outer arm of surstylus with 4–6 tubercles; median arm slender rod. Distribution: Kenya, east of Great Rift Valley	***Centrioncusdecoronotus***
–	Pleura chestnut-brown with small blackish area around anterior spiracle and largely blackish posterior third (Fig. 127); scutellar spine/scutellum ratio: 0.78; apical seta/scutellar spine ratio: 1.11; tergite 2 with large rectangular pale posterolateral spots (Fig. 135); small, heavily sclerotised areas only on sternites 4 and 5; anterior sclerite of female sternite 7 with w/l ratio: 2.6; apex of subanal plate angular, without acuminate extension; common base of outer and median arms of surstylus short and broad (Fig. 138); outer arm with 6–9 tubercles; median arm club-shaped. Distribution: southern Malawi	***Centrioncusjacobae***

### ﻿ Centrioncus aberrans

Taxon classificationAnimaliaDipteraDiopsidae

Feijen

4453C226-2EEA-5716-8230-373D68A5F8C6

Figs 1 , 9–12 , 13–15 , 16–20 , 21–24 , 25–28 , 29 , 30 , Tables 2 , 6 , 8 , 9

Centrioncus aberrans Feijen, 1983: 84.
Centrioncus sp. Feijen 1983: 87 (this specimen from Rwanda, Lac Gando, was only cursorily inspected, but could now be studied in detail).

#### Type material.

Uganda: ***holotype***, ♂, Kilembe, Ruwenzori Range, [0°11'51"N, 30°0'47"E], 1500 m, F.W. Edwards (NHMUK). ***Paratypes***: 1 ♀, 1 ♂, same data as holotype (NHMUK).

#### Material studied.

Kenya: 8 ♀, 12 ♂, Mount Elgon, nr Elephant Cave (Kitum cave), [1°1'56.84"N, 34°45'28.94"E, 2350 m], 22.iii.1988, H.R. Feijen (RMNH); 1 ♂, Saiwa Swamp, [1°5'43.60"N, 35°6'59.45"E, 1870 m], 23.iii.1988, H.R. Feijen (RMNH); 3 ♂, Rift Valley Province, Timboroa Forest (compt. 9), malaise traps, indigenous Afromontane forest, 00°04.092'S, 35°30.909'E, 2628 m, 14–16.iv.2011, A.H. & M.K. Kirk-Spriggs (BMSA); 1 ♀, Rift Valley Prov., Lake Nakuru N.P., malaise trap, *Acaciaxanthophloea* habitat, 0.35130°S, 36.05795°E, 1796 m, 3-17.iii.2006, R. Copeland (ICIPE); Rwanda: 1 ♂, N. Kivu, Lac Gando, 1°36'S, 29°24'E, 2400 m, 25.xii.1925, H. Schouteden (MRAC); Rwanda & DR Congo [as Zaire]: 2 ♀, x.1993, Thomas Wagner, canopy fogging on specific trees (FBUB) [According to Dr Wagner (pers. comm.), there are two options for the collection site: Rwanda, Cyamudongo, Nyakabuye, 2°34'S, 28°59'E, 1750 m, montane rain forest or DR Congo Irangi, Kivu-Sud, 1°54'S, 28°27'E, 950 m, rain forest. The distance between the two sites is 95 km in a straight line.]. In total 12 ♀ and 19 ♂ were studied.

#### Diagnosis.

*Centrioncusaberrans* can be recognised by its mesally depressed, pruinose frons with two small glossy spots; glossy, blackish brown collar; pruinose, blackish brown scutum; pruinose, brown scutellum with two dark spots; brown scutellar spines; blackish brown pleura, but propleuron, anterior half and posteroventral corner of anepisternum, and dorsal “knob” of anepimeron chestnut brown (Fig. 12); scutellar spine/scutellum ratio: 0.95; apical seta/scutellar spine ratio: 1.13; brown fore femur on distal quarter dark brown on both sides, strongly incrassate (l/w ratio: 2.75) with ~ 35.5 tubercles; medium-sized central wing spot largely in basal quarter of cell r4+5, extending into cells br and bm+dm (Fig. 1); tergites dark brown, thinly pruinose; sternite 4 rectangular (Fig. 13), sternite 5 trapezoidal; sternite 6 a short, broad, trapezoidal sclerite, 1.6× the width of sternites 1–5; female 7^th^ spiracle half in/half out of tergite; anterior sclerite of female sternite 7 rectangular, w/l ratio: ~ 5.3 (Figs 13, 14); posterior sclerite of female sternite 7 large, weakly sclerotised and rectangular with more sclerotised anterolateral sections; very elongate female cercus with l/w ratio: ~ 5.1; pentagonal subanal plate; smooth spermathecae with some tiny pustules and small dimple surrounded by ridge-like ring; outer and median arms of surstylus broadly joined, with short broad common base (Figs 21–24); outer arm triangular, apically tapering, base twice as wide as apex, rounded apically, with four or five tubercles; median arm broader and longer than outer arm, parallel-sided, with five or six spinous setae; inner arm half the length of median arm, with apophysis; subepandrial clasper (Figs 25–28) with strong basal constriction, rectangular with lateral apical corner a bit extended and rounded, mesal basal corner square; male cercus (Fig. 17) slender, somewhat broadening from base to apex, without distal lateral extension.

**Figures 9–12.**
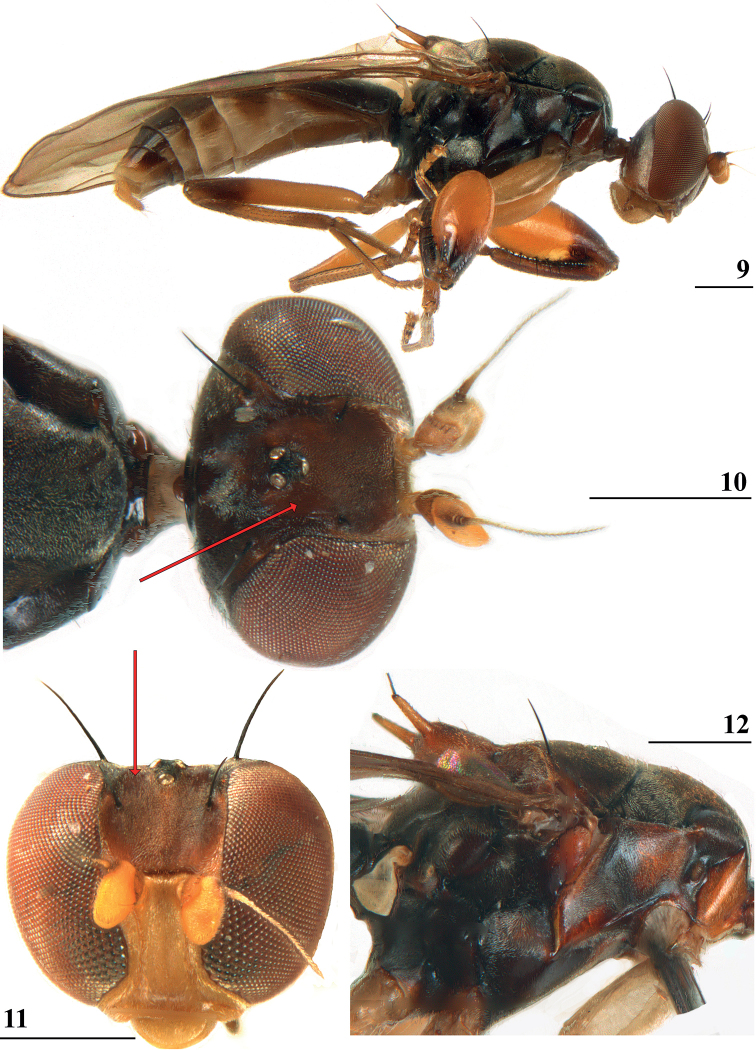
*Centrioncusaberrans***9** Mount Elgon, ♀, habitus, lateral view **10–12** Timboroa Forest, ♂ **10** head, dorsal view **11** head, frontal view **12** thorax, lateral view. Arrows indicate glossy spots on frons. Scale bars: 0.5 mm.

**Figures 13–15.**
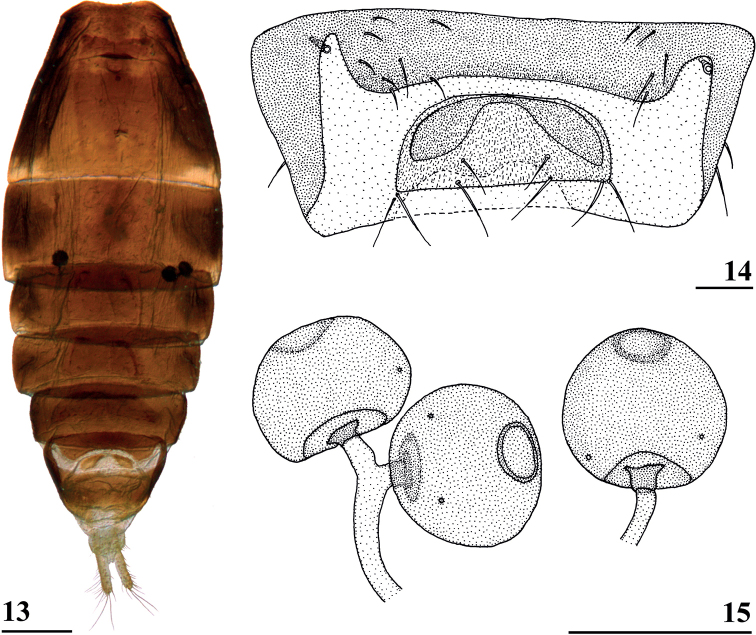
*Centrioncusaberrans*, Mount Elgon, ♀ **13** abdomen, ventral view **14** sternite 7, ventral view **15** spermathecae. Scale bars: 0.5 mm (**13**); 0.1 mm (**14, 15**).

**Figures 16–20.**
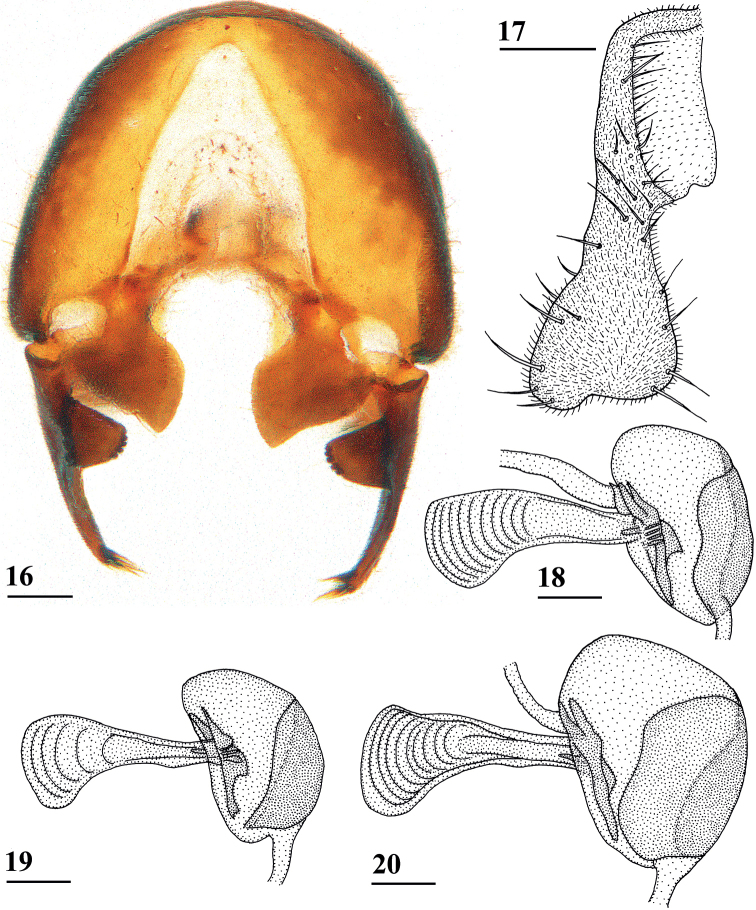
*Centrioncusaberrans*, ♂ **16, 20** Timboroa Forest **17, 18** Mount Elgon **19** Lac Gando **16** epandrium with surstyli, posterior view **17** cercus, posterior view **18–20** ejaculatory apodeme. Scale bars: 0.1 mm.

**Figures 21–24.**
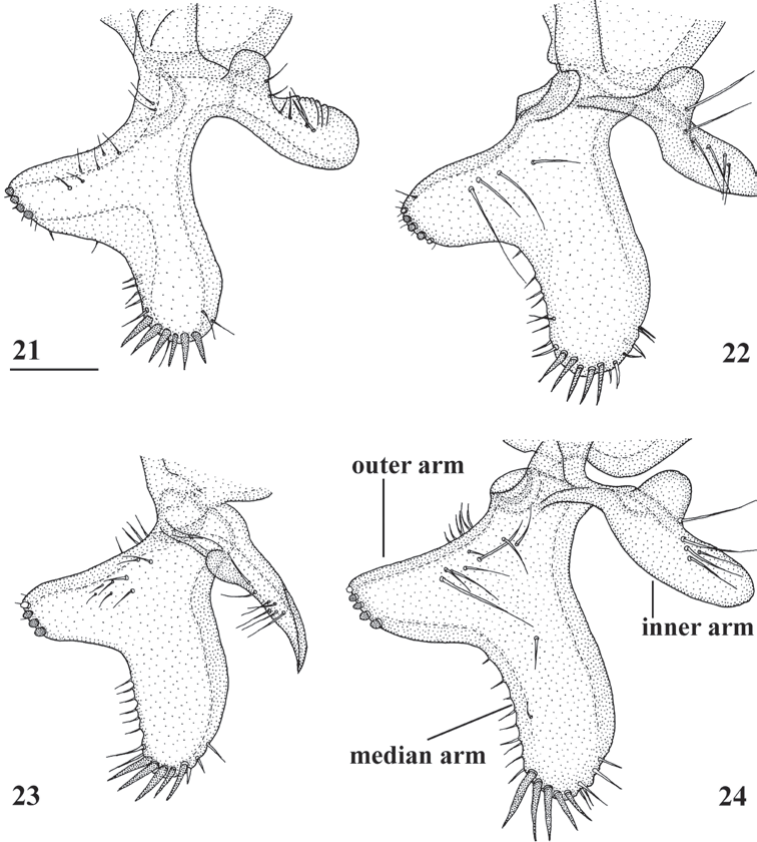
*Centrioncusaberrans*, ♂, surstylus, inner view **21** paratype. Uganda. Kilembe **22** Kenya, Mount Elgon **23** Rwanda, Lac Gando **24** Kenya, Timboroa Forest. Scale bar: 0.1 mm.

**Figures 25–28.**
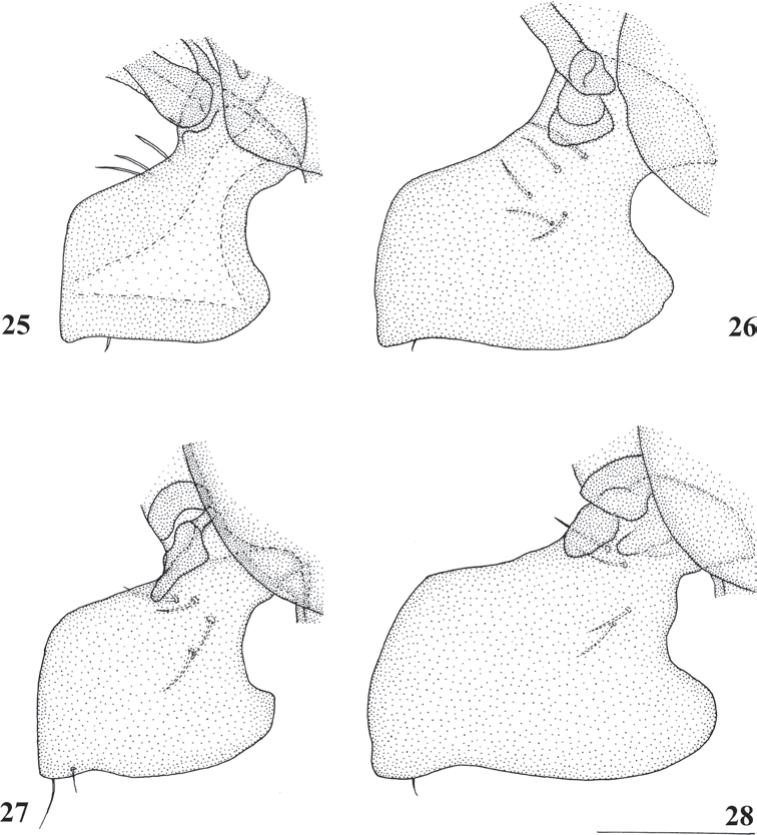
*Centrioncusaberrans*, ♂, subepandrial clasper, outer view **25** paratype, Uganda. Kilembe **26** Kenya, Mount Elgon **27** Rwanda, Lac Gando **28** Kenya, Timboroa Forest. Scale bar: 0.1 mm.

#### Supplementary description.

Below, biometrical data are given for the much larger series now studied, as compared to the type series. Additional morphological data, as well as some rectifications, are presented.

***Measurements*.** For the small type series of 1 ♀ and 2 ♂ Feijen (1983) gave for width of head in ♀ 1.12 mm and in ♂ 1.24 and 1.15 mm, body length in ♀ 4.9 mm and in ♂ 5.2 and 5.0 mm, wing length in ♀ 4.4 mm and in ♂ 4.7 and 4.4 mm, and length of scutellar spine in ♀ 0.26 mm and in ♂ 0.27 and 0.26 mm. In Table 2, measurements and other quantitative characters are presented for the much larger series now studied. In this table, data are presented for females and males separately. From this table, the differences between females and males for quantitative characters are marginal. The body length is slightly larger in the male. In Tables 6, 7 the data for females and males are combined, so that large series can be compared with the other *Centrioncus* species for which large numbers were available. In the other three species for which large series could be measured, the females were, on the average, clearly somewhat larger. The original measurements of the type series of *C.aberrans* fall well within the ranges as presented in Table 2.

**Table 2.** Quantitative characters for *Centrioncusaberrans*. Given are mean ± standard error, range and number of records for females and males separately. Measurements in mm.

Character	♀			♂		
	x̄ ± SE	range	*n*	x̄ ± SE	range	*n*
head width	1.18 ± 0.02	1.11–1.25	8	1.20 ± 0.01	1.13–1.25	15
body length	5.07 ± 0.06	4.82–5.37	8	5.21 ± 0.05	4.94–5.55	15
wing length	4.74 ± 0.04	4.58–4.88	8	4.71 ± 0.05	4.27–4.88	15
sc. sp. length	0.28 ± 0.00	0.27–0.29	8	0.29 ± 0.00	0.25–0.31	15
apical seta length	0.32 ± 0.00	0.30–0.33	7	0.32 ± 0.01	0.29–0.34	3
scutellum length	0.37 ± 0.01	0.36–0.39	7	0.36 ± 0.00	0.34–0.39	11
head w./body l.	0.23 ± 0.00	0.23–0.24	8	0.23 ± 0.00	0.22–0.25	15
sc. sp. l./body l.	0.056 ± 0.001	0.052–0.057	8	0.056 ± 0.001	0.051–0.062	14
sc. sp. l./sc. l.	0.76 ± 0.01	0.73–0.80	7	0.79 ± 0.01	0.75–0.87	11
ap. seta l./sc. sp. l.	1.12 ± 0.01	1.08–1.18	7	1.16 ± 0.01	1.14–1.17	3
F1 – ratio l/w	2.75 ± 0.02	2.68–2.82	8	2.75 ± 0.01	2.68–2.84	14
F1 – n tubercles	35.6 ± 0.6	32–41	15	35.3 ± 0.6	29–40	27
F1 – n spinous setae	9.1 ± 0.1	8–10	15	9.0 ± 0.1	8–10	30
F3 – n tubercles	6.5 ± 0.4	4–10	16	6.5 ± 0.3	4–11	30
OVS length	0.36 ± 0.00	0.36–0.37	6	0.35 ± 0.01	0.29–0.39	11
FOS length	0.21 ± 0.01	0.19–0.24	5	0.22 ± 0.01	0.18–0.25	11

***Colour*.** Most of the specimens now studied were not so dark as those of the type series. These latter three specimens were therefore probably somewhat discoloured, a common tendency in the Centrioncinae (Feijen 1983). The overall colour pattern can be seen in Figs 9–12.

***Head*.** Frons dark brown with anterior edge paler brown (in type-series frons almost uniformly brown), pruinose except two small glossy spots on either side of ocellar tubercle (Figs 10, 11); face, gena and mouthparts pruinose, yellowish brown, palpus blackish; occiput uniformly blackish brown in type series (Feijen 1983), dark brown with more yellowish-brown ventral quarter in additional specimens; length of outer vertical seta 0.36 mm ± 0.00 (*n* = 17); length of fronto-orbital seta 0.22 mm ± 0.01 (*n* = 16).

***Thorax*.** Collar glossy blackish brown (Fig. 10); scutum blackish brown, densely pruinose, humeral calli (postpronotal lobes) less pruinose (Figs 9, 12); scutellum pruinose, dark brown in “greasy” specimens but brown with two vague darker spots in other specimens; scutellar spines brown; pleura (Fig. 12) mostly blackish brown, propleuron, anterior half and posteroventral corner of anepisternum and dorsal “knob” of anepimeron chestnut brown (pleura uniformly blackish brown in type series); scutellar spines diverging at angle of ~ 45° (type-series 40°); scutellar spine/scutellum ratio: 0.78 ± 0.01 (*n* = 18); scutellar spine/body length ratio: 0.056 ± 0.001 (*n* = 22); apical seta/scutellar spine ratio: 1.13 ± 0.01 (*n* = 10); scutellar length/scutellar width (at base) ratio: 0.64.

***Wing*.** Subcostal cell not visible in most specimens, visible in one specimen from Rwanda and one specimen of Mt. Elgon; vein CuA+CuP from vein CuP onward slightly curving downward under angle of 30° to wing margin (Fig. 1); central wing spot distinct, medium-sized, largely in basal quarter of cell r4+5, extending into cells br and bm+dm; vague infuscation along vein M4; cell cua in between triangular and rectangular (Fig. 1).

***Legs*.** Fore femur (Fig. 9) yellowish with distal quarter on both sides dark brown (only Lac Gando specimen on inner side with distinct brown stripe on distal third); fore femur strongly incrassate, l/w ratio: 2.75 ± 0.01 (*n* = 22), two rows of spinous setae on distal two-thirds with 9.0 ± 0.1 setae (*n* = 45), inner row with 4.9 ± 0.0 setae and outer row with 4.1 ± 0.0 setae, two rows of tubercles on distal three-quarters with 35.4 ± 0.5 tubercles (*n* = 42), inner row with 17.2 ± 0.2 tubercles and outer row with 18.2 ± 0.3 tubercles; hind femur distally with 6.5 ± 0.2 (*n* = 46) tubercles in single row but in two specimens one tubercle placed in second row; setal formula (sensu Feijen 1983) of 4.1, 4.9, 18.2, 17.2, 6.5 vs. formula of type-series: 4.0, 5.2, 16.8, 15.2, 6.5.

***Preabdomen*.** Dorsally dark brown, thinly pruinose; posterolateral corners of tergite 2 paler brown; sternites 1–6 brown, thinly pruinose, sternite 2 more glossy; sternite 1 rectangular, constricted on meson (Fig. 13); sternite 2 anteriorly with mesally narrow, strongly sclerotised intersternite 1–2, laterally with thin extensions connected to main sternite 2; sternites 3 and 4 rectangular (Fig. 13), sternite 5 trapezoidal, broadening posteriorly; sternite 6 short, broad, trapezoidal, broadening posteriorly, ~ 1.6× width of sternites 1–5.

***Female postabdomen*.** Seventh spiracle half in/half out of tergite or touching tergite (Fig. 14); anterior sclerite of sternite 7 with w/l ratio: ~ 5.3 (5.2–5.4); flies from Mt Elgon with weakly sclerotised rectangular plate (with rounded anterior corners) of posterior sclerite of sternite 7, as in female paratype with two large more sclerotised sections joined anteromesally (Figs 13, 14); spermathecae as in paratype (spherical with small apical dimple with small ring); junction of ducts of paired spermathecae T-shaped (Figs 13, 15).

***Male postabdomen*.** Epandrium yellowish brown, darker brown anteriorly (Fig. 16) (uniformly brown in type series); qualitative characters conform with original description; surstylus of paratype ♂ (Fig. 21) identical to specimens from Mt Elgon, Lac Gando and Timboroa forest (Figs 22–24); narrow cercus, without apical lateral extension, length/greatest width ratio: 2.6 (Fig. 17, Table 8); subepandrial clasper of males from three localities (Figs 26–28) similar to paratype (Fig. 25); epandrial fold (periandrial fold sensu Feijen (1983)) consists of one single piece, with inner section of fold not detached to form epandrial sclerite (stated as detached in the original description); quantitative characters agree well; outer arm of surstylus with 4–5 tubercles in type-series and specimens from Kenya and Rwanda (Figs 21–24); median arm of surstylus with 6 stout spinous setae in type series, and only 5 spinous setae in specimens from Mount Elgon, Timboroa, and Rwanda (Figs 21–24); basal setulae on outer arm and setulae on inner arm distinctly larger in Kenya specimens (Figs 22, 24); ejaculatory apodeme + sac in all four males (from Rwanda, Mount Elgon, and Timboroa Forest) (Figs 18–20, Table 9) very large (9.3, 10.1, 10.1 and 11.2% of body length, respectively) (shorter in paratype).

#### Distribution and habitat.

**Figure 29.**
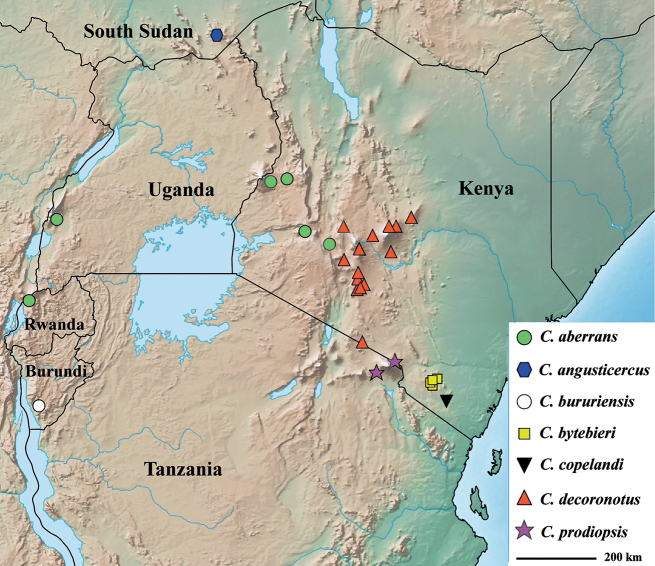
Distribution map of the seven Eastern African *Centrioncus* species.

In the map of Eastern Africa (Fig. 29), the collection localities for *C.aberrans* are indicated. The eastern branch of the Great Rift Valley appears to form a barrier between the populations of *C.aberrans* and *C.decoronotus*. The two females collected in DR Congo/Rwanda are not indicated on the map. These two flies were collected by canopy fogging of individual trees. The methodology used for this fogging is explained in Wagner (2000, 2001). Funnel-shaped sheets were attached to the trees at waist height for collecting the dropping insects. Dr Wagner (pers. comm.) explained that “The lowest branches hit by the fog were about 3 metres [high] and it reaches usually ≤ 10 m.”

**Figure 30.**
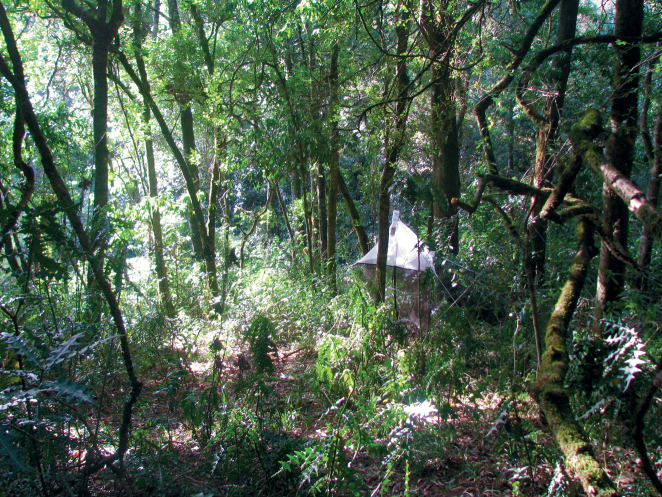
*Centrioncusaberrans*, Kenya, Timboroa Forest, collection site, 2011. Photograph by A.H. Kirk-Spriggs.

Feijen (1983) indicated the habitat for *Centrioncus* and *Teloglabrus* was low shrubs and herbaceous plants. We collected the flies at heights of ~ 20–60 cm from ground level. The results of the canopy fogging trials possibly indicate that *Centrioncus* flies might also occur at higher levels in the trees, though disturbance of the flies or descending insecticide might form alternative explanations. Nothing is known about where oviposition occurs and larval feeding, so the possibility of breeding in the tree canopy cannot be excluded. The flies of *C.aberrans* from Timboroa Forest in Kenya were collected in a malaise trap. The height at which these flies were trapped is more in line with the habitat indicated by Feijen (1983). The Timboroa habitat is illustrated in Fig. 30.

#### Remarks.

Given the unusually large range of distribution of this *Centrioncus* species, special care was taken to examine and compare defining characters such as surstylus, subepandrial clasper, male cercus and female sternite 7 for flies from the regions involved: western Uganda, western Rwanda, western and more central Kenya (Fig. 29). Comparisons of surstyli (Figs 21–24) and subepandrial claspers (Figs 25–28) lead to the conclusion that they are largely similar. For the surstyli, the general shape, number of tubercles and number of spinous setae correspond well. Also, the very specific female sternite 7 (Figs 13, 14) is very similar to the female paratype. The subepandrial clasper of the paratype (Fig. 25) appears less broad, but this is caused by it being slightly tilted to the right during the preparation. While comparing the shapes of the surstyli, care must be taken to present these three-dimensional structures as much as possible in the same plane. In the drawing of the surstylus of the paratype male (Fig. 21), the median arm is slightly tilted upward, so this arm appears somewhat shorter. Likewise, the inner arm of the surstylus in the Lac Gando specimen (Fig. 23) is somewhat tilted and so appears a bit different. In addition, small differences in shape of the three arms and size of setulae are observed and it could be argued that this species is in a very early phase of allopatric speciation. However, if one looks at the major differences for these defining characters between the *Centrioncus* species (Figs 51, 69, 88, 122, 138, 150; Feijen 1983), the conclusion can be drawn that all specimens examined across the large distribution range belong to one species, *C.aberrans*.

### ﻿ Centrioncus angusticercus

Taxon classificationAnimaliaDipteraDiopsidae

Feijen

83F45E8B-D4CA-52FE-AF50-533A91C0C1EC

**Figures 31, 32.**
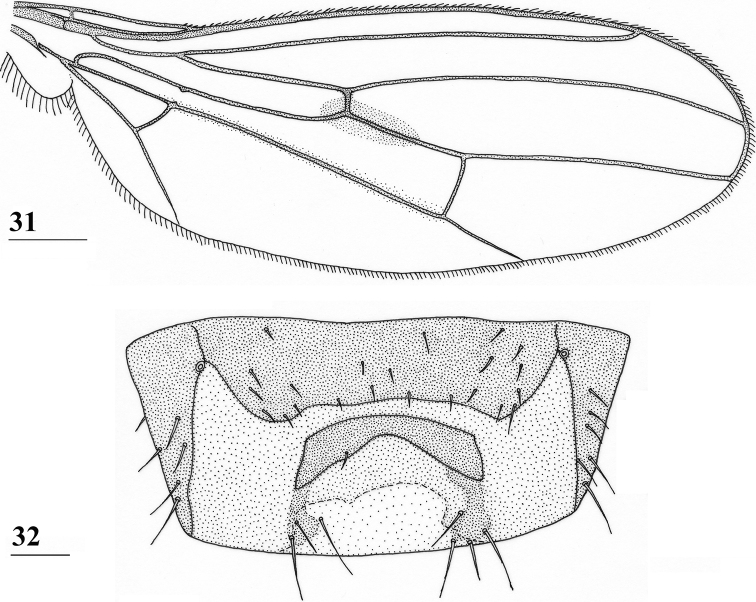
*Centrioncusangusticercus*, ♀, holotype, Nagichot, South Sudan **31** wing, dorsal view **32** sternite 7, ventral view. Scale bars: 0.5 mm (**31**); 0.1 mm (**32**). Both drawings were processed based on original pencil drawings from Feijen (1983: figs 13, 27).

Figs 31 , 32 , Table 8

Centrioncus angusticercus Feijen, 1983: 75.

#### Type material.

South Sudan, ***holotype***, ♀, Nagichot, [4°16'37.99"N, 33°33'34.99"E, 1980 m], D.J. Lewis (NHMUK) (not examined, but notes and pencil drawings for Feijen (1983) were used).

#### Diagnosis

**(after Feijen 1983).***Centrioncusangusticercus* can be recognised by its pruinose, mesally slightly depressed frons with two glossy spots; dark brown collar; blackish brown scutum; blackish brown scutellum with pale brown lateral sides and spines; pleura blackish brown except for brown propleuron and anterior anepisternum; apical seta/scutellar spine ratio: > 1.0; pale brown fore femur with distal third on inner side dark brown and small dark spot apically on outer side, with 36 tubercles; small round central wing spot (Fig. 31) around junction of crossvein r-m and vein M1 in tip of cell br and base of cell r4+5 slightly extending into cell bm+dm; tergites blackish brown, apical edges paler; female 7^th^ spiracle just in tergite; anterior sclerite of female sternite 7 rectangular, w/l ratio: ~ 4.4; posterior sclerite of female sternite 7 consisting of well-sclerotised trapezoidal anterior plate and two weakly sclerotised posterolateral extensions (Fig. 32), anterior edge of anterior plate parallel to its posterior edge, short lateral sides straight, slightly broadening posteriorly, posterior side concave, weakly sclerotised posterolateral extensions irregularly shaped and posteriorly with 6–8 setulae; very elongate female cercus with l/w ratio: 5.4 (Table 8); pentagonal subanal plate; smooth round spermathecae with some tiny pustules and small dimple surrounded by ridge-like ring.

#### Supplementary description.

***Wing*.** Small, rounded, elongate central wing spot (Fig. 31) around junction of crossvein r-m and vein M1, in posterior part of tip of cell br and posterior part of base of cell r4+5 to halfway to crossvein dm-m, slightly extending into cell bm+dm; very vague infuscation along vein M4 between cell cua and crossvein dm-m; vein CuA+CuP from vein CuP onward extending under an angle of 25° to wing margin in straight line; vein M4 continuing distally from crossvein dm-m in straight line to wing margin.

***Female postabdomen*.** Anterior sclerite of sternite 7 rectangular, tapering posteriorly, lateral sides convex, w/l ratio: ~ 4.4 (Fig. 32, Table 8); posterior sclerite of sternite 7 consisting of well-sclerotised trapezoidal anterior plate and two weakly sclerotised posterolateral extensions (Fig. 32); anterior edge of anterior plate parallel to posterior edge of anterior sclerite, short lateral sides straight, slightly expanded posteriorly, posterior edge concave; weakly sclerotised posterolateral extensions irregularly shaped and posteriorly with 3–4 pairs of setulae; very elongate female cercus with l/w ratio: 5.4 (Table 8).

#### Distribution and habitat.

In the Shillito archive (now in NHMUK), a letter was found written on 22 January 1950 by D.J. Lewis, the collector of the holotype, and addressed to J.F. Shillito. It provided information on the collecting locality of the single known specimen: “The Diopsid, *Centrioncusprodiopsis* Speiser, was taken at an altitude of about 1980 metres among vegetation near a small stream in a patch of forest. Nagichot is on the Didinga Hills, one of the many isolated ranges, and may be considered as part of the Eastern and Southern zoogeographical Province ...” The type locality is shown on the map for Eastern Africa (Fig. 29).

#### Remarks.

Feijen (1983) described the posterior sclerite of sternite 7 as “very roughly U-shaped with eight hairs, anterior edge parallel to posterior edge of sternum 7, anterior section well sclerotized, but arms of the U connecting to posterior hairs of sclerite weakly sclerotized.” However, the view that this sclerite can be described as “U-shaped” is now rejected (compare Fig. 32 vs. Figs 43, 62, 82, 119, 133, 148). *Centrioncusangusticercus* is now placed outside the group of *Centrioncus* with a U-shaped sclerite. Instead, it is considered as closer to the other *Centrioncus* without a U-shaped sclerite: *C.aberrans* and *C.crassifemur* sp. nov. Other similarities like l/w ratio of ♀ cercus and w/l ratio of ♀ anterior sternite 7 also support this group. For a confirmation of this relationship, the discovery of the male sex and study of the male genitalia of *C.angusticercus* and *C.crassifemur* sp. nov. and molecular analyses will be required.

### ﻿ Centrioncus bururiensis sp. nov.

Taxon classificationAnimaliaDipteraDiopsidae

5B7FDC48-79D5-5A51-8A0D-853C0FD4BCC0

https://zoobank.org/FF6069BA-459B-4D00-80A6-E1CB6C2CA2C1

Figs 2 , 29 , 33–36 , 37–40 , 41–43 , 44–49 , 50–54 , Tables 8 , 9

#### Type material.

Burundi, ***holotype***, ♀, Bururi Nat. Forest, 3°55'49"S, 29°37'1"E, 1955 m, 7–21.ix.2010, R. Copeland, malaise trap, indigenous forest near stream (NMKE). ***Paratypes***: 1 ♀, 1 ♂, same data as holotype (ICIPE, RMNH).

#### Diagnosis.

*Centrioncusbururiensis* sp. nov. can be recognised by the mesally slightly depressed, uniformly pruinose frons; mainly glossy collar; pruinose blackish brown scutum; pruinose, blackish brown scutellum with brown spines; pleura blackish brown with ventral edge of proepimeron, dorsal third of anepisternum, posterior quarter of anepimeron, and greater ampulla brown; scutellar spine/scutellum ratio: 0.8–0.9; apical seta/scutellar spine ratio: 0.94–1.18; pale brown, strongly incrassate fore femur 1 (l/w ratio: 2.63–2.93) with ~ 38.5 tubercles, dark apical third on mesal side; small central brownish wing spot around crossvein r-m in distal tip of cell br and basal quarter of cell r4+5, somewhat extending into cell bm+dm (Fig. 2); tergites blackish brown, thinly pruinose; sternites 4 and 5 rectangular with each a pair of small, heavily sclerotised areas basally; sternite 6 short, almost as broad as segment, laterally tapering; female 7^th^ spiracle in membrane; anterior sclerite of female sternite 7 rectangular, w/l ratio: ~ 2.6 (Figs 42, 43); posterior sclerite of female sternite 7 broad, strongly curved, U-shaped, well sclerotised (Figs 42, 43), posterolateral apices tapering; elongate female cercus with l/w ratio: ~ 4.5; subanal plate pentagonal, laterally rounded and apically tapering into short extension; spermathecae heavily sclerotised, smooth, flattened (Fig. 49), rounded in cross section, with small apical dimple; outer and median arms of surstylus broadly joined, with short broad common base (Fig. 51); outer arm triangular, apically 4 tubercles; median arm very broad, parallel-sided, apically 11 stout, spinous setae; inner arm half the length of median arm, with apophysis; subepandrial clasper (Fig. 53) with long strong basal constriction, extended towards meson, rectangular with apical corners square, mesal basal corner rounded; male cercus (Fig. 54) slender, distinctly broadening in apical third but without distal lateral extension.

#### Description.

***Measurements*.** Body length ♀ 5.8, 5.8 mm, ♂ 4.6 mm, width of head ♀ 1.28, 1.27 mm, ♂ 1.07 mm, wing length ♀ 5.3, 5.2 mm, ♂ 4.1 mm, length of scutellar spine ♀ 0.39, 0.36 mm, ♂ 0.27 mm.

***Head*.** Frons slightly depressed mesally (Fig. 34), dark brown but anterolateral margins pale brown, uniformly thinly pruinose; posterior side of head dark brown with paler brown ventral margin (Fig. 36), pruinose especially posterior to ocellar tubercle and near eye margins; face yellowish brown, thinly pruinose but densely pruinose along eye margins (Figs 34, 35); antenna yellowish brown but dorsal edge of funiculus darker (Figs 34–36, 38); maxillary palpus dark (Figs 34, 35); outer vertical seta in ♀ 0.41 mm and in ♂ 0.31 mm, fronto-orbital setae in ♀ 0.29 mm and in ♂ 0.18 mm (Figs 34, 36).

**Figures 33–36.**
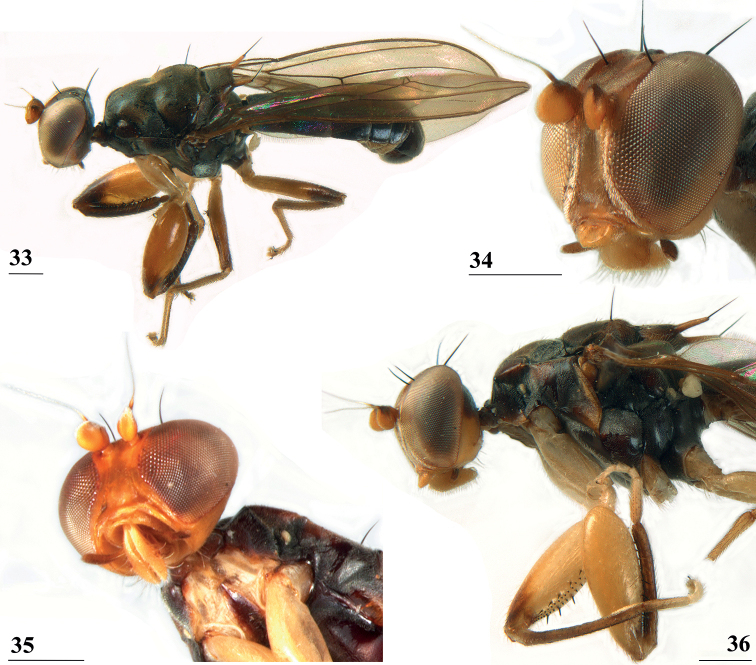
*Centrioncusbururiensis* sp. nov., Bururi Nat. Forest **33** ♂, paratype, habitus, dorsolateral view **34–36** ♀, paratype **34** head, anterolateral view **35** head, basiliform prosternum, ventral view **36** head, thorax, fore femora, lateral view. Scale bars: 0.5 mm.

***Thorax*.** Collar glossy dark brown, posterior and lateral edges pruinose; scutum blackish brown and pruinose (Figs 33, 36), tiny pale spot around base of intra-alar seta, humeral callus brown and less pruinose; scutellum dark brown, pruinose, scutellar spines brown (Figs 33, 36); pleura blackish brown with ventral edge of proepimeron, dorsal third of anepisternum, posterior quarter of anepimeron, and greater ampulla brown (Fig. 36), but, like in all *Centrioncus*, paler sections can also turn dark (Fig. 33); pleura mainly pruinose, glossy sections include ventral edge of anepisternum and anterior spot on katepisternum (Fig. 36); posterior notopleural seta, supra-alar and infra-alar setae present, infra-alar seta longest, followed by posterior notopleural seta, supra-alar seta on inconspicuous carina; basiliform prosternum lanceolate and sharply pointed anteriorly (Fig. 35); scutal length/scutal width ratio: 1.0 in ♀ and ♂; scutellum sticking up at an angle of just over 20° from body axis; scutellar spines almost aligned with dorsal plane of scutellum (Fig. 36), diverging at angle of ~ 50°; scutellar spine/scutellum ratio: 0.9 in ♀, 0.8 in ♂; scutellar spine/length of body ratio: 0.06–0.07 in ♀, 0.06 in ♂; apical seta/scutellar spine ratio: 0.94 in ♀, 1.18 in ♂, scutellar length/scutellar width (at base) ratio: ~ 0.70.

***Wing*.** Almost transparent but slightly tinged; small, central brownish spot around crossvein r-m in distal tip of cell br and basal quarter of cell r4+5, vaguely extending into cell bm+dm (Fig. 2); some vague infuscation around vein M4 proximally of crossvein dm-m; glabrous basal areas only include cells bc and c and basal third of cell br; crossvein h distinct; cell sc almost closed; vein CuA+CuP from vein CuP onward extending under angle of 30° to wing margin in almost straight line (Fig. 2); vein M4 continuing distal of crossvein dm-m very slightly turning downwards towards wing margin; cell cua triangular; alula distinct; crossvein bm-m hardly indicated.

**Figures 37–40.**
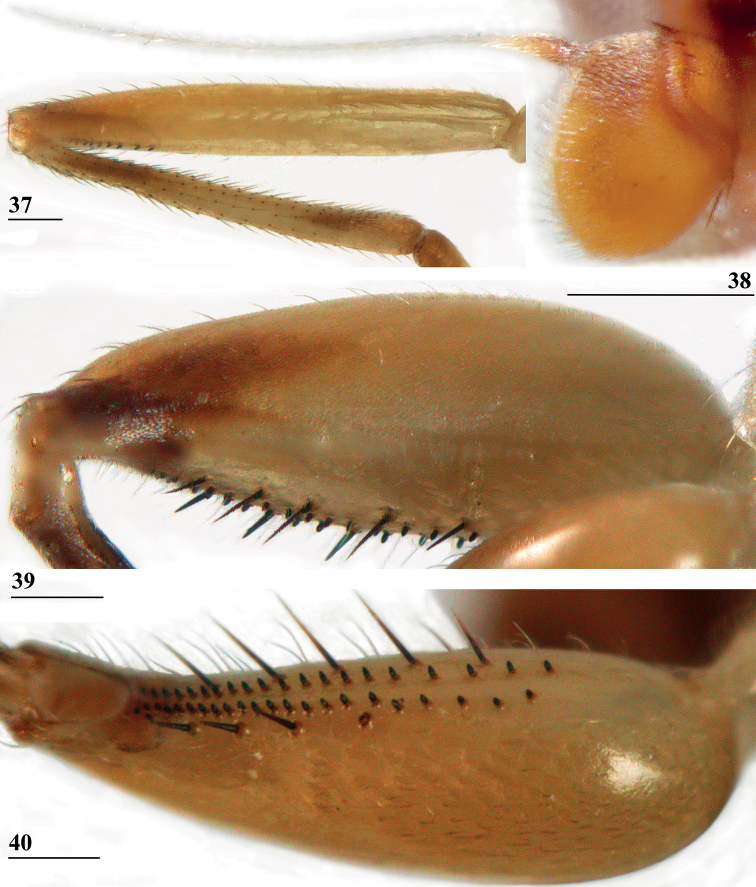
*Centrioncusbururiensis* sp. nov., Bururi Nat. Forest **37, 39, 40** ♀, holotype **38** ♀ paratype **37** hind femur, outer view **38** antenna, lateral view **39** fore femur, inner view **40** fore femur, ventral view. Scale bars: 0.2 mm.

***Legs*.** Coxa 1 and trochanter 1 whitish, thinly pruinose (Figs 35, 36); femur 1 glossy, yellowish brown with dark brown spot on apical third of inner side, darker in ♂ and extending to outer side (Figs 33, 36, 39); tibia 1 dark brown, pruinose; metatarsus 1 yellowish, other tarsal segments whitish; mid and hind legs pale yellowish; femur 2 with pale brownish apex; outer apical quarter of femur 3 with brown spot (Fig. 37); femur 1 strongly incrassate in ♀ and ♂ (Figs 33, 36, 39, 40), l/w ratio in ♀: 2.87, 2.93 and in ♂: 2.63, inner rows of spinous setae (Figs 39, 40) with 4.8 setae (*n* = 6, range 4–5), outer rows with 4.0 setae (*n* = 6, range 3–5), inner rows of tubercles with 18.3 tubercles (*n* = 6, range 17–20), outer rows with 20.2 tubercles (*n* = 6, range 18–23); femur 3 (Fig. 37) with 6.8 distal tubercles (*n* = 4, range 5–8); setal formula 4.0, 4.8, 20.2, 18.3, 6.8; no apical spurs on femora 2 and 3.

**Figures 41–43.**
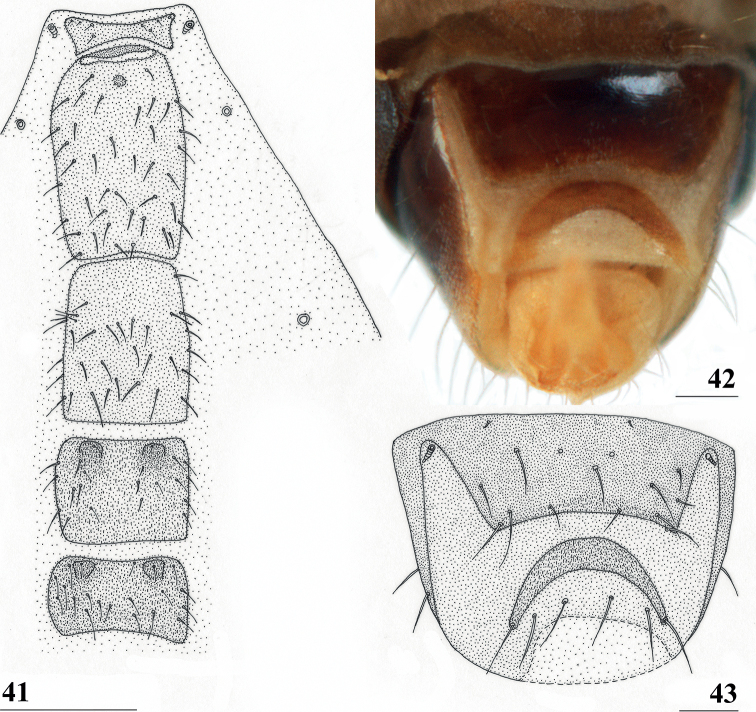
*Centrioncusbururiensis* sp. nov., Bururi Nat. Forest **41, 43** ♀, paratype **42** ♀, holotype **41** sternites 1–5, ventral view **42, 43** sternite 7, ventral view. Scale bars: 0.5 mm (**41**); 0.1 mm (**42, 43**).

***Preabdomen*.** Tergites blackish brown, very thinly pruinose; sternites 1–5 yellowish brown, sternites 1–3 glossy and clothed with some setulae (Fig. 41), sternites 4–5 thinly pruinose, with some setulae; sternite 1 very short, constricted medially; sternite 2 anteriorly with small more sclerotised sclerite (plesiomorphic equivalent of intersternite 1–2 found in most Diopsidae), laterally thinly connected to main sternite, separated from main sclerite by short, non-sclerotised “gap” (Fig. 41); sternites 2–5 rectangular, slender, more or less equal in width (Fig. 41), sternite 3 20% shorter than sternite 2, sternite 4 only half length of sternite 2 and sternite 5 only one-third length of sternite 2; sternites 4 and 5 both with pair of small, heavily sclerotised sections anteriorly (Fig. 41); sternite 6 short and broad, laterally tapering, almost as broad as segment, 1.6× as wide as sternites 1–5 (Fig. 42).

**Figures 44–49.**
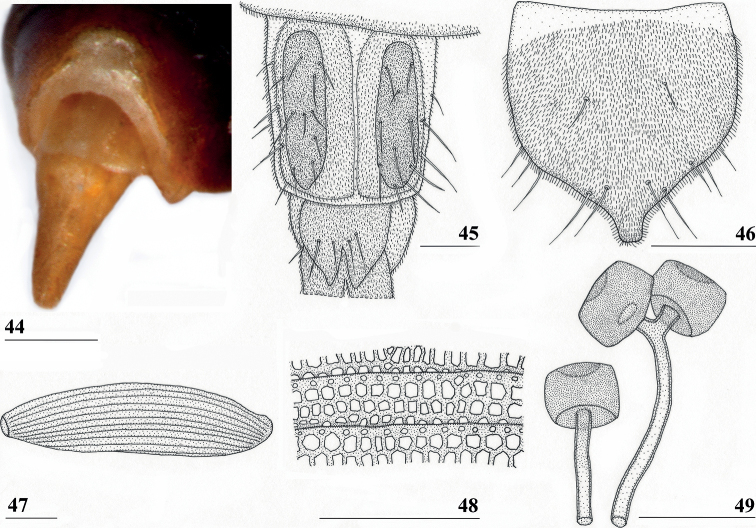
*Centrioncusbururiensis* sp. nov., Bururi Nat. Forest **44** ♀, holotype **45–49**, ♀, paratype **44** tergite 7, 8, 10, cerci, dorsal view (setulae not shown) **45** tergite 8, 10, basal cerci, dorsal view **46** subanal plate, ventral view **47** egg **48** egg, detail **49** spermathecae. Scale bars: 0.2 mm (**44, 47**); 0.1 mm (**45, 46, 48, 49**).

***Female postabdomen*.** Tergite 7 (Figs 42, 44) brown, with large mesal gap posteriorly, lateral edges hardly curved under ventrally; suture in basal ring of segment 7 hardly visible; 7^th^ spiracle in membrane (Fig. 43); anterior sclerite of sternite 7 rectangular, slightly tapering posteriorly (Figs 42, 43), w/l ratio: ~ 2.6 (Table 8), glossy brown, with ~ 16 setulae and some microtrichia only on posterior edge; posterior sclerite of sternite 7 broad, strongly curved U-shaped (Figs 42, 43), almost forming semicircle, posterolateral apices tapering, well sclerotised, clothed in microtrichia and with one apical setula; tergite 8 consisting of two oblong plates, well separated on meson (Fig. 45); sternite 8 consisting of two oblong plates; tergite 10 (Fig. 45) somewhat onion-shaped, extending posteriorly between cerci, clothed in microtrichia and a few setulae; cercus elongate, somewhat tapering posteriorly (Fig. 44; cut off in paratype, Fig. 45), l/w ratio: ~ 4.5; subanal plate (Fig. 46) somewhat pentagonal, laterally rounded and apically tapering into short extension, clothed in microtrichia except anterior edge, with ~ 12 setulae especially along posterior edge; spermathecae heavily sclerotised, smooth, flattened (Fig. 49), round in cross section, with small apical dimple; junction of ducts of paired spermathecae V-shaped (Fig. 49).

**Figures 50–54.**
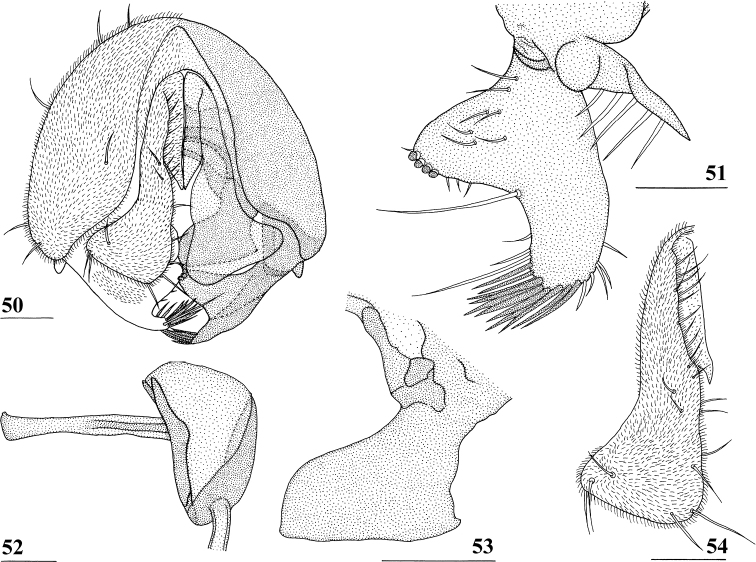
*Centrioncusbururiensis* sp. nov., ♂, paratype, Bururi Nat. Forest **50** epandrium, cerci, surstyli, posterior view **51** surstylus, inner view **52** ejaculatory apodeme + sac **53** subepandrial clasper, posterior view **54** cercus, posterior view. Scale bars: 0.1 mm.

***Male postabdomen*.** Tergite 6, sternite 8 and epandrium more brownish than blackish brown tergites 1–5; sternite 6 rectangular, almost as broad as tergite 6; epandrium broad and rounded (Fig. 50) with w/l ratio: 1.31, dark brown but paler brown along inner edges, clothed in microtrichia and with ~ 6 small setulae; surstylus in outer view (Fig. 50) not deeply bifurcated, with central patch of microtrichia on outer side; outer and median arms broadly joined, with short broad common base (Fig. 51); outer arm almost triangular, tapering apically, not constricted at base, with four apical, closely joined tubercles, basally to centrally with seven setulae on inner side and a few small setulae along posterior edge; median arm very broad, parallel-sided, with apically 11 stout, dark, spinous setae, some small pale setulae and two long setulae (Figs 50, 51); inner arm linked to base of other arms, half-length of median arm, tapering apically, with one apophysis and five strong setulae; subepandrial clasper (Fig. 53) with long strong basal constriction, extended mesally, rectangular with apical corners almost square and mesal basal corner rounded, glabrous; cercus (Fig. 54) slender, distinctly broadening in apical third, without distal lateral extension, clothed in microtrichia, with short setulae mainly along mesal edge, length/greatest width ratio: 2.4 (Table 8); phallapodeme short; ejaculatory apodeme + sac (Fig. 52) very large, 10.4% of body length (Table 9), ejaculatory apodeme club-shaped, basally slender.

***Egg.*** ♀ paratype contained 45 developing and developed eggs in abdomen. The eggs (Figs 47, 48) measured ≤ 1.10 mm in length, with slightly elevated longitudinal ridges spanning from anterior pole to posterior pole with fine, nearly hexagonal microstructure between ridges (Fig. 48).

#### Distribution and habitat.

The new species is only known from the Bururi National Forest in Burundi. It was collected at an altitude of 1955 m. This small Afromontane Forest is relatively isolated from other similar forests. The type locality is shown on the map for Eastern Africa (Fig. 29).

#### Etymology.

The specific epithet of *C.bururiensis* sp. nov. refers to the place of origin for the holotype, the Bururi Forest in Burundi.

#### Remarks.

While cursorily examining the three flies of the Bururi Forest, the male fly appeared to be *Centrioncusaberrans*, given the apparently similar shapes of surstyli and cerci. However, the females with their clearly visible curved posterior sclerite of sternite 7 (Figs 42, 43) did not belong to the *C.aberrans* species-group. This led to the initial assumption that this might be the first case of two *Centrioncus* species occurring sympatrically. However, closer examination of the male genitalia revealed distinct differences with those of *C.aberrans*. The female is also different from other described *Centrioncus* with the characteristic sternites 4, 5, and 7 and the spermathecae. The subanal plate of *C.bururiensis* sp. nov. is more like those of *C.decoronotus* and *C.prodiopsis*, not like *C.aberrans* (compare Fig. 46 vs. Feijen 1983: figs 49–51). The w/l ratio of the anterior female sternite 7 is like the ones for *C.decellei*, *C.bytebieri* and *C.prodiopsis*, certainly not like the one for *C.aberrans* (Table 8), while the shape of posterior sclerite of sternite 7 is more like the one for *C.decoronotus* and certainly not like *C.prodiopsis* or *C.bytebieri* (compare Figs 119, 148, 62 vs. De Meyer 2004: fig. 6).

It remains puzzling that the male genitalia of *Centrioncusbururiensis* sp. nov. indicates a possible relationship with *C.aberrans*, while the female genitalia contradicts such a relationship. Analyses, including molecular analysis, of a series of fresh material are required to resolve this issue. The number of 45 eggs found in a paratype was quite high. Feijen (1983) found in various *Centrioncus* and *Teloglabrus* species > 20 developing and developed eggs and stated that a fecundity of between 25 and 50 eggs seemed likely. The gravid female paratype was collected in September, coinciding with the start of the short rains in Burundi.

### ﻿ Centrioncus bytebieri

Taxon classificationAnimaliaDipteraDiopsidae

De Meyer

5802125B-692E-54CC-BA05-D6B0740780AF

Figs 3 , 29 , 55–60 , 61–67 , 68–73 , Tables 3 , 6 , 8 , 9

Centrioncus bytebieri De Meyer, 2004: 26; Karanja et al. 2014: 49.

#### Type material.

Kenya, ***holotype***, ♂, Ngangao Forest, Taita Hills [3°21'38"S, 38°20'29"E, 1800 m], M. De Meyer (NMKE). ***Paratypes***: 8 ♀ (1 ♀ as “allotype”), 7 ♂, same data as holotype; 8 ♀, 10 ♂, Chawia Forest, Taita Hills [3°28'56"S, 38°20'25"E]; 5 ♀, 6 ♂, Yale Forest, Taita Hills [3°24'7"S, 38°19'36"E]; 5 ♀, 2 ♂, Mbololo Forest, Taita Hills [3°19'36"S, 38°27'3"E]. All paratypes collected by M. De Meyer at altitudes of 1400–1800 m, allotype NMKE, 2 paratypes NHMUK, 2 paratypes NMSA, 46 paratypes MRAC.

#### Material studied.

Kenya: 2 ♀, Coastal Prov., Taita Hills, Mbololo Forest, malaise trap 3°20.00'S, 38°26.85'E, 1550 m, 29.iii–6.iv.1999, Taita biodiversity project (ICIPE); 1 ♀, 1 ♂, Coastal Prov., Taita Hills, Fururu Forest, malaise trap, 3°25.78'S, 38°20.30'E, 1680 m, 23–29.i.1999, Taita biodiversity project, (ICIPE); 1 ♀, Coastal Prov., Taita Hills, Chawia Forest, malaise trap next to small forest pond, 3.47908°S, 38.34162°E, 1614 m, 9–23.i.2012, R. Copeland (ICIPE); 1 ♀, 4 ♂, Coastal Prov., Taita Hills, Vuria Forest, malaise trap, edge indigenous forest, 3.41428°S, 38.29178°E, 2162 m, 1 ♂ 12–6.vi.2011, 1 ♀ 11–25.i.2012, 1 ♂ 13–27.v.2012, 1 ♂ 25.vii–8.viii.2012, 1 ♂ 8–22.viii.2012, R. Copeland (ICIPE); 5 ♀, 1 ♂, Coastal Prov., Taita Hills, Ngangao Forest, malaise trap, indigenous forest, 3.36100°S, 38.34186°E, 1848 m, 1 ♀ 30.x–13.xi.2011, 2 ♀ 24.i–7.ii-2012, 1 ♀ 8–22.ii.2012, 1 ♂ 19.iv–3.v.2012, 1 ♀ 27.v–10.vi.2012, R. Copeland (ICIPE). In total 10 ♀ and 6 ♂ were studied.

#### Diagnosis.

**Figures 55–60.**
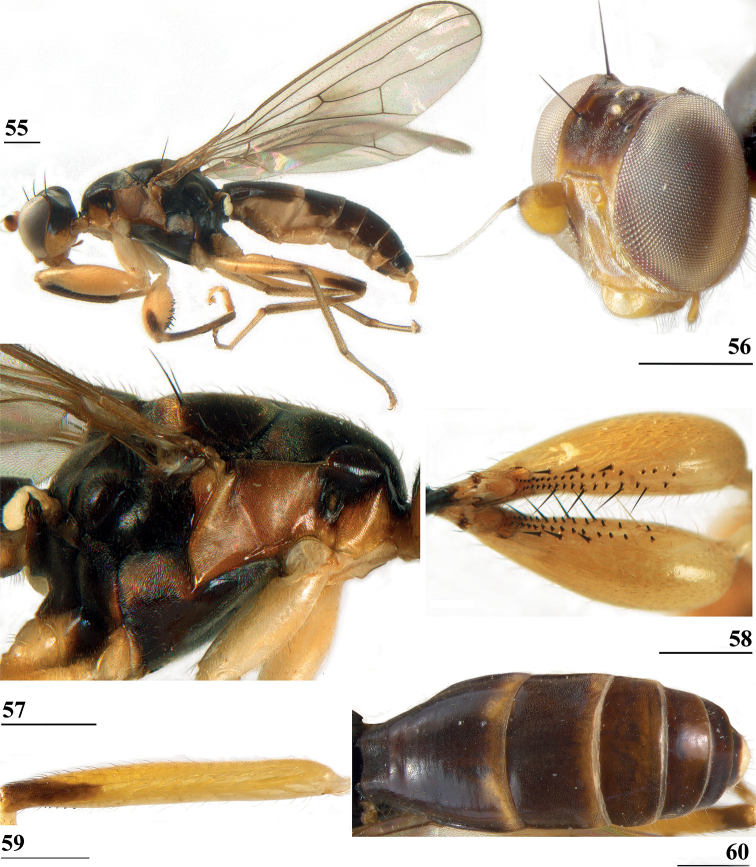
*Centrioncusbytebieri*, ♀ **55, 57–60** Ngangao Forest **56** Vuria Forest **55** habitus, lateral view **56** head, frontolateral view **57** thorax, lateral view **58** fore femora, ventral view **59** hind femur, inner view **60** abdomen, dorsal view. Scale bars: 0.5 mm.

**Figures 61–67.**
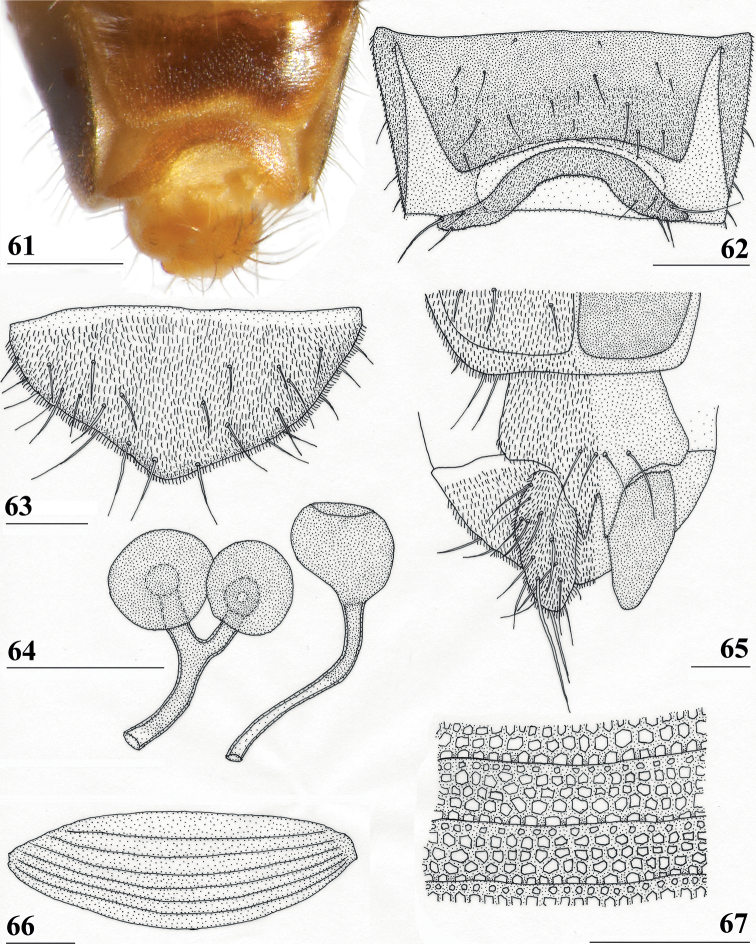
*Centrioncusbytebieri*, ♀ **61** Ngangao Forest **62–67** Vuria Forest **61, 62** sternite 7, ventral view **63** subanal plate ventral view **64** spermathecae **65** sternites 8, 10, cerci, ventral view **66** egg **67** egg, detail. Scale bars: 0.2 mm (**61, 62, 66**); 0.1 mm (**63–65, 67**).

**Figures 68–73.**
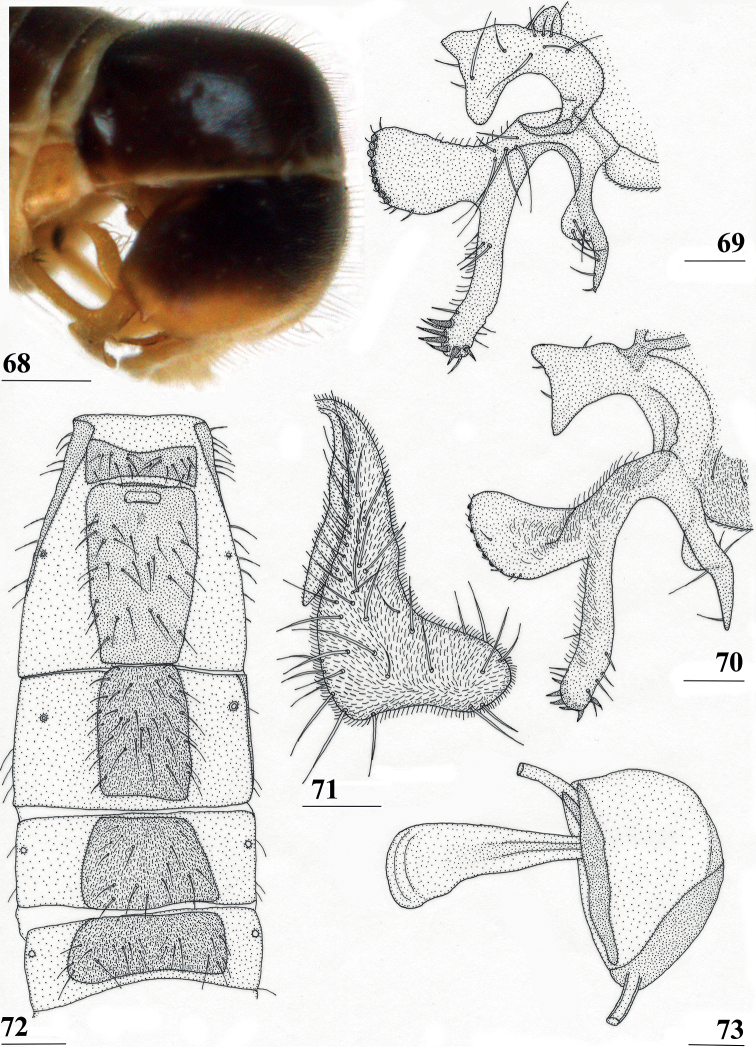
*Centrioncusbytebieri*, ♂, Vuria Forest **68** synsternite 7+8, epandrium, surstyli, lateral view **69** subepandrial clasper and surstylus, inner view **70** subepandrial clasper and surstylus, outer view **71** cercus, posterior view **72** sternites 1–5, ventral view **73** ejaculatory apodeme + sac. Scale bars: 0.2 mm (**68, 72**); 0.1 mm (**69–71, 73**).

*Centrioncusbytebieri* can be recognised by the pruinose frons with glossy spots; glossy collar; pruinose blackish brown scutum; scutellum blackish brown with brown lateral sides; scutellar spines brown; pleura blackish brown with largely brown proepisternum and strikingly brown central area formed by anepisternum, anepimeron and greater ampulla (Figs 55, 57); scutellar spine/scutellum ratio: 0.95; apical seta/scutellar spine ratio: 1.14; pale brown, incrassate fore femur (l/w ratio: ~ 3.30) with ~ 33 tubercles, broad brown stripe on distal half; small, central brownish wing spot around crossvein r-m in distal tip of cell br and basal fifth of cell r4+5, somewhat extending into cell bm+dm (Figs 3, 55); tergites blackish brown, syntergite with small pale spots in posterolateral corners (Fig. 60), tergite 3 with pale posterior band; sternite 4 trapezoidal; sternite 5 rectangular; sternite 6 short and broad; female 7^th^ spiracle in tergite; anterior sclerite of female sternite 7 rectangular, w/l ratio: ~ 2.6 (Figs 61, 62); posterior sclerite of female sternite 7 somewhat U-shaped, with posterolateral extensions almost extending to posterior corners of tergite 7, lateral sections outwardly curved (Figs 61, 62); female cercus rather broad with l/w ratio: 2.3; subanal plate short, broad, triangular with rounded apex (Fig. 63); spermathecae well-sclerotised, smooth, rounded with shallow apical dimple; common base of outer and median arm of surstylus long, slender; outer arm of surstylus basally constricted, apically > 1.5× width at base, with 6–9 tubercles (Figs 68–70); median arm slender, longer than outer arm, with 3–6 spinous setae; inner arm of surstylus two-thirds length of median arm, with apophysis; subepandrial clasper (Figs 69, 70) trapezoidal, basally narrower, mesal apical corner rounded, lateral apical corner pointed; male cercus (Fig. 71) with broad lateral extension, slightly concave apically.

#### Supplementary description.

Below, biometrical data are presented for the series now studied, and compared to the type series. Additional morphological data, as well as a few rectifications, are presented.

***Measurements*.** For the type series of 26 ♀ and 26 ♂, De Meyer (2004) stated body length 5.59 mm (4.95–6.80) and wing length 4.83 mm (4.55–5.10). In Table 3, measurements and other quantitative characters are presented for the series now studied. In this table, data are presented for females and males separately. The table shows that differences between females and males for quantitative characters are small. The body length and various other measurements are slightly larger in females. In Table 6, the data for females and males are combined, so that large series can be compared with other *Centrioncus* species for which large numbers were available. The original measurements of the type series agree well with the means and ranges as presented in the tables: body length 5.55 mm ± 0.09 vs. 5.59 mm and wing length 5.04 mm ± 0.06 vs. 4.83 mm. The measurements confirm *C.bytebieri* as one of the larger *Centrioncus*, together with possibly *C.angusticercus* and *C.bururiensis* sp. nov. (based on measurements presented in this paper (Tables 6, 7), and Feijen (1983)).

**Table 3.** Quantitative characters for *Centrioncusbytebieri*. Given are mean ± standard error, range and number of records for females and males. Measurements in mm.

Character	♀			♂		
	x̄ ± SE	range	*n*	x̄ ± SE	range	*n*
head width	1.18 ± 0.01	1.11–1.21	10	1.13 ± 0.02	1.06–1.22	6
body length	5.77 ± 0.05	5.61–6.16	10	5.16 ± 0.08	4.94–5.49	6
wing length	5.18 ± 0.04	5.00–5.34	10	4.82 ± 0.11	4.39–5.15	6
sc. sp. length	0.39 ± 0.01	0.34–0.43	10	0.31 ± 0.01	0.29–0.34	6
apical seta length	0.43 ± 0.01	0.39–0.46	9	0.37 ± 0.01	0.36–0.39	3
scutellum length	0.40 ± 0.00	0.37–0.42	10	0.34 ± 0.01	0.31–0.36	6
head w./body l.	0.204 ± 0.002	0.192–0.212	10	0.219 ± 0.001	0.215–0.222	6
sc. sp. l./body l.	0.067±0.001	0.060–0.075	10	0.059 ± 0.001	0.055–0.062	6
sc. sp. l./sc. length	0.97 ± 0.02	0.90–1.06	10	0.91 ± 0.01	0.86–0.93	6
ap. seta l./sc. sp. l.	1.13 ± 0.02	1.03–1.19	9	1.20 ± 0.04	1.11–1.25	3
ratio l/wF1	3.25 ± 0.02	3.17–3.39	10	3.39 ± 0.02	3.31–3.43	6
n tubercles F1	31.9 ± 0.7	26–38	19	33.9 ± 1.1	27–38	12
n spinous setae F1	7.2 ± 0.2	6–10	19	6.3 ± 0.2	5–70	12
n tubercles F3	4.8 ± 0.3	3–7	20	5.1 ± 0.2	4–6	12
OVS length	0.39 ± 0.01	0.36–0.43	10	0.36 ± 0.01	0.34–0.39	6
FOS length	0.29 ± 0.01	0.27–0.31	10	0.28 ± 0.01	0.27–0.29	6

***Colour*.**De Meyer (2004) gave a fine description of the colouration. Characteristic colouration of pleura in prime specimens should be stressed (Figs 55, 57). Yellowish-brown sections include proepisternum, proepimeron, anepimeron with greater ampulla, posterodorsal corner of katepisternum and anterior spot on meron.

***Head*.** Frons (Fig. 56) thinly pruinose, laterally densely pruinose, with glossy spots (reduced in some specimens) laterally of ocellar tubercle; length of outer vertical seta 0.38 mm ± 0.01 (*n* = 16), length of fronto-orbital seta 0.28 mm ± 0.00 (*n* = 16) (see Tables 6, 7).

***Thorax*.** Scutum densely pruinose, humeral calli and lateral sections behind intrascutal suture thinly pruinose, almost glossy; proepisternum with dorsal third blackish, ventral area brown; anepisternum, anepimeron and greater ampulla brown, leading to striking central brown area on pleura (Figs 55, 57); posterodorsal section of katepisternum brown, other pleura blackish; glossy sections on pleura include ventral edge of anepisternum and large central spot on katepisternum (Fig. 57); scutellar spine/scutellum ratio: 0.95 ± 0.01 (*n* = 16, Table 6); scutellar spine/body length ratio: 0.064 ± 0.001 (*n* = 16); apical seta/scutellar spine ratio: 1.14 ± 0.02 (*n* = 12); scutellar length/scutellar width (at base) ratio: 0.66.

***Wing*.** Central brownish spot around crossvein r-m in distal tip of cell br and basal fifth of cell r4+5, somewhat extending into cell bm+dm (Figs 3, 55); some infuscation around vein M4 proximal to crossvein dm-m; vein CuA+CuP from vein CuP onward extending under angle of 30° to wing margin in almost straight line (Fig. 3); cell cua triangular; vein M4 continuing distal of crossvein dm-m, gradually thinning, very slightly turned downwards towards wing margin.

***Legs*.** Femur 1 on almost distal half of inner side with broad brown stripe which apically broadens (Fig. 55); femur 2 with brown stripes on distal third of inner and outer sides, femur 3 with brown stripes on distal quarter of inner and outer sides (Fig. 59); femur 1 (Figs 55, 58) incrassate, l/w ratio: 3.30 ± 0.02 (*n* = 16, Tables 3, 6), femur 1 much less incrassate than in other *Centrioncus* (see Tables 6, 7); two rows of spinous setae (Fig. 58) on distal two-thirds of femur 1 with 6.8 ± 0.2 setae (*n* = 31, Tables 3, 6), inner row with 3.7 ± 0.1 setae, outer row with 3.1 ± 0.0 setae; two rows of tubercles (Fig. 58) on distal three-quarters of femur 1 with 32.7 ± 0.6 tubercles (*n* = 31, Tables 3, 6), inner row with 15.2 ± 0.3 tubercles, outer row with 17.5 ± 0.4 tubercles; femur 3 ventrodistally with 4.9 ± 0.2 tubercles (*n* = 32) in single row (Fig. 59); setal formula: 3.1, 3.7, 17.5, 15.2, 4.9 agrees with formula of type series given by De Meyer (2004): 3, 3–4, 18, 15, 4.

***Preabdomen*.** Tergites blackish brown, thinly pruinose, posterior edges somewhat paler, more pruinose; tergite 2 with small pale spots in posterolateral corners (Figs 55, 60); tergite 3 with pale posterior band (Fig. 60); sternites pale brown; lateral edges of membranous ventral areas with dark lateral spots; sternite 1 rectangular but somewhat constricted mesally (Fig. 72); sternite 2 slightly tapering posteriorly, no intersternite present, only mesal anterior edge of sternite 2 slightly more sclerotised (Fig. 72); sternite 3 rectangular, sternite 4 trapezoidal, broadening posteriorly (Fig. 72); sternite 5 rectangular; sternite 6 short and broad, almost as broad as segment (Fig. 68); sternites 1 and 2 glossy, other sternites pruinose (Fig. 72); first spiracle in membrane.

***Female postabdomen*.** Seventh spiracle at edge of tergite (Fig. 62); anterior sclerite of sternite 7 with w/l ratio: 2.6 (3.1 in De Meyer 2004; fig. 6; see also Table 8), glossy on basal third but pruinose posteriorly (Figs 61, 62); posterior sclerite weakly U-shaped, with posterolateral extensions (Figs 61, 62), almost extending to posterior corners of tergite 7, lateral sections with characteristic outward curve, uniformly pruinose; tergite 8 consisting of two oblong plates, narrowly separated mesally, large central sections more sclerotised (Fig. 65); tergite 10 (Fig. 65) inverted mitre-shaped, extending posteriorly between cerci, clothed in microtrichia and with two pairs of setulae; cercus rather broad, l/w ratio: 2.3 (Fig. 65, Table 8); subanal plate (Figs 63) short, broad, almost triangular with rounded apex, ventrally clothed in microtrichia, with ~ 26 setulae on apical half, shape of subanal plate unusual in the genus (compare Fig. 63 vs. Figs 46, 84, 105, and Feijen (1983: figs 47–65); spermathecae well-sclerotised, smooth, rounded with shallow apical dimple (Fig. 64), junction of ducts of paired spermathecae V-shaped.

***Male postabdomen*.** Epandrium, cerci and surstyli of additional specimens conform to drawings and description by De Meyer (2004) (Figs 68–70); microtrichia on outer side of surstylus shown in Fig. 70; common base of outer and median arm of surstylus long and slender; outer arm with 6–9 tubercles; median arm with six dark, spinous setae apically; inner arm of surstylus two-thirds length of median arm; subepandrial clasper (Figs 69, 70) trapezoidal, basally narrower, mesal apical corner rounded, lateral apical corner distinctly pointed, glabrous, with ~ 11 setulae on inner side; cercus (Fig. 71, Table 8) with broad lateral extension distally, with “foot” slightly concave distally, length/greatest width ratio: 1.7 (Table 8); ejaculatory apodeme + sac (Fig. 73, Table 9) very large, 11.6% of body length, apodeme club-shaped, basally slender.

***Egg.*** Female from Ngangao carried nine developed and developing eggs in abdomen; eggs (Figs 66, 67) ≤ 1.04 mm in length; slightly elevated longitudinal ridges spanning from anterior pole to posterior pole with fine, nearly hexagonal microstructure between ridges (Fig. 67).

#### Distribution and habitat.

The localities of the type series and the specimens now studied are indicated on the map for Eastern Africa (Fig. 29). The distribution appears to be limited to the Taita Hills, a Precambrian mountain range located in the Taita-Taveta County in south-eastern Kenya. These hills consist of three massifs: Dabida (Dawida), Sagalla and Kasigau. *Centrioncusbytebieri* is only known from the Dabida Massif. No *Centrioncus* are known from Sagalla, while *C.copelandi* sp. nov. occurs in Kasigau. The type series was collected in the forest remnants of the Dabida complex in the Ngangao, Chawia and Yale Forests, as well as in the Mbololo Forest. The new specimens also came from Ngangao, Chawia and Mbololo Forests, while as new locations the Fururu and Vuria Forests can now be added. De Meyer (2004) found that his collecting sites were situated from 1400–1800 m, while the specimens now studied were collected from 1680–2162 m. De Meyer added that the species is common and “found on leaves of lower vegetation (shrubs, plants), usually in shaded places in the forests.” The gravid female was collected in the last week of January/first week of February, just before the start of the long rains in Kenya. The male collected in the last week of July/first week of August proved to be teneral, so emerging after the end of the long rains.

#### Remarks.

The additional specimens were largely from the type locality. Male and female genitalia, but also external characters like colour pattern and large size, conform to the description by De Meyer (2004). However, some differences and additions should be indicated. For the pleura, the brown anepimeron and greater ampulla also form part of the large central brown area. The wing has a central wing spot. De Meyer’s description of the posterior sclerite of female sternite 7 was fine but the drawing (fig. 6) shows a rather small sclerite. De Meyer gave a very brief description of the subanal plate. As this plate is very characteristic, it needed redescription. De Meyer described the female cercus as “not so narrow” but gave as l/w ratio: 4.2, which differs from the ratio 2.3 found here. Additional or more detailed illustrations have now been provided for various characters.

### ﻿ Centrioncus copelandi sp. nov.

Taxon classificationAnimaliaDipteraDiopsidae

50EF0FE7-44CA-5020-B028-348CD8E248ED

https://zoobank.org/BC6DFC78-D93E-4A68-B5FE-24EE081A4227

Figs 4 , 29 , 74–79 , 80–83 , 84–87 , 88–91 , 92 , Tables 8 , 9

#### Type material.

Kenya, ***holotype***, ♂, Coastal Prov., Kasigau Mtn, 3.82700°S, 38.64875°E, 1065 m, 28.xii.2011–11.i.2012, R. Copeland (NMKE). ***Paratype***: 1 ♀, Coastal Prov., Kasigau Mtn, 3.82667°S, 38.64982°E, 1117 m, 30.vi–13.vii.2011, R. Copeland (NMKE).

#### Diagnosis.

**Figures 74–79.**
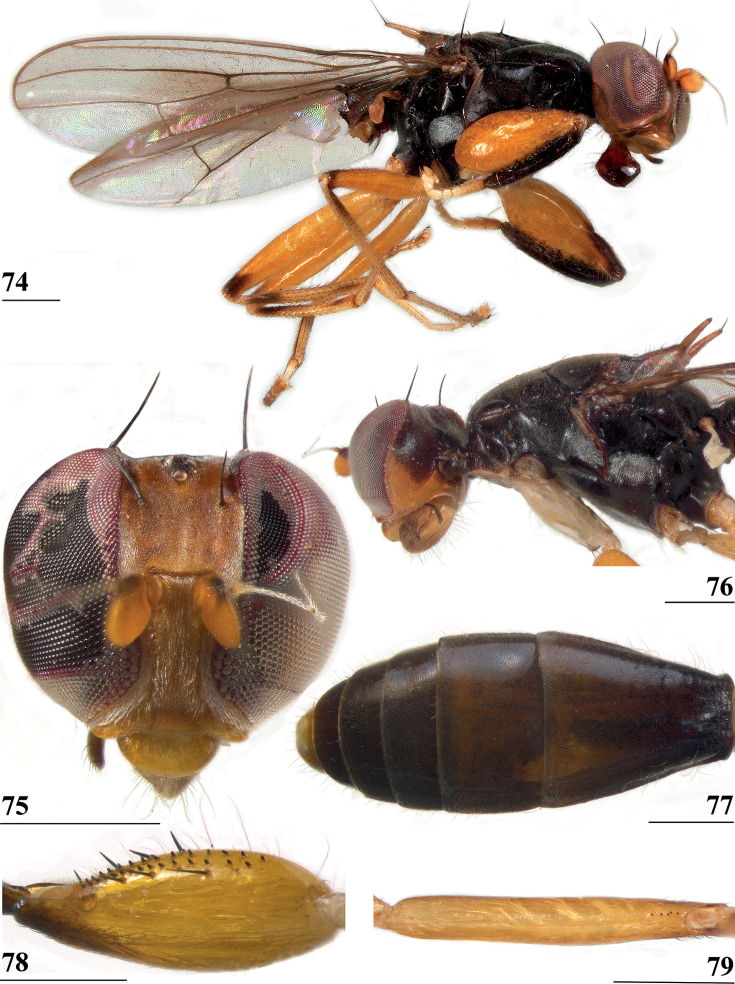
*Centrioncuscopelandi* sp. nov., Kasigau Mtn **74** ♂, holotype, lateral view (abdomen removed) **75–79** ♀, paratype **75** head, frontal view **76** head posterolateral view, thorax, lateral view **77** abdomen, dorsal view **78** fore femur, ventral view **79** hind femur, ventral view. Scale bars: 0.5 mm.

*Centrioncuscopelandi* sp. nov. can be recognised by a mesally slightly depressed, pruinose frons with glossy spots; glossy collar; pruinose blackish brown scutum; dark brown scutellum with brown edges and spines; pleura blackish brown (Figs 74, 76); scutellar spine/scutellum ratio: 0.87–1.00; apical seta/scutellar spine ratio: 0.86–0.92; brown, strongly incrassate fore femur (l/w ratio: ~ 2.65) with ~ 31.7 tubercles, dark brown stripe dorsally on apical third of inner side; very large, distinct central wing spot in distal third of cell br, basal two-fifths of cell r4+5 and distal two-thirds of cell bm+dm (Fig. 4); tergites blackish brown; female 7^th^ spiracle in tergite; sternite 4 rectangular; sternite 5 trapezoidal, invaginated anteriorly; sternites 4 and 5 with pair of strongly sclerotised spots; sternite 6 trapezoidal, 1.5× as broad as sternites 1–5; anterior sclerite of female sternite 7 with w/l ratio: ~ 3.1 (Fig. 81); posterior sclerite of female sternite 7 truncated U-shaped, straight anterior and lateral edges with angular anterolateral corners; female cercus elongate, l/w ratio: ~ 4.4; subanal plate pentagonal, apically tapering into short extension; spermathecae rounded, basally flat (Fig. 85), with small apical dimple; spermathecal ducts with constriction near spermathecae; outer and median arms of surstylus well separated, with short broad common base; outer arm constricted at base, apically broadening to 3× width of base, sides concave, with 16 apical tubercles (Fig. 88); median arm a slender rod, apically some long setulae but no spinous setae, slightly shorter than outer arm; inner arm twice as broad as median arm and slightly longer, apically with shape of cap opener; subepandrial clasper elongate, basal third constricted, apical corners angular, apically strongly convex; cercus (Fig. 89) with broad lateral extension on distal third, apically convex.

#### Description.

***Measurements*.** Body length ♀ 5.1 mm, ♂ 5.0 mm, width of head ♀ 1.08 mm, ♂ 1.13 mm, wing length ♀ 4.3 mm, ♂ 4.3 mm, length of scutellar spine ♀ 0.34 mm, ♂ 0.31 mm.

***Head*.** Frons slightly depressed mesally, brown but mesally and anteriorly paler brown, black stripe posterior to FOS; frons pruinose, anterolaterally more densely pruinose, black stripe behind FOS glossy, small glossy spots lateral to ocellar tubercle (Fig. 75); occiput blackish brown, with lateroventral corners yellowish brown (Fig. 76), uniformly pruinose, more densely pruinose dorsomesally; face yellowish brown, pruinose, denser pruinosity along eye margins; antenna yellowish brown, funiculus with dark spot at base of arista; maxillary palpus dark; OVS 0.31 mm in ♀ and 0.29 in ♂, FOS 0.24 mm in ♀ and ♂.

***Thorax*.** Collar glossy dark brown; scutum blackish brown, pruinose, posterior half of humeral callus glossy (Fig. 76); scutellum dark brown, pruinose, posterior and lateral edges and scutellar spines yellowish brown (Figs 74, 76); pleura dark brown, pleura mainly pruinose, glossy sections include anterior proepisternum, ventral edge of anepisternum, ventral half of katepisternum and ventral spot on anepimeron, densely pruinose spots on posterior section of anepimeron and dorsoposterior section of katepisternum (Figs 74, 76); posterior notopleural, supra-alar and infra-alar setae present, infra-alar seta twice as long as posterior notopleural and supra-alar setae, supra-alar seta on inconspicuous carina (Fig. 74); basiliform prosternum lanceolate and anteriorly sharply pointed and with line-like extension; scutal length/scutal width ratio: 1.1; scutellum projecting at angle of just > 20° from body axis; scutellar spines almost aligned with dorsal plane of scutellum, diverging at angle of ~ 50°; scutellar spine/scutellum ratio: 1.00 in ♀ and 0.87 in ♂; scutellar spine/length of body ratio: 0.067 in ♀ and 0.063 in ♂; apical seta/scutellar spine ratio: 0.86 in ♀ and 0.92 in ♂; scutellar length/scutellar width (at base) ratio: ~ 0.70.

***Wing*.** Almost transparent with very large, distinct, brownish central spot in distal third of cell br, basal two-fifths of cell r4+5, distal two-thirds of cell bm+dm and slightly extending into cells r2+3, m1 and m4 (Fig. 4); glabrous basal areas only include cells bc and c and small basal and subbasal spots in cell br; crossvein h distinct; cell sc closed; vein CuA+CuP from vein CuP onward extending under angle of 25° to wing margin in almost straight line (Fig. 4); cell cua triangular; vein M4 continuing distal of crossvein dm-m in almost straight line to wing margin; alula distinct; crossvein bm-m very vaguely indicated.

***Legs*.** Coxa 1 and trochanter 1 whitish (Fig. 76), thinly pruinose; femur 1 glossy, yellowish brown with dark brown stripe on apical third of dorsal part of inner side (Figs 74, 78); tibia 1 dark brown, pruinose; metatarsus 1 yellowish brown, other tarsal segments whitish; mid and hind legs pale yellowish brown; apex of femur 2 brown; femur 3 dark brown on apical seventh (Fig. 74); tibia 3 with brown spots basally and apically (Fig. 74); femur 1 strongly incrassate in ♀ and ♂ (Figs 74, 78), l/w ratio: 2.62 in ♀ (*n* = 1) and 2.67 in ♂ (*n* = 1), inner row of spinous setae (Fig. 78) with 4–5 setae (*n* = 3), outer row with 4 setae (*n* = 3), inner row of tubercles with 15.3 tubercles (range 15–16, *n* = 3), outer row with 16.3 tubercles (range 15–17, *n* = 3); femur 3 (Fig. 79) with 5.0 tubercles (range 4–6, *n* = 3); setal formula 4.0, 4.7, 16.3, 15.3, 5.0; femora 2 and 3 without apical spurs.

**Figures 80–83.**
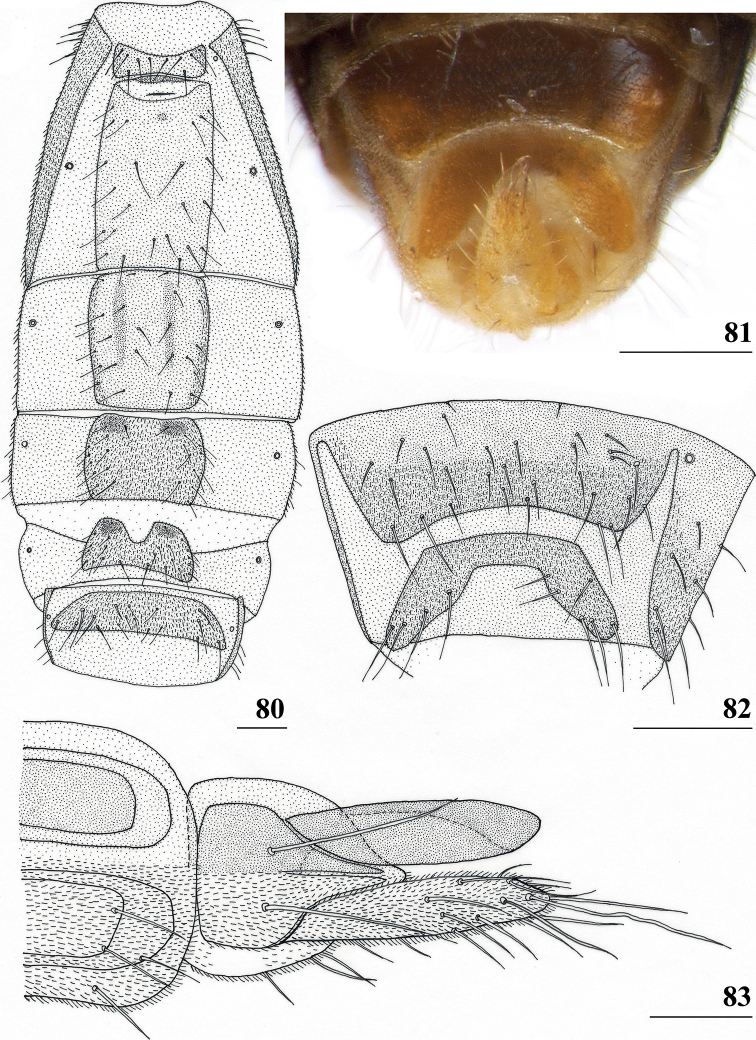
*Centrioncuscopelandi* sp. nov., ♀, paratype, Kasigau Mtn **80** sternites 1–6, ventral view **81, 82** sternite 7, ventral view **83** terga 8, 10 and cerci, dorsal view. Scale bars: 0.2 mm

***Preabdomen*.** Tergites blackish brown (Fig. 77), very thinly pruinose, posterior edges of tergites 2 and 3 with more densely pruinose band; sternites dark brown; lateral edges of membranous ventral areas with dark brown spots; sternite 1 rectangular, constricted mesally (Fig. 80); intersternite 1–2 very dark, laterally acuminate, with thin lateral connections to main sternite 2; sternite 2 rectangular, posterior of intersternite with small membranous area; sternite 3 rectangular with two narrow, elongate, more sclerotised areas (Fig. 80); sternite 4 rectangular with convex lateral sides and two distinct, heavily sclerotised spots anteriorly; sternite 5 trapezoidal, anteriorly strongly constricted mesally, with two distinct, heavily sclerotised spots anteriorly (Fig. 80); sternite 6 trapezoidal, ~ 1.5× as broad as sternites 1–5; sternites 1–3 glossy, sternites 1 and 3 with a few microtrichia laterally; sternites 4–6 pruinose; 1^st^ spiracle in membrane.

**Figures 84–87.**
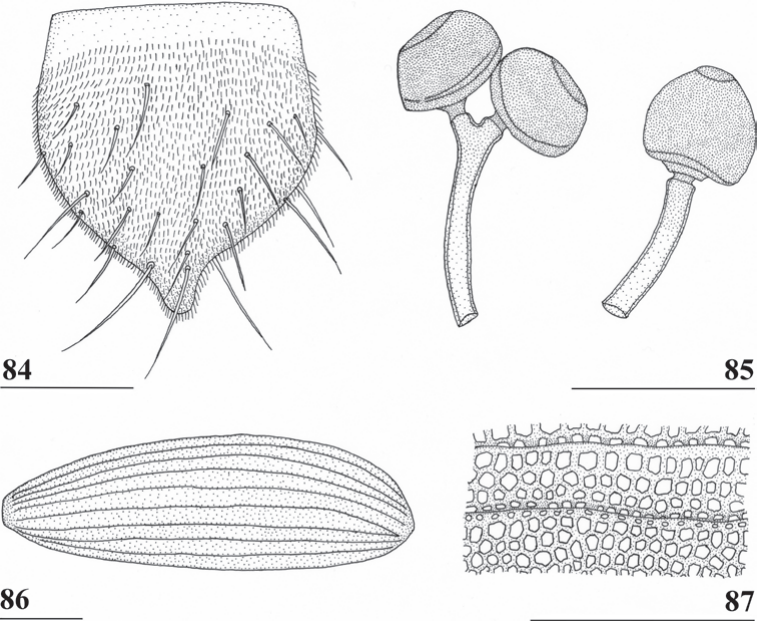
*Centrioncuscopelandi* sp. nov., ♀, paratype, Kasigau Mtn **84** subanal plate, ventral view **85** spermathecae **86** egg 87 egg, detail of external microstructure. Scale bars: 0.1 mm (**84, 85, 87**); 0.2 mm (**86**).

***Female postabdomen*.** Tergite 7 brown, with mesal gap posteriorly, posterior gap extending to lateral sides, lateral edges curved under ventrally; suture in basal ring of segment 7 faint; 7^th^ spiracle well into tergite (Fig. 82); anterior sclerite of sternite 7 rectangular, slightly tapering posteriorly (Figs 81, 82), w/l ratio: ~ 3.1 (Table 8), dark brown, glossy on basal third, pruinose posteriorly (Figs 81, 82), with ~ 30 setulae; posterior sclerite of sternite 7 truncated U-shaped, consisting of broad anterior section and two broad lateral extensions (Figs 81, 82), anterior edge straight and parallel to posterior edge of anterior sternite, lateral edges of extensions straight and diverging posteriorly, anterolateral corners angular, posterolateral apices rounded, clothed in microtrichia and with four setulae at posterior apices; tergite 8 consisting of two oblong plates, well separated mesally (Fig. 83); sternite 8 consisting of two oblong plates; tergite 10 (Fig. 83) onion-shaped, extending posteriorly between cerci, clothed in microtrichia, with one pair of strong setulae; cercus elongate, somewhat tapering posteriorly (Fig. 83, Table 8), l/w ratio: ~ 4.4; subanal plate (Figs 83, 84) somewhat pentagonal, laterally rounded and apically tapering into short extension, clothed in microtrichia except anterior edge, with ~ 26 setulae; spermathecae heavily sclerotised, smooth, rounded, basally flattened (Fig. 85), with small apical dimple; junction of ducts of paired spermathecae V-shaped, spermathecal duct with distinct constrictions near junction with spermathecae (Fig. 85).

**Figures 88–91.**
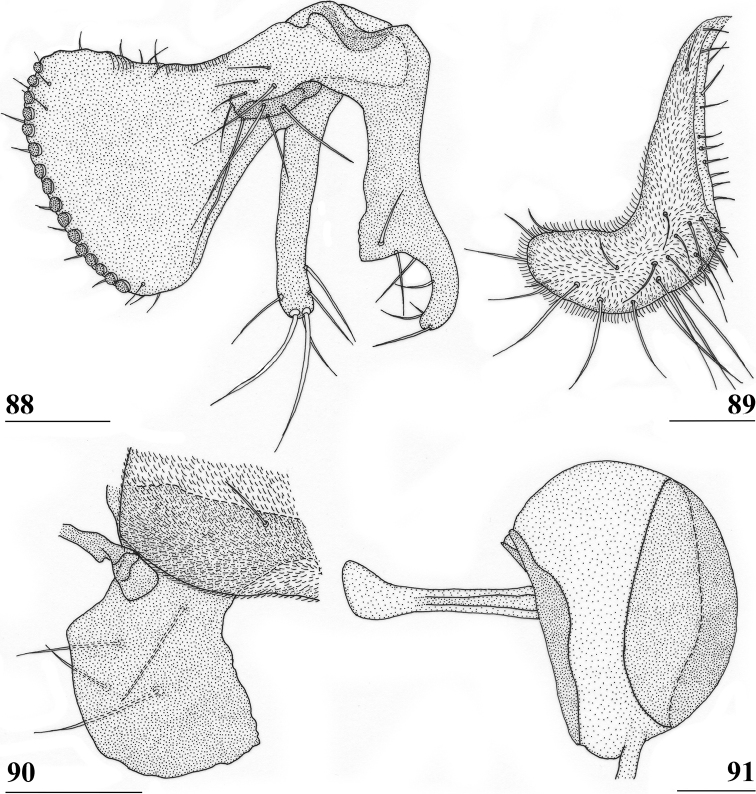
*Centrioncuscopelandi* sp. nov., ♂, holotype, Kasigau Mtn **88** surstylus, inner view **89** cercus, posterior view **90** subepandrial clasper, posterior view **91** ejaculatory apodeme + sac. Scale bars: 0.1 mm.

***Male postabdomen*.** Tergite 6, sternite 8 and epandrium uniformly blackish brown; epandrium broad and rounded (too damaged to measure w/l ratio), clothed in microtrichia; outer and median arms of surstylus well separated, with short broad common base (Fig. 88); outer arm deeply constricted at base with concave lateral sides, broadening apically to ~ 3× basal width, apically with row of 16 tubercles, basally with nine setulae (2 very long) on inner side and a few small setulae along apical edge, on outer side with a few small setulae and central patch of microtrichia; median arm (Fig. 88) slender, parallel-sided, rod-shaped, almost as long as outer arm, apically with four small setulae and two long setulae, no spinous setae present; inner arm twice as broad as median arm and slightly longer, apical quarter abruptly narrowed, cap opener-shaped, apically with seven setulae; subepandrial clasper (Fig. 90) somewhat elongate, basal third constricted, apical corners angular, apically strongly convex, glabrous, with four long setulae on inner side; cercus (Fig. 89, Table 8) slender, with broad lateral extension on distal third, apically convex, clothed in microtrichia and short setulae, 7 longer setulae along apical edge, length/greatest width ratio: 1.5 (Table 8); ejaculatory apodeme + sac (Fig. 91, Table 9) very large, 10.1% of body length, ejaculatory apodeme slender, apically broadening.

***Egg.*** Female with two damaged full-grown eggs and some pieces of undeveloped eggs in abdomen. Eggs (Fig. 86) measured 1.01 mm in length with elevated longitudinal ridges spanning from anterior pole to posterior pole with fine, roughly hexagonal microstructures between ridges (Fig. 87).

#### Distribution and habitat.

**Figure 92.**
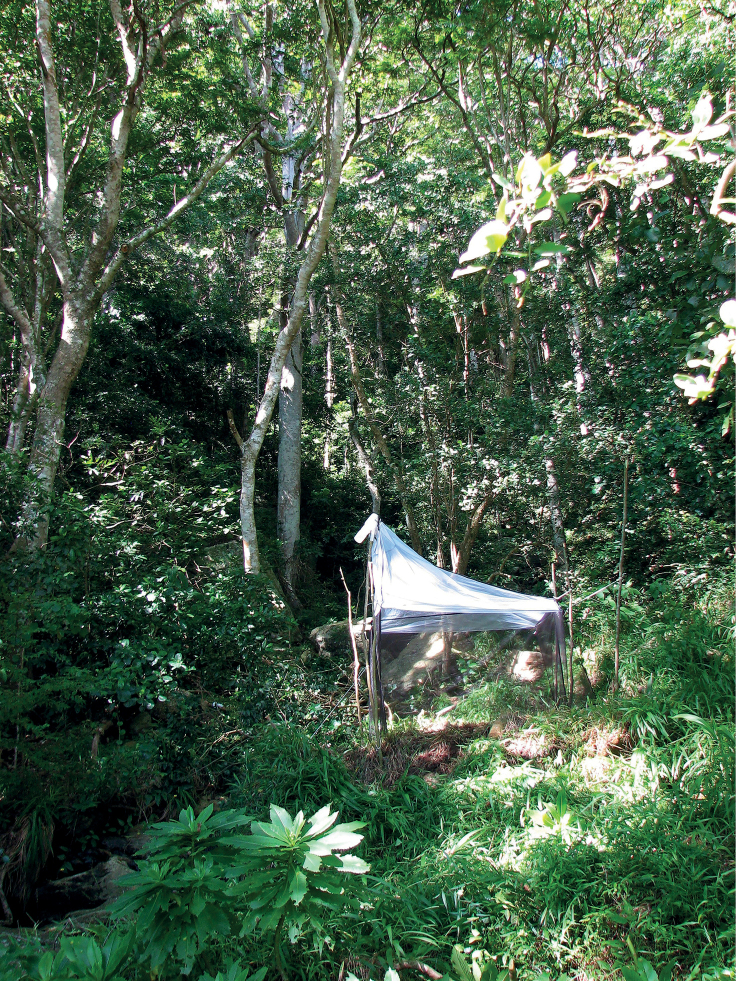
*Centrioncuscopelandi* sp. nov., Kenya, Kasigau Mtn, collection site at 1117 m. Photograph by Robert Copeland.

The localities of the type series are indicated on the map for Eastern Africa (Fig. 29). The collecting sites varied from 1056 to 1117 m in altitude. The habitat of the Kasigau site at 1117 m is shown in Fig. 92. The distribution appears to be limited to the Kasigau Massif of the Taita Hills (see also under *C.bytebieri*). *Centrioncusbytebieri* is only known from the Dabida Massif of the Taita hills. The gravid female was collected in early July, in the middle of the dry season which is unusual for *Centrioncus* flies.

#### Etymology.

The specific epithet of *C.copelandi* sp. nov. refers to the name of its collector Dr Robert S. Copeland (ICIPE), who contributed a significant number of *Centrioncus* specimens to this study.

#### Remarks.

From the shape of the outer arm of the surstylus (apically much wider than at base, with large number of 16 or 17 tubercles), absence of spinous setae on the median arm of the surstylus, the very large central wing spot and anteriorly strongly constricted sternite 5, it can be postulated that the closest known relative of *C.copelandi* sp. nov. is *C.prodiopsis*. From a geographical point of view that also makes some sense (Fig. 29), although *C.bytebieri* occurs closer to *C.copelandi* sp. nov.

### ﻿ Centrioncus crassifemur sp. nov.

Taxon classificationAnimaliaDipteraDiopsidae

65C3CDB4-A6A2-5E56-990C-4FE87300FE33

https://zoobank.org/C2531274-139D-4B06-8662-F44C89C8D602

Figs 5 , 93–96 , 97–100 , 101–105 , 106–109 , Table 8

#### Type material.

Angola, ***holotype***, ♀, Salazar I.I.A.A. N’dalatando (Salazar) [most probably Instituto de Investigação Agronomica de Angola at Quilombo, 5 km to north of N’dalatando, 8°53'1"S, 14°44'59"E, 600– > 1000 m], 9–15.iii.1972, Southern African Exp. B.M. 1972, no collector given [probably leg. P.M. Hammond] (NHMUK).

#### Diagnosis.

**Figures 93–96.**
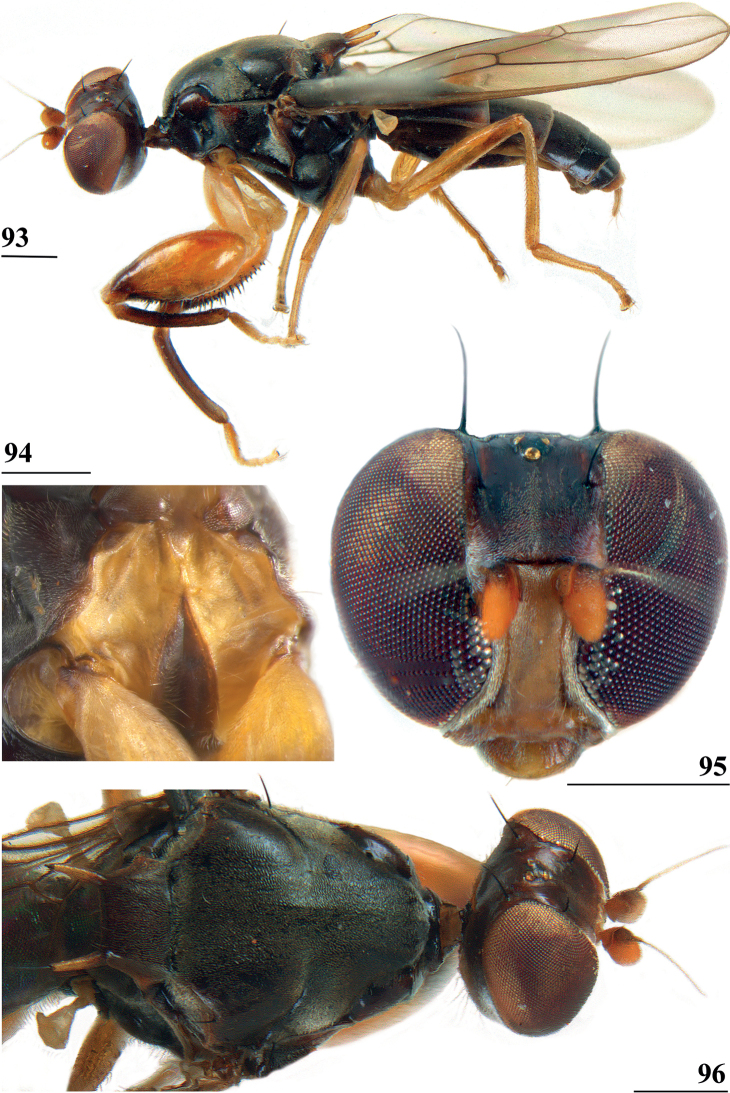
*Centrioncuscrassifemur* sp. nov., ♀, holotype, N’dalatando **93** habitus, lateral view **94** basiliform prosternum, ventral view **95** head, frontal view **96** head, thorax, dorsal view. Scale bars: 0.5 mm (**93, 95, 96**); 0.2 mm (**94**).

*Centrioncuscrassifemur* sp. nov. can be recognised by the mesally slightly depressed, pruinose frons; mainly pruinose collar; pruinose blackish brown scutum with brownish humeral calli; dark brown, pruinose scutellum with pale scutellar spines; pleura blackish brown with anterodorsal corner of anepisternum brown (Fig. 93); scutellar spine/scutellum ratio: 0.81; apical seta/scutellar spine ratio: 0.81; fore femur (Figs 93, 98) strongly incrassate (l/w ratio: 2.36) with ~ 41.5 tubercles, pale brown, on distal third dark brown on both sides; distinct central brownish wing spot around crossvein r-m in distal tip of cell br and basal fifth of cell r4+5, somewhat extending into cell bm+dm (Fig. 5); tergites dark brown, posterolateral corners of tergites 2–4 paler brown; female 7^th^ spiracle just in tergite; sternite 4 rectangular; sternite 5 square; sternite 6 trapezoidal, 1.5× as wide as sternites 1–5; anterior sclerite of female sternite 7 broad, very short (w/l ratio: ~ 8.6); posterior sclerite of female sternite 7 large, membranous, trapezoidal plate, two tiny more sclerotised sections in anterolateral corners (Figs 102, 104); female cercus elongate, l/w ratio: 4.3; subanal plate (Fig. 105) laterally rounded, pentagonal, apically pointed; spermathecae round in cross section, flattened with large apical dimple and central pinnacle (Figs 106, 107).

#### Description.

***Measurements*.** Body length ♀ 5.1 mm, width of head ♀ 1.23 mm, wing length ♀ 4.5 mm, length of scutellar spine ♀ 0.31 mm.

***Head*.** Frons slightly depressed mesally, dark brown, with lateral margins pale brown, mainly pruinose except glossy areas lateral to ocellar tubercle (Figs 95, 96); occiput glossy brown with some pruinescence posteriorly to ocellar tubercle and densely pruinose margins near eyes; face yellowish brown, thinly pruinose, with denser pruinosity along eye margins (Fig. 95); antenna yellowish brown, dorsal section of funiculus and basal segments of arista darker (Figs 95, 96); maxillary palpus dark; outer vertical seta 0.30 mm, fronto-orbital seta 0.18 mm.

***Thorax*.** Collar dark brown, mainly pruinose, mesally and anteriorly glossy (Fig. 96); scutum blackish brown and pruinose, humeral callus brown and more glossy (Figs 93, 96); scutellum dark brown, pruinose; scutellar spines pale, but narrowly brownish basally (Figs 93, 96); pleura dark brown with only anterodorsal corner of anepisternum paler brown; pleura mainly thinly pruinose, densely pruinose areas around and below anterior spiracle, anterior and posterior sections of anepisternum, and dorsal anterior and posterior sections of katepisternum, glossy sections include ventral edge of anepisternum and ventral half of katepisternum (Fig. 93); posterior notopleural seta, supra-alar and infra-alar setae present, supra-alar seta ca. two-thirds length of other two setae; basiliform prosternum lanceolate and sharply pointed anteriorly (Fig. 94); scutal length/scutal width ratio: 1.22 (Fig. 96); scutellum projecting at angle of ~ 20° from body axis; scutellar spines almost aligned with dorsal plane of scutellum (Fig. 93), diverging at angle of just > 30°; scutellar spine/scutellum ratio: 0.81; scutellar spine/length of body ratio: 0.06; apical seta/scutellar spine ratio: 0.81; scutellar length/scutellar width (at base) ratio: 0.74.

***Wing*.** Almost transparent but slightly tinged; distinct central brownish spot around crossvein r-m in distal tip of cell br and basal fifth of cell r4+5, somewhat extending into cell bm+dm (Fig. 5); some infuscation around vein M4 proximal to crossvein dm-m; glabrous basal areas only include cells bc and c and basal third of cell br; crossvein h distinct; cell sc closed; vein CuA+CuP from vein CuP onward extending under angle of 25° to wing margin in almost straight line; vein M4 continuing distal of crossvein dm-m in almost straight line towards wing margin; cell cua subtriangular; alula distinct; crossvein bm-m vaguely indicated.

**Figures 97–100.**
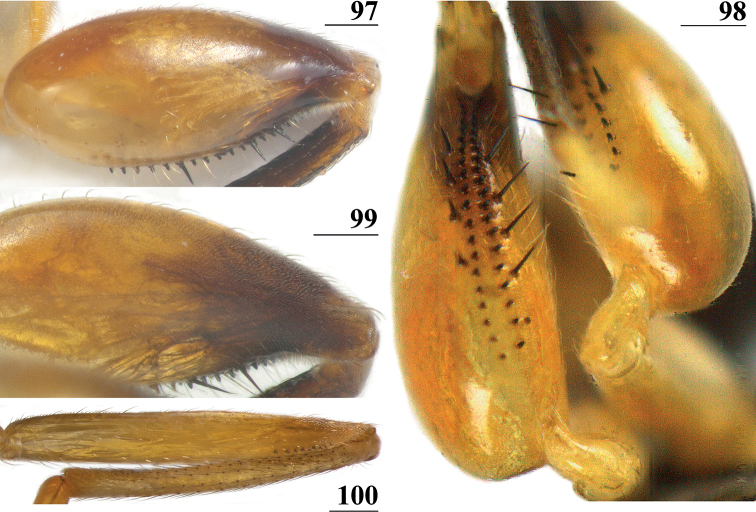
*Centrioncuscrassifemur* sp. nov., ♀, holotype, N’dalatando **97** fore femur, outer view **98** fore femora, ventral view **99** fore femur, inner view **100** hind femur, inner view. Scale bars: 0.2 mm.

***Legs*.** Fore coxa and trochanter yellowish white, thinly pruinose; fore femur glossy, pale brown with darker brown apex (Figs 93, 97–99); fore tibia glossy brown with paler, more pruinose base; fore metatarsus yellowish brown, other tarsal segments whitish (Fig. 93); mid and hind legs pale yellowish brown, apical quarter of hind femur brown (Fig. 100); fore femur strongly incrassate in ♀ (Figs 97–99), l/w ratio: 2.36 (*n* = 1), fore femur subbasally connected to trochanter (Fig. 98) on inner side [assumed adaptation to very incrassate femora], inner row of spinous setae (Fig. 98) with five setae, outer row with four setae, inner row of tubercles with 20–21 tubercles, outer row with 21 tubercles; hind femora with six and nine (Fig. 100) tubercles; setal formula 4.0, 5.0, 21.0, 20.5, 7.5; no apical spurs on mid and hind femora.

**Figures 101–105.**
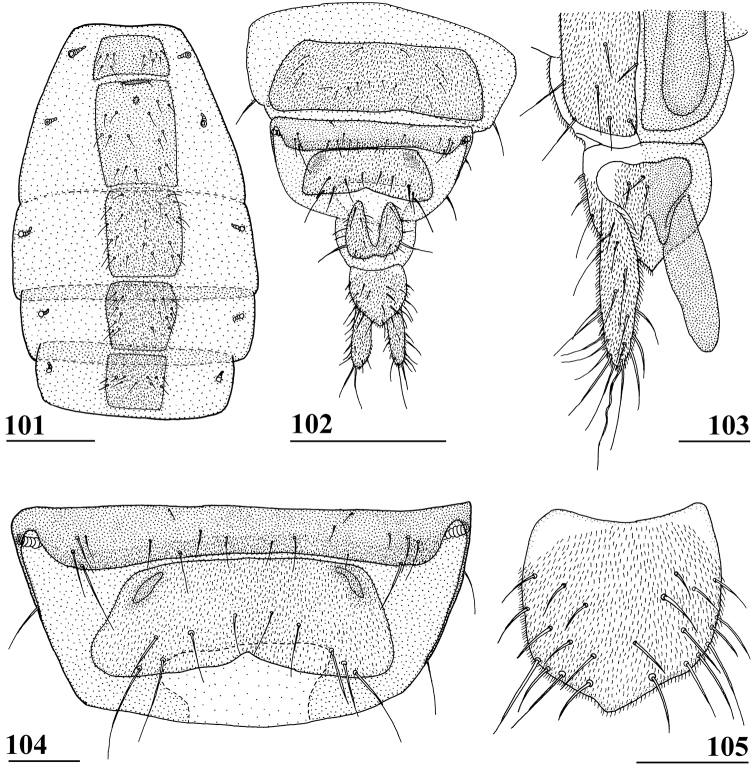
*Centrioncuscrassifemur* sp. nov., ♀, holotype, N’dalatando **101** sternites 1–5, ventral view **102** sternites 6–8, subanal plate, cerci, ventral view **103** tergites 8, 10, cerci, dorsal view **104** sternite 7, ventral view **105** subanal plate, ventral view. Scale bars: 0.5 mm (**101, 102**); 0.1 mm (**103–105**).

***Preabdomen*.** Dark brown, thinly pruinose, posterolateral corners of tergites 2–4 paler brown, posterior half of tergite 7 pale brown (Fig. 93); sternites 1–6 brown, sternites 1 and 2 glossy, sternites 3–6 pruinose; sternite 1 slightly trapezoidal (Fig. 101); sternite 2 anteriorly with very slender, more sclerotised section mesally with two minute setulae laterally; sternites 2–4 all rectangular, slender, more or less equal in width; sternite 5 square; sternite 6 (Fig. 102) trapezoidal, ~ 1.5× as wide as sternites 1–5; 1^st^ spiracle in membrane.

**Figures 106–109.**
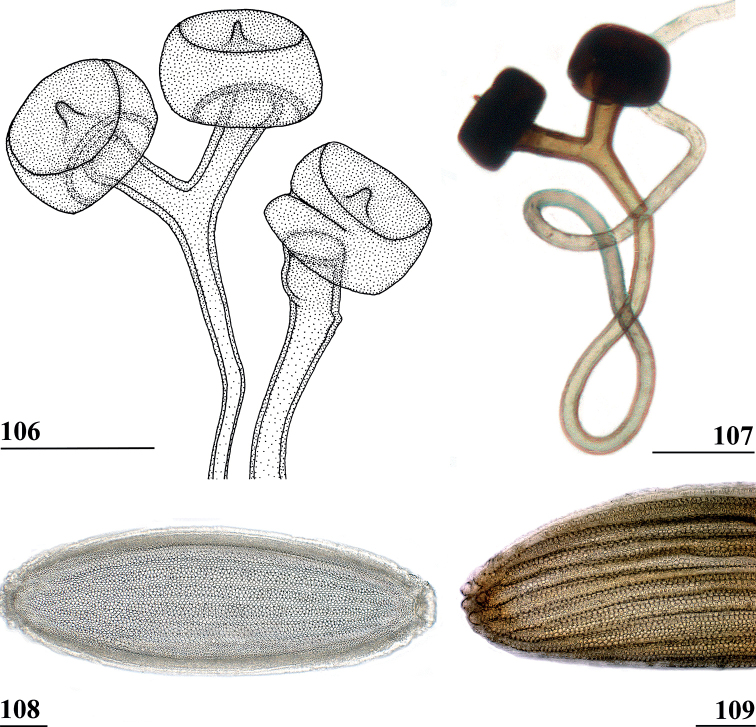
*Centrioncuscrassifemur* sp. nov., ♀, holotype, N’dalatando **106, 107** spermathecae **108** egg (transmitted light) **109** egg, detail (reflected light). Scale bars: 0.1 mm.

***Female postabdomen*.** Tergite 7 (Fig. 104) with large mesal gap posteriorly, lateral edges only marginally curved under ventrally; suture in basal ring of segment 7 hardly visible; 7^th^ spiracle at edge of tergite (Fig. 104); anterior sclerite of sternite 7 rectangular, broad and very short, w/l ratio: ~ 8.6, covered with microtrichia and some setulae on posterior half (Fig. 104, Table 8); posterior sclerite of sternite 7 trapezoidal plate, weakly sclerotised with two very small more sclerotised sections in anterolateral corners (Fig. 104), covered with microtrichia and with ~ 10 setulae posteriorly; tergite 8 consisting of two oblong plates, narrowly separated mesally, central sections more sclerotised (Fig. 103); sternite 8 consisting of two oblong plates, expanding posteriorly, and posteriorly linked mesally (Fig. 102); tergite 10 (Fig. 103) somewhat onion-shaped, extending posteriorly between cerci, laterally bare, otherwise clothed in microtrichia and with two pairs of setulae; cercus elongate, l/w ratio: 4.3 (Table 8); subanal plate (Figs 102, 105) laterally rounded, apically pointed, subpentagonal, ventrally covered with microtrichia and > 20 setulae on apical half; spermathecae heavily sclerotised, smooth, round in cross section, flattened with large apical dimple and peculiar central pinnacle (Figs 106, 107); junction of ducts of paired spermathecae V-shaped (Figs 106, 107).

***Egg.*** Holotype with six almost fully developed eggs in abdomen. Eggs (Figs 108, 109) measured 0.9 mm in length, with longitudinal ridges spanning from anterior pole to posterior pole with fine, nearly hexagonal, microstructures on and in between ridges. With different illumination settings, either ridges become more distinct (Fig. 109) or hexagonal microstructures (Fig. 108).

#### Distribution and habitat.

The single known specimen is from north-western Angola. The holotype was most likely collected in rain forest around the experimental station of the Instituto de Investigação Agronomica de Angola at Quilombo. The altitude of the collecting locality must then have been between 600 and slightly > 1000 m. The gravid holotype was collected in early March, which falls in the second half of the rainy season in Angola.

#### Etymology.

The specific epithet of *C.crassifemur* sp. nov. refers to the remarkably incrassate fore femora.

#### Remarks.

The more sclerotised anterior margin of sternite 2 forms an integral part of sternite 2 (Fig. 101) and is not, like in other *Centrioncus*, with thin lateral connections tied to the main region of sternite 2. Given the shape of the posterior sclerites of female sternite 7, the closest known relative of *C.crassifemur* sp. nov. could be *C.aberrans*. However, it is possible that the shape of sternite 7 simply represents a symplesiomorphic condition. *Centrioncusaberrans* and *C.bururiensis* sp. nov. are geographically speaking its closest known relatives.

### ﻿ Centrioncus decellei

Taxon classificationAnimaliaDipteraDiopsidae

Feijen

F9B5D85A-8243-5D5C-A4D1-59144E3A9F01

**Figures 110, 111.**
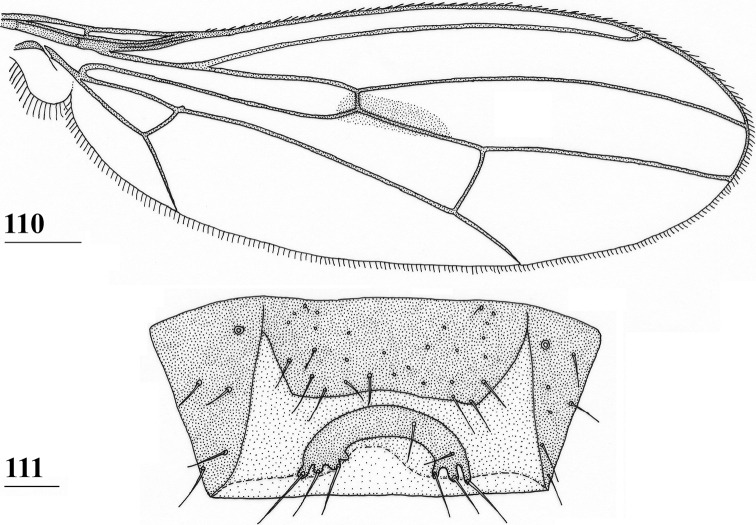
*Centrioncusdecellei*, ♀, holotype, Amanikro, Ivory Coast **110** wing, dorsal view **111** sternite 7, ventral view. Scale bars: 0.5 mm (**110**); 0.1 mm (**111**). Both drawings were processed, based on original pencil drawings from Feijen (1983: figs 12, 26).

Figs 110 , 111 , Table 8

Centrioncus decellei Feijen, 1983: 73.

#### Type material.

Ivory Coast, ***holotype*** ♀, Amanikro, 50 km nw of Abengourou [7°1'0"N, 3°52'0"W, 52 km NW of Abengourou, 194 m], ix. 1961, J. Decelle (MRAC) (not examined, but notes and pencil drawings for Feijen (1983) were used).

#### Diagnosis

**(after Feijen 1983).***Centrioncusdecellei* can be recognised by the flat, uniformly pruinose frons; pruinose blackish brown scutum, brownish humeral calli, chestnut-brown lateral edges; scutellum blackish brown with brown edges and lateral sides, scutellar spines yellowish; blackish brown pleura, brown anterior part of anepisternum and posterior part of anepimeron, katepisternum with brown spot anteriorly; scutellum projecting at angle of 45° from body axis; pale brown fore femur on distal half of inner side with dark brown, stripe-like spot, with ~ 38 tubercles; small, elongate, very vague, brownish central spot around crossvein r-m (Fig. 110), in tip of cell br, mainly in posterior part of basal quarter of cell r4+5, slightly extending into cell bm+dm; vein M4 distally of crossvein dm-m curving downward towards wing margin; tergites blackish brown, apical edges paler; female 7^th^ spiracle in tergite (Fig. 111); female tergite 7 with small mesal gap at posterior edge; female tergite 10 with 3 pairs of setulae; trapezoidal anterior sclerite of female sternite 7 narrow (w/l ratio: ~ 2.7); posterior sclerite of female sternite 7 rounded U-shaped, relatively short lateral arms with tri-lobed posterior apices (Fig. 111); female cercus elongate, l/w ratio: 4.1; subanal plate triangular, with convex lateral sides; spermathecae wrinkled, with a dimple and some tiny tubercles.

#### Supplementary description.

***Wing*.** Small, elongate, very vague, brownish central spot around crossvein r-m (Fig. 110), in tip of cell br, mainly in posterior part of basal quarter of cell r4+5 to three-quarters distance to crossvein dm-m, slightly extending into cell bm+dm; vein CuA+CuP from vein CuP onward extending under angle of 25° to wing margin in straight line; vein M4, especially distal of crossvein dm-m, curving downward to wing margin (Fig. 110).

***Female postabdomen*.** Anterior sclerite of sternite 7 trapezoidal with w/l ratio: ~ 2.7 (Fig. 111, Table 8); posterior sclerite of sternite 7 rounded U-shaped, with relatively short lateral arms, with 3–4-lobed posterior apices (Fig. 111); female cercus elongate with l/w ratio: 4.1 (Table 8); subanal plate triangular with convex lateral sides.

#### Distribution and habitat.

There are several Amanikro locations in Ivory Coast, three of which are north-west of Abengourou. However, given the label distance of 50 km from Abengourou, the correct collecting location must be Amanikro at 7°1'0"N, 3°52'0"W, 52 km NW of Abengourou, 194 m. The holotype was collected in lowland forest. This species is the only *Centrioncus* known to occur at a low altitude, while the single specimen is also the only *Centrioncus* known from West Africa.

### ﻿ Centrioncus decoronotus

Taxon classificationAnimaliaDipteraDiopsidae

Feijen

EB138283-FD5F-5201-99FD-83475C08EE8D

Figs 6 , 29 , 112–116 , 117 , 118–121 , 122–125 , 153 Tables 4 , 7 , 8 , 9

Centrioncus decoronotus Feijen, 1983: 76; McAlpine 1997: 175, 2011: 150; De Meyer 2004: 28; Marshall et al. 2009: 6; Lonsdale 2020: 6, figs 153–159, 405, 423, 425; Feijen and Feijen 2021, figs 2, 29.
Centrioncus prodiopsis : Steyskal 1970: 326 (in part, male description and figs 3, 4).

#### Type material.

Kenya, ***holotype*** ♂, Naivasha [0°41'40"S, 36°22'47"E], vii.1937, H.J.A. Turner (NHMUK). ***Paratypes***: 1 ♀, 1 ♂, same data as holotype; 1 ♀, 1 ♂, Thomson’s Falls [now Nyahururu Falls, 0°2'38.98"N, 36°22'13.04"E], x.1934, F.W. Edwards (NHMUK); 1 ♂, Chania Falls, Aberdare range [0°27'16.70"S, 36°43'1.28"E], x.1934, F.W. Edwards (NHMUK); 1 ♀, Ngong [1°21'22"S, 36°40'8"E], ix.1940, G. van Someren (NHMUK).

#### Material studied.

Kenya: 27 ♀, 25 ♂, Naro Moru River, [0°9'22.93"S, 37°0'50.45"E, 1950 m], 20.vii.1987, H.R. Feijen (RMNH); 6 ♀, 6 ♂, Limuru nr Mrs Mitchels tea plantation, Rain forest, Kiambethu Tea Plantation, [1°7'3.18"S, 36°40'58.11"E, 2167 m], 3.ix.1986, Cobi Feijen (RMNH); 2 ♀, 2 ♂, nr Namanga Tanzania [but in Kenya], Ol Doinyo Orok forest, [~ 2°31'S, 36°47'E, ~ 1800 m], 0.iii.1986, J. Muhangani (RMNH); 1 ♀, 1 ♂, Thomson’s falls, Nyahururu, [0°2'38.98"N, 36°22'13.04"E, 2329 m], 20.iii.1988, H.R. Feijen (RMNH); 3 ♀, 3 ♂, Nyahuru(ru), Upper Imenti Forest, Meru Distr. E. Kenya, [0°3'0"N, 37°31'59"E], vii.1973, E. Balyetagara (CNC); 2 ♀, 3 ♂, Laikipia Co., Thomson’s Falls env. (= Ngare Naro forest), 0.047°N, 36.377°E, 25.xi.2012, D. Gavryushin (ZMUM); 3 ♀, 1 ♂, Laikipia Co., Thomson’s Falls env., 0.047°N, 36.377°E, 21–23.xii.2013, N. Vikhrev (ZMUM); many (photograph), Ngare Naro forest (near Thomson’s Falls), 27.xi.2012, D. Gavryushin; 2 ♀, 2 ♂, Central Province, Katura Forest, Nairobi, shaded mixed upland indigenous forest, 1°14.504'S, 36°49.452'E, 1720 m, 23.iv.2011, A.H. & M.K. Kirk-Spriggs (BMSA); 3 ♀, 1 ♂, Eastern Prov., Nyambene Hills, Itieni forest, at bottom, malaise trap, edge indigenous forest, 0.24433°N, 37.87016°E, 2142 m, 1 ♀ 1 ♂ 2–16.x.2011, 1 ♀ 18.ix–2.x.2011, 1 ♀ 15–29.xi.2011, R. Copeland (ICIPE); 1 ♀, Central Prov., Ngong road forest, Nairobi, fogging, 1°18'57"S, 36°44'27"E, ~ 1830 m, 12–14.ii.1999, T. Wagner (ICIPE); 2 ♀, 3 ♂, Eastern Prov., Njuki-Ini Forest, near forest station, sweep net, edge indigenous forest, 0.51660°S, 37.41843°E, 1455 m, 13.ii.2011, R. Copeland (ICIPE); 1 ♀, 2 ♂, Gatama(i)yu forest, near fishing camp, malaise trap, 0°58.68'S, 36°41.62'E, ~ 2246 m, 20–27.iii.1999, R. Copeland (ICIPE). In total 53 ♀and 49 ♂ were examined.

#### Diagnosis.

**Figures 112–116.**
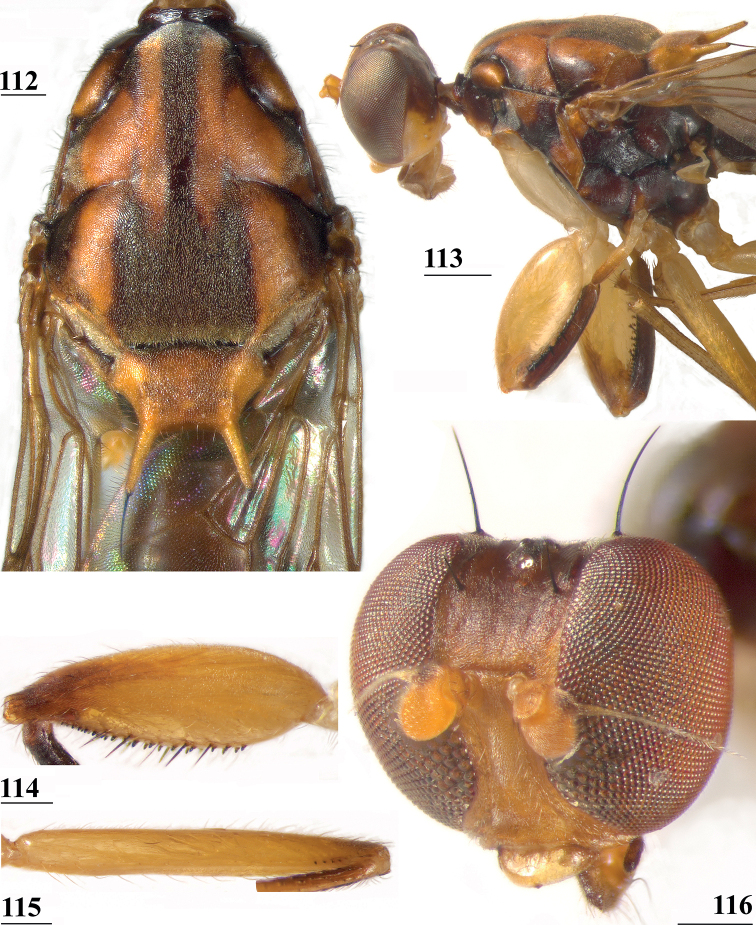
*Centrioncusdecoronotus***112, 113** ♀, Thomson’s Falls **114, 115** ♂, Itieni Forest **116** ♂, Katura Forest **112** thorax, dorsal view **113** head, thorax, lateral view **114** fore femur, inner view **115** hind femur, inner view **116** head, frontolateral view. Scale bars: 0.2 mm (**112, 114–116**); 0.5 mm (**113**).

**Figure 117.**
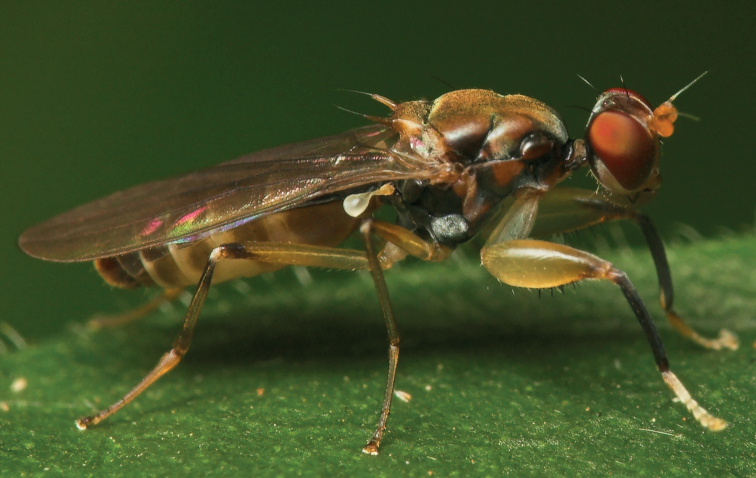
*Centrioncusdecoronotus*, ♂, Nyahururu, Ngare Naro Forest. Photograph by D. Gavryushin.

**Figures 118–121.**
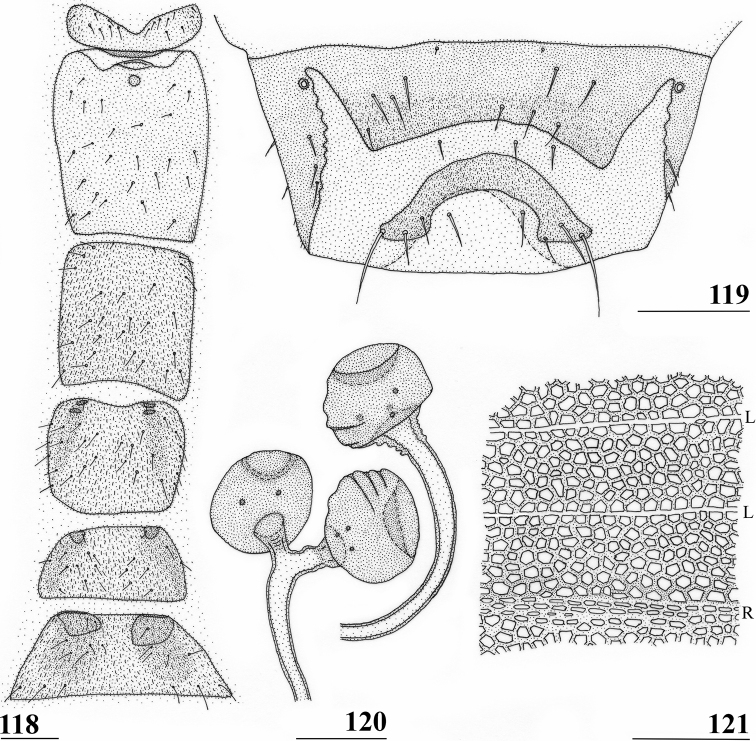
*Centrioncusdecoronotus*, ♀, Ol Doinyo Orok **118** sternites 1–5, ventral view **119** sternite 7, ventral view **120** spermathecae **121** egg, detail of external structure (L = line, R = ridge). Scale bars: 0.2 mm (**118, 119**); 0.05 mm (**120, 121**).

**Figures 122–125.**
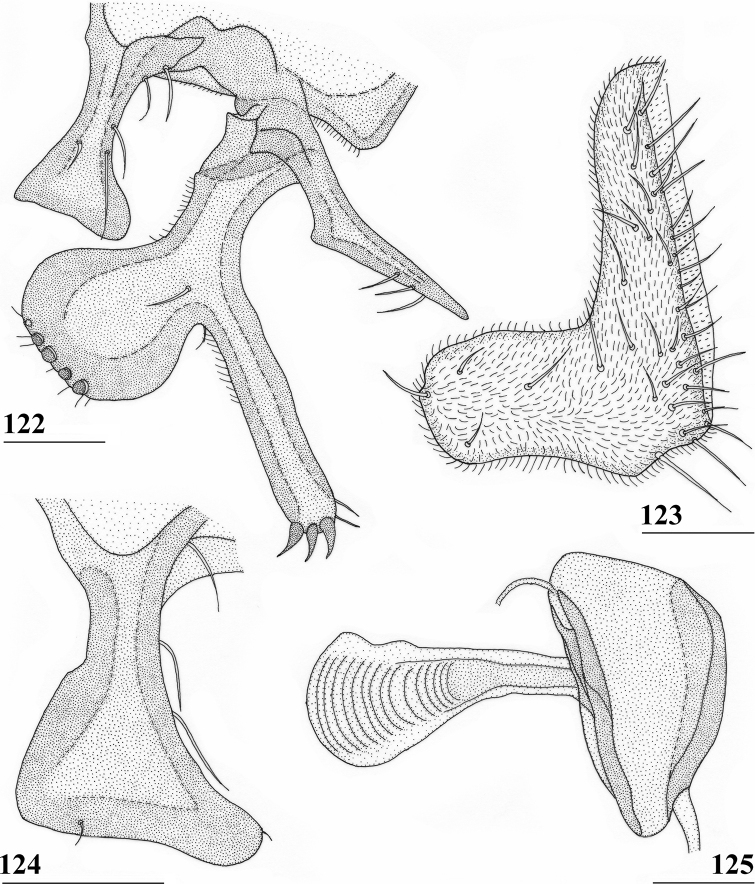
*Centrioncusdecoronotus*, ♂, Ol Doinyo Orok **122** left subepandrial clasper and surstylus, inner view **123** cercus, posterior view **124** right subepandrial clasper, posterior view **125** ejaculatory apodeme + sac. Scale bars: 0.1 mm (**122–124**); 0.2 mm (**125**).

*Centrioncusdecoronotus* can be recognised by the mesally depressed, pruinose frons with glossy spots; glossy collar; scutum with configuration of blackish brown and brown (Figs 112, 113, 117), basic colour chestnut brown (including humeral calli), blackish sutures around humeral calli, blackish mesal stripe on anterior half, square, mesal, blackish area posteriorly of intrascutal sutures; scutellum brown, dorsally darker, scutellar spines brown; pleura blackish brown (Figs 113, 117) with chestnut brown anterodorsal anepisternum, greater ampulla and posterior anepimeron; scutellar spine/scutellum ratio: 0.90; apical seta/scutellar spine ratio: 0.91; yellowish brown, strongly incrassate fore femur (l/w ratio: 2.89) with ~ 34.5 tubercles; brown stripe dorsally on distal half of inner side (Figs 113, 114); large central wing spot (Fig. 6), mainly in basal section of cell r4+5 till just apically of crossvein dm-m, extending into cell br, cell r2+3 and cell bm+dm; tergites dark brown, thinly pruinose, posterolateral corners whitish pruinose; sternite 4 rectangular, laterally two more sclerotised sections, anteriorly 2 pairs of tiny heavily sclerotised spots (Fig. 118); sternite 5 broadening posteriorly, laterally more sclerotised, 1 pair of strongly sclerotised spots anteriorly; sternite 6 trapezoidal, posteriorly 1.6× as wide as sternites 1–4, two more sclerotised plates anteriorly; female tergite 7 with serrated lateral edges; female 7^th^ spiracle in tergite; anterior sclerite of female sternite 7 short (Table 8), w/l ratio: ~ 3.9 (Fig. 119); posterior sclerite of female sternite 7 U-shaped, posterior apices sometimes slightly broadening (Fig. 119); female cercus rather elongate with l/w ratio: 3.6; subanal plate pentagonal, laterally convex, apex acuminate; spermathecae round, somewhat wrinkled, distinct dimple, some tiny tubercles (Fig. 120); common base of outer and median arm of surstylus long, slender; outer arm rounded, deeply constricted near median arm, largest width 1.8× width at base, 4–6 tubercles apically (Fig. 122); median arm slender, rod-shaped, much longer than outer arm, three or four spinous setae apically; inner arm apically acuminate, shorter and narrower than median arm, no apophysis; subepandrial clasper triangular, basal two-thirds narrow, medial apical corner angular, lateral corner extended (Figs 122, 124); cercus (Fig. 123) with broad lateral extension on distal third, apically slightly concave.

#### Supplementary description.

The biometrical data are presented for the series now studied, and compared to the type series. Additional morphological data as well as a few rectifications are presented. Various aspects of the morphology are now illustrated by photographs, while line drawings are presented for flies from Ol Doinyo Orok, a location distant from the type location.

***Measurements*.** For type series of 3 ♀ and 4 ♂, Feijen (1983) provided the following measurements: body length ♀ 5.3 mm and ♂ 5.2 mm, head width ♀ 1.23 mm and ♂ 1.17 mm, wing length ♀ 5.0 mm and ♂ 4.7 mm, scutellar spine length ♀ 0.36 mm and ♂ 0.31 mm. For the much larger series now available, 22 ♀ and 22 ♂ were measured in more detail. In Table 4, measurements and other quantitative characters are presented for this series. In this table, data are presented for females and males separately. The table shows that differences between females and males for quantitative characters are marginal. The body length and various other measurements are slightly larger in the females. In Tables 6, 7 the data for females and males are combined, so that large series can be compared with the other *Centrioncus* species for which large numbers were available. The original measurements of the type series fall well within the ranges provided here: body length 5.15 mm ± 0.04 (range 4.64–5.61, *n* = 44), head width 1.14 mm ± 0.01 (range 1.02–1.24, *n* = 44), wing length 4.51 mm ± 0.03 (range 4.09–5.00, *n* = 39), scutellar spine length 0.33 mm ± 0.00 (range 0.29–0.37, *n* = 44).

**Table 4.** Quantitative characters for *Centrioncusdecoronotus*. Given are mean ± standard error, range and number of records for females and males. Measurements in mm.

Character	♀			♂		
	x̄ ± SE	range	*n*	x̄ ± SE	range	*n*
head width	1.14 ± 0.01	1.04–1.23	22	1.15 ± 0.01	1.02–1.24	22
body length	5.27 ± 0.02	5.06–5.55	22	5.03 ± 0.06	4.64–5.61	22
wing length	4.60 ± 0.04	4.33–5.00	18	4.43 ± 0.04	4.09–4.76	21
sc. sp. length	0.34 ± 0.00	0.30–0.37	22	0.32 ± 0.00	0.29–0.36	22
apical seta length	0.31 ± 0.01	0.29–0.36	10	0.28 ± 0.01	0.24–0.34	11
scutellum length	0.37 ± 0.00	0.34–0.41	22	0.36 ± 0.00	0.31–0.40	22
head w./body l.	0.22 ± 0.00	0.20–0.23	22	0.23 ± 0.00	0.21–0.25	22
sc. sp. l./body l.	0.064 ± 0.001	0.057–0.072	22	0.063 ± 0.001	0.056–0.068	22
sc. sp. l./sc. length	0.90 ± 0.01	0.76–1.00	22	0.89 ± 0.01	0.80–0.96	22
ap. seta l./sc sp. l.	0.93 ± 0.02	0.86–1.03	10	0.90 ± 0.01	0.83–0.96	11
ratio l/wF1	2.88 ± 0.02	2.76–3.05	22	2.90 ± 0.01	2.75–3.03	21
n tubercles F1	35.1 ± 0.4	29–40	35	33.9 ± 0.3	31–40	36
n spinous setae F1	8.7 ± 0.1	8–11	35	8.2 ± 0.1	7–9	37
n tubercles F3	5.1 ± 0.2	2–8	43	4.7 ± 0.2	3–7	40
OVS length	0.34 ± 0.00	0.31–0.36	18	0.34 ± 0.01	0.31–0.39	17
FOS length	0.23 ± 0.00	0.22–0.24	19	0.22 ± 0.00	0.19–0.24	16

***Colour*.** The specific epithet *decoronotus* refers to the colourful notum of the mesothorax with its pattern of brown and blackish brown. This pattern is shown well in the fly from the Thomson’s Falls (Fig. 112), but is much less pronounced to almost absent in flies from Katura Forest. This is not only due to preservation techniques, but is present in live specimens as can be observed in photographs of live flies from Ngare Naro forest (near Thomson’s Falls) where the pattern is also less pronounced (Fig. 117).

***Head*.** Frons mesally depressed, pruinose with glossy spots lateral to ocellar tubercle (Fig. 116), face yellowish brown; occiput mainly blackish brown, ventral edge of occiput and postgena yellowish (Fig. 113), thinly pruinose, with median occipital sclerite densely pruinose; length of outer vertical seta 0.34 mm ± 0.00 (*n* = 35, Tables 4, 7); length of fronto-orbital seta 0.22 mm ± 0.00 (*n* = 35). In highly enlarged photographs, mid facial region densely microtrichose, with row of microtrichia near eye margin (McAlpine 1997: figs 21, 22) (see also Fig. 116).

***Thorax*.** Collar glossy blackish brown (Figs 112, 117); scutum and pleura show typical configuration of blackish brown and brown (Figs 112, 113, 117); humeral callus brown (not blackish brown as described by Feijen (1983: fig. 7)); scutellar spine/scutellum ratio: 0.90 ± 0.01 (*n* = 44, Tables 4, 7); scutellar spine/body length ratio: 0.063 ± 0.001 (*n* = 44); apical seta/ scutellar spine ratio: 0.91 ± 0.01 (*n* = 21, range 0.83–1.03; Table 7); scutellar length/scutellar width (at base) ratio: 0.64; supra-alar carina with supra-alar seta illustrated by McAlpine (1997: fig. 35), seta difficult to see in Figs 112, 113.

***Wing*.** Large, central wing spot, mainly in basal section of cell r4+5, extending into cell br, cell r2+3 and cell bm+dm (Fig. 6); distinct infuscation along central section of vein M4; some variability in intensity of central spot, specimens from Katura forest have, for instance a darker spot than specimens from Naro Moru; two vague apical wing spots mentioned by Feijen (1983) in cells r2+3 and r4+5 often not visible; vein M4 continuing distal of crossvein dm-m in straight line to wing margin; vein CuA+CuP from vein CuP onward extending under angle of 30° to wing margin in almost straight line; cell cua triangular.

***Legs*.** Femur 1 with distinct brown stripe (Figs 113, 114, 117) dorsally on distal half of inner side, not vague as stated by Feijen (1983); femur 1 strongly incrassate, l/w ratio: 2.89 ± 0.01 (*n* = 43, Tables 4, 7); two rows of spinous setae (Fig. 114) on distal two-thirds of femur 1 with 8.4 ± 0.1 setae (*n* = 72, Tables 4, 7), inner row with 4.5 ± 0.1 setae, outer row with 3.9 ± 0.0 setae; two rows of tubercles (Fig. 114) on distal three-quarters of femur 1 with 34.5 ± 0.3 tubercles (*n* = 71, Tables 4, 7), inner row with 16.4 ± 0.1 tubercles and outer row with 18.1 ± 0.2 tubercles; femur 3 (Fig. 115) distally with 4.9 ± 0.1 tubercles (*n* = 83, Tables 4, 7) in single row, except for 1 ♀ and 1 ♂ with each one tubercle in second row; setal formula 3.9, 4.5, 18.1, 16.4, 4.9 agrees well with formula of type-series given by Feijen (1983): 4.0, 4.3, 17.8, 17.0, 4.9.

***Preabdomen*.** Tergites blackish brown, thinly pruinose, with whitish pruinose posterolateral corners; female tergite 7 less dark and glossier; posterolateral corners of tergite 2 more densely pruinose; sternites pale brown; membranous ventral areas with large dark lateral spots (Fig. 117); sternite 1 rounded rectangular, anteriorly strongly constricted mesally (Fig. 118); intersternite 1–2 dark, laterally acuminate, with thin lateral connections to main sternite 2; sternites 2–4 rectangular (Fig. 118), slender, more or less of equal width; sternite 5 somewhat broadening posteriorly; sternite 6 trapezoidal, posteriorly ~ 1.6× as wide as sternites 1–4 (Fig. 118); sternite 4 laterally with two ill-defined more sclerotised sections and two pairs of small heavily sclerotised sections anteriorly (Fig. 118); sternite 5 laterally with more sclerotised sections, anteriorly with pair of strongly sclerotised plates; sternite 6 more vaguely sclerotised laterally and anteriorly with pair of strongly sclerotised plates; sternites 1 and 2 glossy, sternite 1 with some microtrichia laterally, sternite 2 with some microtrichia in posterolateral corners; sternites 3–6 pruinose.

***Female postabdomen*.** Tergite 7 with slightly serrated lateral edges (Fig. 119); 7^th^ spiracle in tergite; anterior sclerite of sternite 7 relatively short with w/l ratio: 3.9 (Table 8); posterior sclerite U-shaped; anterior and posterior sclerites of sternite 7 of ♀ from Ol Doinyo Orok forest similar to paratype illustrated by Feijen (1983), but posterolateral sections of posterior sclerite somewhat expanded (Fig. 119); cercus rather elongate, l/w ratio: 3.6 (Table 8); spermathecae of Ol Doinyo Orok ♀ (Fig. 120) similar to type series.

***Male postabdomen*.** Genitalia identical with illustrations by Feijen (1983) but can be annotated as follows: for ♂ from Ol Doinyo Orok forest, surstylus, subepandrial clasper, cercus and ejaculatory apodeme + sac illustrated (Figs 122–125), as location is furthest removed from localities of type series; median arm of surstylus curved, long (Fig. 122) (due to curve median arm appears slightly shorter in Feijen (1983: fig. 140); outer arm of surstylus with 4 tubercles (and 1 underdeveloped one) (average of 6.0 tubercles (*n* = 4) in Feijen (1983)); median arm and basal half of outer arm clothed in microtrichia on outer side; 3-dimensional character of subepandrial clasper with small differences (compare Figs 122, 124 vs. Feijen 1983: fig. 141), very constricted basal section of subepandrial clasper, mentioned by Feijen (1983), also found in Ol Doinyo Orok ♂; cercus (Fig. 123, Table 8) with broad lateral extension on distal third, apically slightly concave, length/greatest width ratio: 1.4; ejaculatory apodeme + sac/length of body ratio: 16.5% (paratype from Chania Falls with ratio 16.3%) (Figs 125, 153).

***Egg.*** Female from Ol Doinyo Orok forest with two almost fully developed eggs in abdomen. Eggs measured respectively 0.99 mm and 1.00 mm in length with longitudinal ridges spanning from anterior pole to posterior pole; in addition, more simple “lines”. Ridges (indicated with R) with tiny elongate pits along their length, while lines (indicated with L) form more integral part of roughly hexagonal microstructures between ridges (Fig. 121).

#### Distribution and habitat.

The collecting localities are shown on the map for Eastern Africa (Fig. 29). *Centrioncusdecoronotus* was now found at altitudes varying from 1455–2329 m. Feijen (1983) reported it as occurring between 1200–2350 m. The eastern branch of the Great Rift Valley appears to form a barrier between *C.decoronotus* and *C.aberrans*. The gravid female was collected in March at the beginning of the long rains.

#### Remarks.

Feijen (1983) described the lateral sides of female tergite 7 as “rather irregular”, but showed the same serrated edges in his fig. 28, as now found in the specimen from Ol Doinyo Orok (Fig. 119). Other species of *Centrioncus* or *Teloglabrus* with serrated sides of tergite 7 are not known. The paratype from Thomson’s Falls had with 16.3% the highest ejaculatory apodeme + sac/length of body ratio. The Ol Doinyo Orok ♂ had with 16.5% a similar giant ejaculatory apodeme + sac (Table 9).

### ﻿ Centrioncus jacobae

Taxon classificationAnimaliaDipteraDiopsidae

Feijen

D2CDF7F4-994E-51E1-B914-935E0A55BECF

Figs 7 , 127–131 , 132–135 , 136–138 , Tables 5 , 7 , 8 , 9

Centrioncus jacobae Feijen, 1983: 87; Feijen and Feijen 2021: figs 16, 44, 46, 47.

#### Type material.

Malawi, ***holotype*** ♂, Limbe [Blantyre], Mount Soche [15°50'21"S, 35°1'10"E], 1300–1400 m, 22.x.1972, H.R. & J.J. Feijen (RMNH). ***Paratypes***: 3 ♀, 2 ♂, same data as holotype; 7 ♀, 4 ♂, Limbe, Mount Soche, 1300–1400 m, 27.ii.1972; 2 ♀, 3 ♂, Limbe, Mount Soche, 1300–1400 m, 19.iii.1972, H.R. Feijen; 1 ♀, Mount Chiradzulu [Chiradzulu district, near Limbe, 15°41'47.11"S, 35°10'34.20"E], 1300–1350 m, 7.v.1972, H.R. Feijen; 1 ♀, 9 ♂, Limbe, Mount Ndirande [15°45'28"S, 35°3'19"E], 1400 m, 16.xi.1974, H.R. & J.J. Feijen. Next to the holotype, 14 ♀ and 18 ♂ paratypes were traced in the RMNH collections. This is slightly different from the 15 ♀ and 15 ♂ paratypes mentioned by Feijen (1983).

#### Additional material studied.

Malawi: 3 ♀, Limbe, Mount Ndirande [15°45'28"S, 35°3'19"E], 1400 m, 23.iv.1972, H.R. & J.J. Feijen (RMNH); 4 ♀, 5 ♂, Limbe, Mount Soche, [15°50'21"S, 35°1'10"E], 1300–1400 m, 7.viii.1974, H.R. Feijen (RMNH); 1 ♂, Limbe, Mount Soche, 1300–1400 m, 1972–1975, H.R. Feijen (RMNH); 3 ♀, 5 ♂, Limbe, Mount Soche, 1300–1400 m, 19.iii.1972, H.R. Feijen; 3 ♀, 1 ♂, Limbe, Mount Soche, 1300–1400 m, 7.viii.1974, H.R. Feijen; 5 ♀, 5 ♂, Limbe, Mount Soche, 1300–1400 m, 7.i.1973, H.R. Feijen; 3 ♂, Limbe, Mount Ndirande, 1400 m, 10.vii.1975, H.R. & J.J. Feijen. Including the type series, a total of 32 ♀ and 39 ♂ were collected from 1972–1975. This additional material was not included in Feijen (1983) because the author was working in Mozambique and later Zanzibar and did not transport the whole series to those countries.

#### Diagnosis.

**Figures 126–131.**
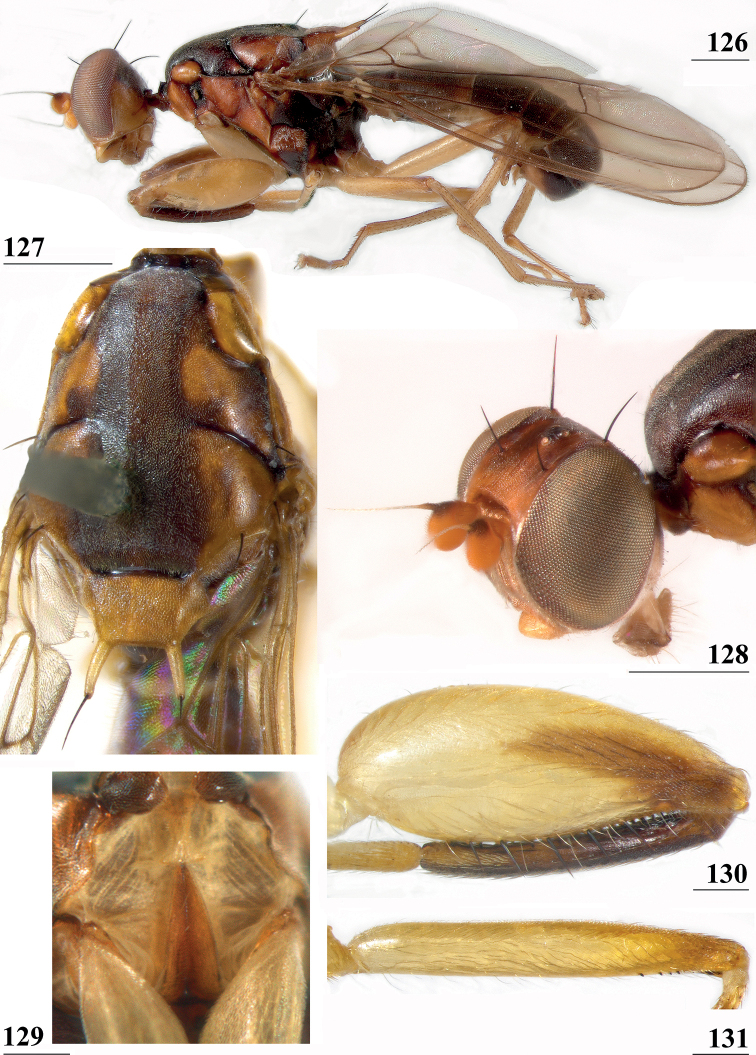
*Centrioncusjacobae*, paratypes **126** Mt Ndirande **127–131** Mt Soche 126 ♂, habitus, lateral view **127** ♀, thorax, dorsal view **128** ♂, head, anterolateral view **129** ♀, basiliform prosternum, ventral view **130** ♀, fore femur, inner view **131** ♂, hind femur, inner view. Scale bars: 0.5 mm (**126–128**); 0.2 mm (**129–131**).

**Figures 132–135.**
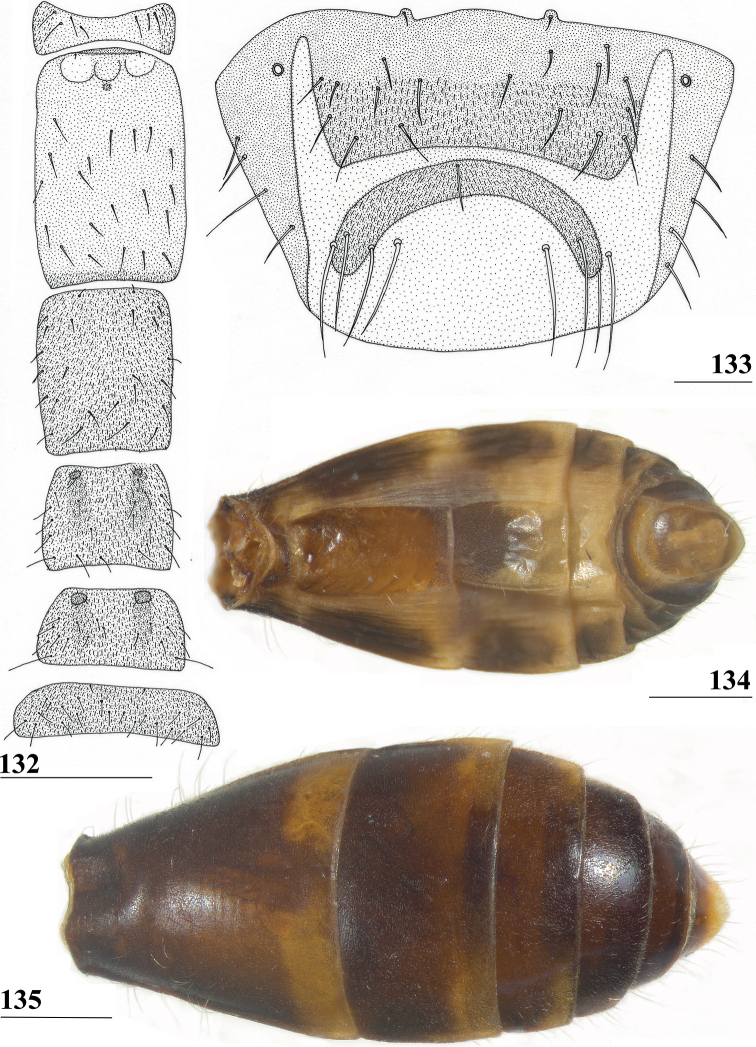
*Centrioncusjacobae*, ♀, paratype, Mt Soche **132** sternites 1–6, ventral view **133** sternite 7, ventral view **134** abdomen, ventral view **135** abdomen, dorsal view. Scale bars: 0.5 mm (**132, 134, 135**); 0.2 mm (**133**).

*Centrioncusjacobae* can be recognised by the mesally slightly depressed pruinose frons with glossy areas (Fig. 128); glossy collar; thinly pruinose scutum with typical configuration of blackish brown and chestnut-brown (Figs 126, 127), brown humeral calli and laterally large brown presutural and postsutural areas; scutellum brown, pale brown scutellar spines; pleura chestnut-brown, blackish area around anterior spiracle, blackish posterior third; scutellar spine/scutellum ratio: 0.78; apical seta/scutellar spine ratio: 1.11; pale, strongly incrassate fore femur (l/w ratio: 2.78) with ~ 33.3 tubercles, inner side with broad brown stripe on distal three-fifths (Fig. 130); large central wing spot (Fig. 7), covering more than basal third of cell r4+5, extending into cells br and bm+dm; tergites blackish brown, two striking, large rectangular pale spots in the posterolateral corners of tergite 2 (Fig. 135); sternite 4 rectangular, sternite 5 slightly broadening posteriorly, sternites 4 and 5 both with anterior pair of small heavily sclerotised spots (Fig. 132); sternite 6 trapezoidal, 1.5× as broad as sternites 1–5; female 7^th^ spiracle in tergite; anterior sclerite of female sternite 7 with w/l ratio: ~ 2.6; posterior sclerite of female sternite 7 smoothly curved, slender, semi-circular U-shaped (Fig. 133); female cercus rather elongate, l/w ratio: 3.2; subanal plate pentagonal, lateral and apical sides straight; spermathecae round, smooth, with dimple, some dispersed tiny tubercles; common base of outer and median arm of surstylus short, broad; outer arm rounded, elongate, basally strongly constricted, largest width 2.7× basal width, apically with ~ 7.6 tubercles (Fig. 138); median arm longer than outer arm, club-shaped, rather broad, apically with ~ 4.0 spinous setae; outer and median arm almost fully clothed in microtrichia on outer side; inner arm much shorter than median arm, apically rounded, halfway with a rounded apophysis (Fig. 138); subepandrial clasper triangular, basal third constricted, medial apical corner pointed, extended lateral corner rounded (Fig. 138); male cercus with a long lateral, parallel-sided extension on distal quarter.

#### Supplementary description.

Biometrical data are given for the remeasured series. Additional morphological data and illustrations are also presented.

***Measurements*.**Feijen (1983) measured 20 ♀and 26 ♂ and gave as results: body length ♀ 5.61 mm ± SE 0.07 (range 5.2–6.2) and ♂ 5.28 mm ± 0.04 (4.8–5.7), head width ♀ 1.19 mm ± 0.01 (1.08–1.28) and ♂ 1.15 mm ± 0.01 (1.00–1.21), wing length ♀5.02 mm ± 0.06 (4.6–5.5) and ♂ 4.70 mm ± 0.05 (4.4–5.1), scutellar spine length ♀ 0.289 mm ± 0.006 (0.25–0.33) and ♂ 0.273 mm ± 0.004 (0.23–0.31). Of the original type series, 10 ♀ and 10 ♂ were remeasured in detail. In Table 5, the new measurements and other quantitative characters are presented. In this table, data are presented for females and males separately. The table shows that differences between females and males for quantitative characters are small. Body length and various other measurements are slightly larger for the females.

**Table 5.** Quantitative characters for *Centrioncusjacobae*. Given are mean ± standard error, range and number of records for females and males. Measurements in mm.

Character	♀			♂		
	x̄ ± SE	range	*n*	x̄ ± SE	range	*n*
head width	1.15 ± 0.02	1.08–1.21	10	1.08 ± 0.01	1.04–1.13	10
body length	5.16 ± 0.06	4.82–5.43	10	4.78 ± 0.06	4.58–5.25	10
wing length	4.44 ± 0.05	4.15–4.64	10	4.11 ± 0.05	3.78–4.39	10
sc. sp. length	0.28 ± 0.01	0.24–0.31	10	0.26 ± 0.00	0.24–0.28	10
apical seta length	0.30 ± 0.01	0.28–0.34	8	0.29 ± 0.01	0.27–0.31	9
scutellum length	0.36 ± 0.01	0.33–0.39	9	0.32 ± 0.01	0.31–0.36	10
head w./body l.	0.22 ± 0.00	0.22–0.24	10	0.23 ± 0.00	0.22–0.24	10
sc. sp. l./body l.	0.054 ± 0.001	0.050–0.057	9	0.054 ± 0.001	0.051–0.058	10
sc. sp. l./sc. length	0.77 ± 0.01	0.73–0.83	9	0.80 ± 0.01	0.77–0.85	10
ap. seta l./sc. sp. l.	1.09 ± 0.02	1.00–1.17	8	1.12 ± 0.02	1.00–1.20	9
ratio l/wF1	2.73 ± 0.02	2.64–2.81	9	2.83 ± 0.02	2.74–2.92	9
n tubercles F1	34.0 ± 0.4	30–37	19	32.4 ± 0.4	30–35	17
n spinous setae F1	8.9 ± 0.1	8–10	19	8.9 ± 0.2	7–10	20
n tubercles F3	6.0 ± 0.3	4–9	20	6.1 ± 0.4	4–10	17
OVS length	0.34 ± 0.01	0.29–0.39	9	0.32 ± 0.01	0.29–0.37	10
FOS length	0.24 ± 0.01	0.20–0.29	9	0.20 ± 0.01	0.16–0.23	9

The original measurements of the type series do not agree well with the measurements now recorded. The measurements now found for ♀ and ♂ combined are: body length 4.97 mm ± 0.06 (range 4.58–5.43, *n* = 20), head width 1.11 mm ± 0.01 (range 1.04–1.21, *n* = 20), wing length 4.28 mm ± 0.05 (range 3.78–4.64, *n* = 20), scutellar spine length 0.27 mm ± 0.00 (range 0.24–0.29, *n* = 20). This means that the present measurements for body length and wing length are ~ 10% less, while the measurements for head width and scutellar spine are ~ 5% less. The specimens of the type series were measured in Malawi while in fresh condition. This could only affect the body length measurements to a small extent but not the other measurements. The persistent differences are probably due to calibration errors. The new measurements now indicate *C.jacobae* as a species is clearly smaller than the other *Centrioncus* species for which large series could be measured. (Tables 6, 7).

**Table 6.** Quantitative characters for *Centrioncusaberrans* and *Centrioncusbytebieri* for both sexes combined. Measurements in mm.

Character	*C.aberrans* ♀ & ♂			*C.bytebieri* ♀ & ♂		
	x̄ ± SE	range	*n*	x̄ ± SE	range	*n*
head width	1.19 ± 0.01	1.11–1.25	23	1.16 ± 0.01	1.06–1.22	16
body length	5.16 ± 0.04	4.82–5.55	23	5.55 ± 0.09	4.94–6.16	16
wing length	4.72 ± 0.03	4.27–4.88	23	5.04 ± 0.06	4.39–5.34	16
sc. sp. length	0.29 ± 0.00	0.25–0.31	23	0.36 ± 0.01	0.29–0.43	16
ap. seta length	0.32 ± 0.00	0.29–0.34	10	0.42 ± 0.01	0.36–0.46	12
scutellum length	0.37 ± 0.00	0.34–0.39	18	0.38 ± 0.01	0.31–0.42	16
head w./body length	0.23 ± 0.00	0.22–0.25	23	0.21 ± 0.00	0.19–0.22	16
sc. sp. l./body length	0.056 ± 0.001	0.051–0.062	22	0.064 ± 0.001	0.055–0.075	16
sc. sp. l./sc. length	0.78 ± 0.01	0.73–0.87	18	0.95 ± 0.01	0.86–1.06	16
ap. seta l./sc. sp. l.	1.13 ± 0.01	1.08–1.18	10	1.14 ± 0.02	1.03–1.25	12
F1 – ratio l/w	2.75 ± 0.01	2.68–2.84	22	3.30 ± 0.02	3.17–3.43	16
F1 – n tubercles	35.4 ± 0.5	29–41	42	32.7 ± 0.6	26–38	31
F1 – n spinous setae	9.0 ± 0.1	8–10	45	6.8 ± 0.2	5–10	31
F3 – n tubercles	6.5 ± 0.2	4–11	46	4.9 ± 0.2	3–7	32
OVS length	0.36 ± 0.00	0.29–0.39	17	0.38 ± 0.01	0.34–0.43	16
FOS length	0.22 ± 0.01	0.18–0.25	16	0.28 ± 0.00	0.27–0.31	16

**Table 7.** Quantitative characters for *Centrioncusdecoronotus* and *Centrioncusjacobae* for both sexes combined. Measurements in mm.

Character	*C.decoronotus* ♀ & ♂			*C.jacobae* ♀ & ♂		
	x̄ ± SE	range	*n*	x̄ ± SE	range	*n*
head width	1.14 ± 0.01	1.02–1.24	44	1.11 ± 0.01	1.04–1.21	20
body length	5.15 ± 0.04	4.64–5.61	44	4.97 ± 0.06	4.58–5.43	20
wing length	4.51 ± 0.03	4.09–5.00	39	4.28 ± 0.05	3.78–4.64	20
sc. sp. length	0.33 ± 0.00	0.29–0.37	44	0.27 ± 0.00	0.24–0.29	20
ap. seta length	0.30 ± 0.01	0.24–0.36	21	0.30 ± 0.00	0.27–0.34	17
scutellum length	0.36 ± 0.00	0.31–0.41	44	0.34 ± 0.01	0.31–0.39	19
head w./body length	0.22 ± 0.00	0.20–0.25	44	0.22 ± 0.00	0.21–0.24	20
sc. sp. l./body length	0.063 ± 0.001	0.056–0.072	44	0.054 ± 0.000	0.050–0.058	20
sc. sp. l./sc. length	0.90 ± 0.01	0.76–1.00	44	0.78 ± 0.01	0.73–0.85	19
ap. seta l./sc. sp. l.	0.91 ± 0.01	0.83–1.03	21	1.11 ± 0.02	1.00–1.20	17
F1 – ratio l/w	2.89 ± 0.01	2.75–3.05	43	2.78 ± 0.02	2.64–2.92	18
F1 – n tubercles	34.5 ± 0.3	29–40	71	33.3 ± 0.3	30–37	36
F1 – n spinous setae	8.4 ± 0.1	7–11	72	8.9 ± 0.1	7–10	39
F3 – n tubercles	4.9 ± 0.1	2–8	83	6.0 ± 0.2	4–10	37
OVS length	0.34 ± 0.00	0.31–0.39	35	0.33 ± 0.01	0.29–0.39	19
FOS length	0.22 ± 0.00	0.19–0.24	35	0.22 ± 0.01	0.16–0.29	18

In connection with ecological research in Malawi from 1971 to 1975, the weight of fresh flies was regularly determined. The weight of some *C.jacobae* flies was also determined. In August (in the non-reproductive phase) seven females weighed on average 4.2 mg, while six males weighed on average 3.4 mg.

***Colour*.** In optimal conditions, this species presents a colourful pattern of blackish brown and chestnut brown on the thorax as described by Feijen (1983) and now illustrated (Figs 126, 127).

***Head*.** Colour pattern of anterior and posterior sides of head shown in Figs 126, 128; length of outer vertical seta 0.33 mm ± 0.01 (*n* = 19), length of fronto-orbital seta 0.22 mm ± 0.01 (*n* = 18).

***Thorax*.** Basiliform prosternum brown, slender, triangular and anteriorly acuminate (Fig. 129); scutellar spine/scutellum ratio: 0.78 ± 0.01 (*n* = 19, Tables 5, 7); scutellar spine/body length ratio: 0.054 ± 0.000 (*n* = 20); apical seta/scutellar spine ratio: 1.11 ± 0.02 (*n* = 17), Feijen (1983) described apical seta as being “as long as spine”, but seta is now shown to be slightly longer than spine; scutellar length/scutellar width (at base) ratio: 0.68.

***Wing*.** Pale brownish with distinct, large central wing spot, covering more than basal third of cell r4+5 (just past crossvein dm-m), slightly extending into apex of cell br and extending into anterior section of cell bm+dm between crossveins (Fig. 7); distinct infuscation along crossvein dm-m and along vein M4 between cell cua and crossvein dm-m; vein CuA+CuP from vein CuP onward extending under angle of 30° to wing margin in almost straight line (very sightly curving downward); vein M4 continuing distal of crossvein dm-m in almost straight line to wing margin; cell cua triangular (Fig. 7).

***Legs*.** Femur 1 (Figs 126, 130) strongly incrassate, l/w ratio: 2.78 ± 0.02 (*n* = 18, Tables 5, 7); two rows of spinous setae on distal two-thirds of femur 1 with 8.9 ± 0.1 setae (*n* = 39; Tables 5, 7), inner row with 4.9 ± 0.1 setae and outer row with 4.0 ± 0.1 setae; two rows of tubercles on distal three-quarters of femur 1 with 33.3 ± 0.3 tubercles (*n* = 36), inner row with 15.4 ± 0.2 tubercles and outer row with 17.8 ± 0.2 tubercles; femur 1 on inner side with broad brown stripe on distal three-fifths (Fig. 130); femur 3 (Fig. 131) distally with 6.0 ± 0.2 (*n* = 37), tubercles in single row but one specimen with one tubercle placed in second row. Results lead to setal formula: 4.0, 4.9, 17.8, 15.4, 6.0 which agrees well with formula of the type-series as given by Feijen (1983): 4.0, 4.7, 16.9, 15.4, 5.6.

**Figures 136–138.**
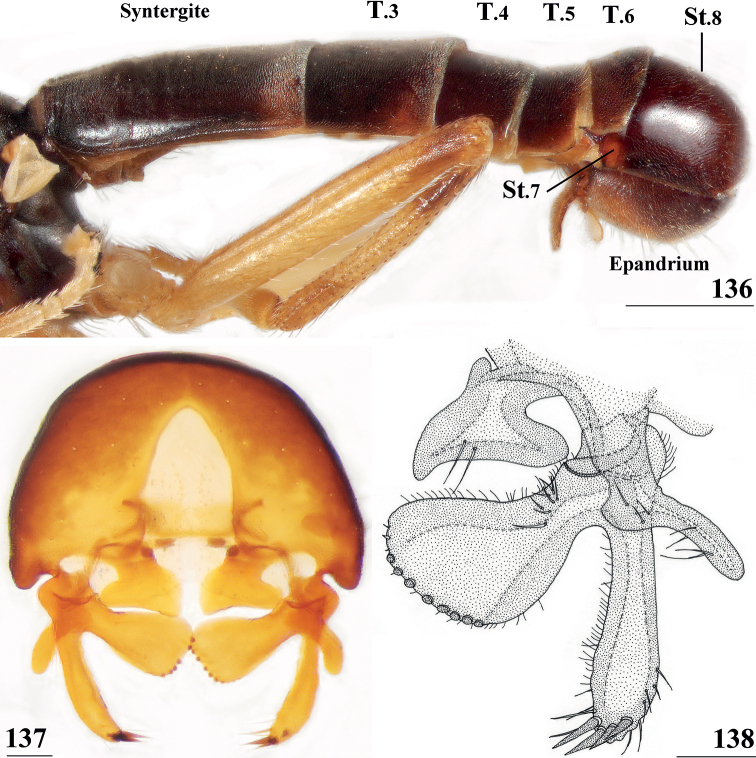
*Centrioncusjacobae*, ♂, Mt Soche **136** holotype, abdomen, hind femur, lateral view **137** epandrium, surstyli, posterior view **138** paratype, subepandrial clasper and surstylus, inner view. Scale bars: 0.5 mm (**136**); 0.1 mm (**137, 138**).

***Preabdomen*.** Tergites blackish brown (Figs 135, 136), almost glossy, but with some pruinosity, especially at posterior margins of tergites 2 and 3 (Fig. 136); tergite 2 with two striking, large, pale brown rectangular areas in posterolateral corners (Fig. 135); smaller pale brown spots in posterolateral corners of tergites 3, 4, and 5; sternites 1 and 2 and anterior half of sternite 3 dark brown, posterior half of sternite 3 and sternites 4–6 yellowish brown (Fig. 134); sternite 1 glossy but laterally with some pruinosity; sternite 2 glossy with pruinose posterior margin; sternites 3–6 pruinose (Figs 132, 134); membranous ventral areas with large dark lateral spots (Fig. 134); sternite 1 somewhat rectangular, with concave anterior and posterior sides (Fig. 132); intersternite 1–2 dark, laterally acuminate, with thin lateral connections to main sternite 2; sternite 3 rectangular; sternite 4 rectangular, sternite 5 slightly broadening posteriorly, both sternites with pair of tiny heavily sclerotised spots, and with slightly more sclerotised areas posterior to spots (Fig. 132); sternite 6 ~ 1.5× as broad as sternites 1–5, with convex lateral sides and somewhat broadening posteriorly.

***Female postabdomen*.** Tergite 7 brown anteriorly and yellowish brown posteriorly (Fig. 135); anterior sclerite of sternite 7 rectangular, w/l ratio: ~ 2.6 (Figs 133, 134, Table 8), dark brown and glossy on anterior half, more yellowish brown and pruinose on posterior half; posterior sclerite of sternite 7 slender, rounded, U-shaped (Fig. 133), clothed in microtrichia and with four setulae at posterior apices; cercus rather elongate, l/w ratio: 3.2 (Table 8); 7^th^ spiracle in tergite (Fig. 133).

**Table 8.** Ratio width/length for the anterior sclerite of ♀ sternite 7, ratio length/width for ♀ cercus and ratio length/greatest width for ♂ cercus. Species are arranged according to three species-groups recognised.

*Centrioncus*	♀ anterior plate of sternite 7, ratio w/l	♀ cercus, ratio l/w	♂ cercus, ratio length/greatest width
*angusticercus*	4.4	5.4	-
*aberrans*	5.4	5.1	2.6
*crassifemur* sp. nov.	8.6	4.3	-
*bururiensis* sp. nov.	2.6	4.5	2.4
*decellei*	2.7	4.1	-
*copelandi* sp. nov.	2.9	4.4	1.5
*prodiopsis*	2.5	4.0	1.4
*bytebieri*	2.6	2.3	1.7
*decoronotus*	3.9	3.6	1.4
*jacobae*	2.6	3.2	1.4

***Male postabdomen*.** Lateral side of male postabdomen shown in Fig. 136; epandrium, surstylus and subepandrial clasper presented in Figs 137, 138; common base of outer and median arm of surstylus short, broad; outer arm rounded, elongate, basally strongly constricted, largest width 2.7× basal width, apically with 7.6 (range 7–9) tubercles (Figs 137, 138); median arm long (longer than outer arm), club-shaped, rather broad, apically with 4.0 (range 3–5) stout spinous setae; outer and median arm almost fully clothed in microtrichia on outer side; inner arm much shorter than median arm, apically rounded, halfway with a rounded apophysis (Fig. 138); subepandrial clasper triangular, basal third constricted, medial-apical corner pointed, extended lateral corner rounded (Figs 137, 138); cercus with broad lateral extension on distal third, length/greatest width ratio: 1.4 (Table 8); ejaculatory apodeme + sac very large, 11–14% of body length (Table 9).

**Table 9.** Length of ejaculatory apodeme + sac, length of body and ratio ejaculatory apodeme + sac/length of body for *Centrioncus* and *Teloglabrus* species. Measurements in mm, ratio as %. For some species, values are given for more than one specimen, partly from different locations.

Genus	Species	Origin	Apodeme + sac	Length of body	Apodeme + sac/body
*Centrioncus*	*aberrans*	Kenya, Timboroa	0.60	5.37	11.2
	*aberrans*	Kenya, Mt Elgon	0.52	5.12	10.1
	*aberrans*	Kenya, Mt Elgon	0.52	5.12	10.1
	*aberrans*	Rwanda, Lac Gando	0.48	5.10	9.3
	*bururiensis* sp. nov.	Burundi, Bururi For.	0.48	4.64	10.4
	*bytebieri*	Kenya, Vuria For.	0.61	5.00	12.2
	*copelandi* sp. nov.	Kenya, Kasigau Mt	0.51	5.00	10.1
	*decoronotus*	Kenya, Chania Falls	0.85	5.20	16.3
	*decoronotus*	Kenya, Ol Doinyo Orok	0.84	5.10	16.5
	*jacobae*	Malawi, Mt Soche	0.74	5.28	14.0
	*jacobae*	Malawi, Mt Soche	0.56	5.28	10.7
	*prodiopsis*	Tanzania, Kilimanjaro	0.60	5.00	12.0
*Teloglabrus*	*australis*	South Africa, G. of Eden	0.39	4.60	8.4
	*curvipes*	South Africa, Ingeli For.	0.33	5.00	6.5
	*duplospinosus*	South Africa, Lions Bush	0.33	4.90	6.6
	*entabenensis*	South Africa, Entabeni For.	0.35	5.00	7.0
	*lebombensis*	South Africa, Gwaleni For.	0.39	5.30	7.3
	*londti*	South Africa, Grahamstown	0.30	5.20	5.8
	*milleri*	South Africa, Nkandla For.	0.33	5.00	6.5
	*pelecyformis*	South Africa, Town Bush	0.40	5.50	7.3
	*prolongatus*	South Africa, Deepdale	0.31	4.70	6.6
	*sabiensis*	South Africa, Frankfurt For.	0.36	5.00	7.3
	*sanorum*	South Africa, Drakensberg	0.31	4.92	6.4
	*sanorum*	South Africa, Drakensberg	0.30	4.92	6.1
	*stuckenbergi*	South Africa, Mariepskop	0.36	6.50	5.6
	*tsitsikamensis*	South Africa, Tsitsikama	0.39	4.60	8.4
	*trituberculatus*	Mozambique, Gorongosa Mt	0.34	5.50	6.1
	*vumbensis*	Zimbabwe, Vumba Mt	0.35	5.20	6.7

#### Distribution and habitat.

This species occurs at altitudes between 1300 and 1400 m in rain forests on the mountains around Blantyre and Limbe in Malawi. The rain forest at Mount Ndirande is gone, but patches on Mount Soche remain. The largest patches of rain forest remain on Mount Chiradzulu. However, this latter remark is quite relative when Loveridge’s (1954) observations about the Chiradzulu forest are considered: “Unfortunately the forest was but a pitiful remnant of its former self, few of its trees exceeding thirty feet in height and even these patches of secondary growth were separated by bracken which had swept up from below” and “This deforestation has resulted in the drying up of many of the streams during much of the year”. The strong discontinuity of the habitat of Afromontane Forest Flies will obviously be reinforced by the ongoing deforestation.

In the period 1971–1975, extensive collecting of Diopsidae was carried out in Malawi. Regular sampling took place on other mountains in Malawi, like the Nyika Plateau, Ntchisi Mountain, Dedza Mountain, Zomba Plateau and Mount Mulanje. However, *Centrioncus* were never encountered on these other mountains.

#### Remarks.

Feijen (1983) remarked that *Centrioncusjacobae* was every now and then collected together with other diopsids, especially *Diopsisphlogodes* Hendel. However, this was a misidentification of a species of the *D.cruciata* species-group. *Diopsisphlogodes* is a junior synonym of the rice stem-borer *D.longicornis* Macquart (see Feijen 1985).

### ﻿ Centrioncus prodiopsis

Taxon classificationAnimaliaDipteraDiopsidae

Speiser

A42B13D1-4836-5FBB-9F9E-AE6F61D4D2C8

Figs 8 , 29 , 139–145 , 146–149 , 150, 151 , 152 , Tables 8 , 9

Centrioncus prodiopsis Speiser, 1910: 191; Hennig in litt. 1950 (Shillito archive, NHMUK); Feijen 1983: 80; De Meyer 2004: 28.

#### Type series.

Tanzania, ***lectotype*** ♂ (designated by Feijen 1983), Kibonoto [Kibongoto 3°11'S, 37°06'E], Kilimandjaro [Kilimanjaro], 7.x.1905–06, 2000–3500 m, Y. Sjöstedt (NHRS). ***Paralectotypes***: 1 ♀, same locality, 5.i.1905–06, 2000 m, Y. Sjöstedt (NHRS); 1 ♀ (no abdomen), same locality, ix.1905–06, 1300–1900 m, Y. Sjöstedt (NHRS); 1 ♂ (no abdomen, but genitalia drawn by Hennig prior to1950, who marked the drawings with “holotype *C.prodiopsis*”), same locality, 5.i.1905–06, 2000 m, Y. Sjöstedt (ZMHB).

#### Material studied.

Kenya: 2 ♀, Rift Valley Prov., Olloitokitok [Oloitokitok], malaise trap, indigenous forest, 2.94456°S, 37.50714°E, 11–25.xi.2011, 1853 m, R. Copeland (ICIPE).

#### Diagnosis.

*Centrioncusprodiopsis* can be recognised by mesally slightly depressed pruinose frons with two glossy spots; glossy collar; pruinose, blackish brown scutum, brown humeral calli; blackish brown scutellum with brown lateral sides; blackish brown pleura, brown propleuron with paler ventral half; scutellar spine/scutellum ratio: ~ 0.98; apical seta/scutellar spine ratio: ~ 1.02; pale, strongly incrassate fore femur (l/w ratio: 2.92) with ~ 31.1 tubercles, on inner side with brown stripe on distal two-fifths (Fig. 143); very large central wing spot (Fig. 8), covering nearly basal half of cell r4+5, extending into distal half of cell br, extending into distal two-thirds of cell bm+dm and well into cells r2+3, m1 and m4; tergites 1–6 uniformly blackish brown; female 7^th^ spiracle in tergite; sternite 4 rounded, anteriorly significant, rounded invagination; sternite 5 rounded trapezoidal, anteriorly significant, rounded invagination; sternites 4 and 5 with anterior pair of small heavily sclerotised spots (Fig. 146); sternite 5 half-length of sternite 4; sternite 6 trapezoidal, 1.4× as broad as sternites 1–5; anterior sclerite of sternite 7 trapezoidal, w/l ratio: ~ 2.5, posterior sclerite of sternite 7 rounded, broad U-shaped (Figs 147, 148); female cercus rather elongate, l/w ratio: 4.0; subanal plate pentagonal, laterally convex and with acuminate apex; smooth spermathecae, very large apical dimple, cup-shaped, some dispersed tiny tubercles; common base of outer and median arms of surstylus short, broad; outer arm trapezoidal, straight lateral sides, broadening apically to twice width at base, apically with row of 17 tubercles (Fig. 150); median arm slender, parallel-sided, slightly curved rod, shorter than outer arm, apically with six setulae, no spinous setae; outer arm and basal half of median arm clothed in microtrichia on outer side; inner arm short, ca. half-length of other arms, with bulbous preapical apophysis; subepandrial clasper elongate, narrow, hardly constricted at base, apical corners rounded, apically hardly convex (Fig. 151); male cercus basally narrow, broadening apically, with broad lateral extension.

**Figures 139–145.**
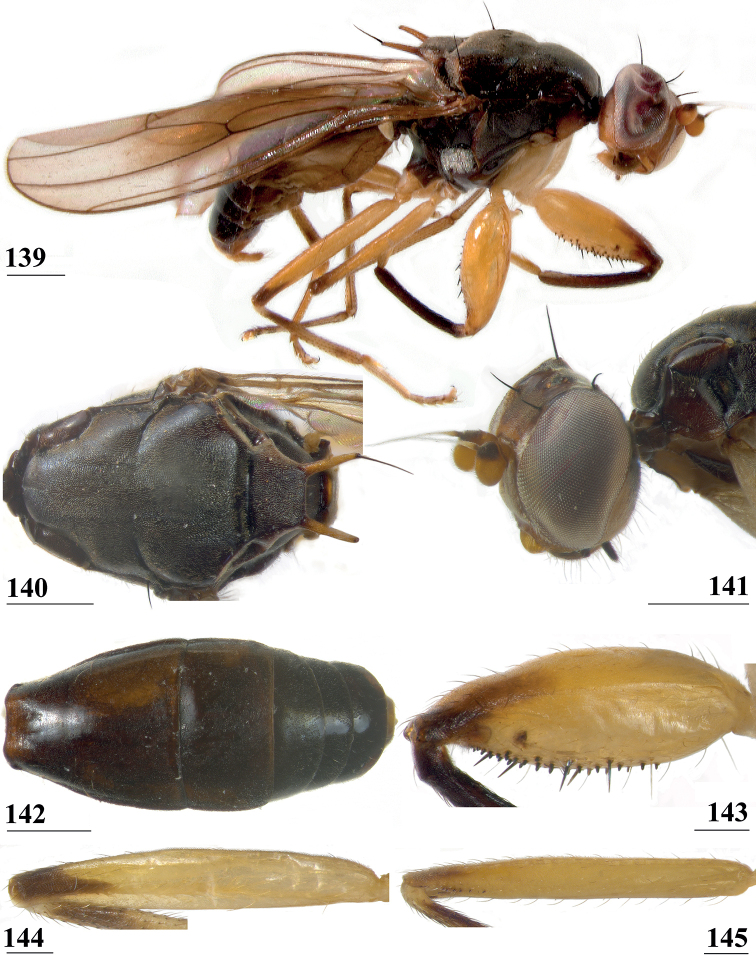
*Centrioncusprodiopsis*, ♀, Oloitokitok **139** habitus, lateral view **140** thorax, dorsal view **141** head, anterolateral view **142** abdomen, dorsal view **143** fore femur, inner view **144** hind femur, outer view **145** hind femur, inner view. Scale bars: 0.5 mm (**139–142**); thorax, head, abdomen), 0.2 mm (**143–145**).

**Figures 146–149.**
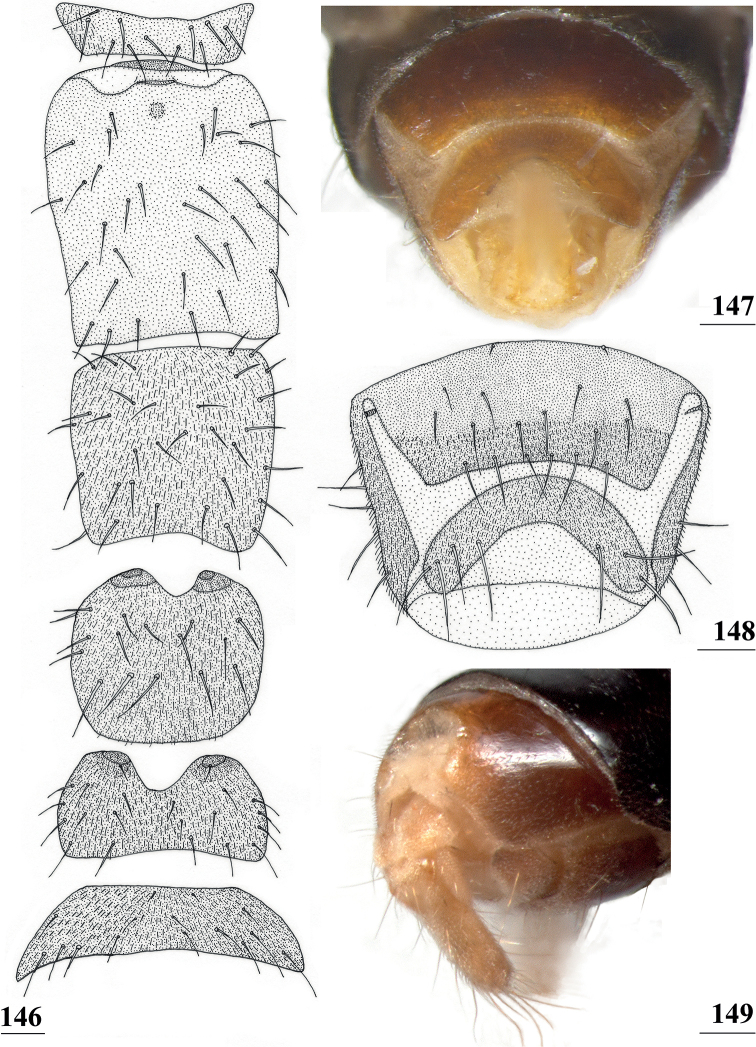
*Centrioncusprodiopsis*, ♀, Oloitokitok **146** sternites 1–6, ventral view **147, 148** sternite 7, ventral view **149** tergite 7 and cerci, posterolateral view. Scale bars: 0.5 mm (**146**); 0.1 mm (**147–149**).

#### Supplementary description.

Feijen (1983) studied the ♂ lectotype and one ♀ paralectotype. The redescription by Hennig (in litt.) of the specimen of the type series in ZMHB also became available. Now two additional females from Kenya could be examined, but one of these two flies was rather teneral. Updated biometrical data are now given. Additional morphological data and illustrations are presented.

***Measurements*.**Speiser (1910) provided length for type series as 4¾–5¼ mm. Length of body 5.9 mm (♀ paralectotype), 4.9 mm (Kenya ♀) and 5.0 mm (♂ lectotype), width of head 1.20 mm (♀ paralectotype), 1.08 and 1.04 (Kenya ♀♀), and 1.06 mm (♂ lectotype), length of wing 5.4 mm (♀ paralectotype), 4.5 mm (Kenya ♀) and 4.6 mm (♂ lectotype), length of scutellar spine 0.37 mm (♀ paralectotype), 0.34 and 0.36 (Kenya ♀♀), and 0.29 mm (♂ lectotype).

***Head*.** As in lectotype and paralectotype, Kenya specimens with frons slightly depressed mesally, anterior quarter of pruinose frons less dark brown and on either side of ocellar tubercle with glossy spot (Fig. 141); length of outer vertical seta 0.31 mm (*n* = 2), length of fronto-orbital seta 0.22 mm (*n* = 2).

***Thorax*.** Collar dark brown, mainly glossy; basiliform prosternum dark brown, slender, triangular and anteriorly acuminate; scutum blackish brown and pruinose, humeral callus brown and more glossy (Figs 139–141), Speiser (1910) stated humeral callus dark mahogany red, but was not visible in Feijen (1983); scutellum blackish brown, pruinose, lateral sides and spines brown (Figs 139, 140); colouration of pleura of Kenya specimens corresponds to description of lectotype and paralectotype by Feijen (1983), especially more brownish propleuron with paler ventral half (Fig. 139); densely pruinose spot on katepisternum (also mentioned by Speiser) distinct (Fig. 139); basiliform prosternum dark brown, slender, triangular and anteriorly acuminate; scutellar spine/scutellum ratio: 0.98 (*n* = 2, range 0.97–1.00); scutellar spine/body length ratio: 0.069 (*n* = 1); two Kenya specimens with apical seta/scutellar spine ratio: 1.02 (range 1.00–1.04) [Feijen (1983) described apical seta as “slightly larger than spine”]; scutellar length/scutellar width (at base) ratio: 0.64.

***Wing*.** Almost transparent with very large, distinct, brownish central spot covering nearly basal half of cell r4+5 (well past crossvein dm-m), extending into distal half of cell br and extending into distal two-thirds of cell bm+dm and well into cells r2+3, m1 and m4 (Fig. 8); vein CuA+CuP from vein CuP onward extending under angle of 30° to wing margin in almost straight line; vein M4 continuing distal of crossvein dm-m in almost straight line to wing margin; cell cua subtriangular (Fig. 8).

***Legs*.** Femur 1 (Fig. 143) strongly incrassate, l/w ratio: 2.92 (*n* = 1); two rows of spinous setae on distal two-thirds of femur 1 with 7.3 ± 0.2 setae (*n* = 8), inner row with 3.9 ± 0.1 setae, outer row with 3.4 ± 0.2 setae; two rows of tubercles on distal three-quarters of femur 1 with 31.1 ± 0.6 tubercles (*n* = 8), inner row with 15.4 ± 0.3 tubercles, outer row with 15.8 ± 0.4 tubercles; femur 1 yellowish brown with on inner side brown stripe on distal two-fifths (Fig. 143); femur 3 (Fig. 145) distally with 3.9 ± 0.4 (*n* = 8) tubercles; setal formula 3.4, 3.9, 15.8, 15.4, 3.9 which agrees with formula of type-series as given by Feijen (1983): 3.5, 3.8, 15.8, 14.3, 3.0; femur 3 yellowish with on outer side brown stripe on distal quarter and on inner side vague brown spot apically (Figs 144, 145).

***Preabdomen*.** Tergites uniformly blackish brown (Fig. 142), almost glossy, laterally with some pruinosity (Fig. 139); sternites brownish (Fig. 139); sternite 1 glossy but laterally with some pruinosity, sternite 2 glossy, sternites 3–6 pruinose (Fig. 146); membranous ventral areas with dark lateral spots; sternite 1 trapezoidal, anteriorly broader (Fig. 146); intersternite 1–2 dark, laterally acuminate, with thin lateral connections to main sternite 2; sternite 3 rectangular with rounded corners; sternites 4 and 5 anteriorly rounded, with significant rounded invagination, both sternites with pair of small heavily sclerotised spots anteriorly (Fig. 146); sternite 5 half-length of sternite 4; sternite 6 broadening posteriorly, ~ 1.4× as broad as sternites 1–5, with convex lateral sides.

***Female postabdomen*.** Tergite 7 brown, paler brown along posterior margin (Fig. 149); anterior sclerite of sternite 7 trapezoidal, narrowing posteriorly, w/l ratio: ~ 2.5 (Figs 147, 148, Table 8), brown but paler along posterior margin, glossy on anterior two-thirds, pruinose on posterior one-third; posterior sclerite of sternite 7 rounded, broadly U-shaped (Figs 147, 148), clothed in microtrichia and with 4–6 setulae at both posterior apices; cercus (Fig. 149) rather elongate, l/w ratio: 4.0 (Table 8); 7^th^ spiracle at edge of tergite (Fig. 148).

**Figures 150, 151.**
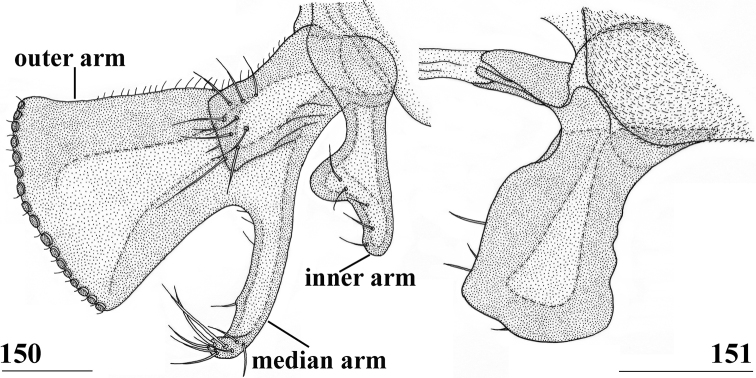
*Centrioncusprodiopsis*, ♂, lectotype, Kibongoto **150** surstylus, inner view **151** subepandrial clasper and base of surstylus, posterior view. Scale bars: 0.1 mm.

***Male postabdomen*.** Outer and median arms of surstylus well separated, with short, broad common base (Fig. 150); outer arm somewhat trapezoidal with straight lateral sides, broadening apically to almost twice width at base, apically with row of 17 tubercles with tiny setulae in between, with rectangular, raised section with 11 setulae basally on inner side (Fig. 150), outer arm wholly clothed with microtrichia on outer side; median arm slender, parallel-sided, slightly curved rod-shaped, slightly shorter than outer arm, apically with 4 small setulae and 2 long setulae, no spinous setae present, basal half of outer side covered with microtrichia; inner arm of surstylus short, ca. half-length of other two arms, with a bulbous preapical apophysis, apically with five setulae (Fig. 150); subepandrial clasper (Fig. 151) elongate, slightly constricted at base, narrow, somewhat rectangular, apically with rounded corners, apical edge slightly convex, glabrous, with three long setulae centrally on inner side and four small hairs at apical edge; cercus basally narrow, broadening apically, with short, broad lateral extension, length/greatest width ratio: 1.4 (Table 8); ejaculatory apodeme + sac very large, 12% of body length (Table 9).

#### Distribution and habitat.

The collecting localities are shown on the map for Eastern Africa (Fig. 29). The type locality is on the southern side of the Kilimanjaro region, while the Kenyan specimens are from the northern side of the Kilimanjaro region. In a straight line, the distance between the two localities is 55 km.

#### Remarks.

Speiser (1910) based his description on two pairs of specimens. Feijen (1983) used for his redescription one pair from NHRS. In the NHRS collection, there was one additional fly without an abdomen, while ZMHB also housed one fly without an abdomen. Feijen designated the ♂ of the pair studied as lectotype and the ♀ as paralectotype. Now, a typed letter from W. Hennig, dated 3.xii.1947, was recovered from the J.F. Shillito archive (NHMUK). The section relevant to *Centrioncus* ran as follows:

Ueber den Holotypus von Centrioncusprodiopsis Speiser, der sich in der Sammlung des Zoologischen Museums Berlin befindet, habe ich mir früher einmal, für den Fall, dass er zerstört werden sollte, Notizen gemacht. Kopien meiner Zeichnungen fuge ich für Sie diesem Briefe bei. Sie können diese Zeichnungen beliebig verwenden. Auch gegen Veröffentlichung habe ich nichts einzuwenden, da wir hier doch in absehbare Zeit nicht dazu kommen.

Ueber die systematische Stellung von Centrioncusprodiopsis habe ich mir noch kein abschliessendes Urteil gebildet. Ich weiss nur sicher, dass die Art nicht zu den Sepsiden, und auch nicht zu den Megamerinidae gehört. Gegen Ihre Ansicht, dass es sich um eine Diopside handelt, wäre von der morphologie des Kopulationsapparates her wohl kaum etwas einzuwenden. Da ich selbst aber die Verwandtschaftsgruppen um die Sapromyzidae noch nicht genügend untersucht habe, möchte ich mit meinem Urteil vorläufig zurückhalten. Von Kopfborsten sind nur 1 Vertikalborste und 1 Frontorbitalborste vorhanden, von Thorakalborsten 1 Notopleuralborste (die hintere), 1 Supraalar- und 1 Postalarborste.

[In a brief translation this comes to: Regarding the holotype of *Centrioncusprodiopsis* in ZMHB, I made years ago notes in case it would be destroyed. I enclose copies of the drawings I made. If you like, you can use these for publication. I have not yet formed a conclusive judgment on the systematic position of *Centrioncusprodiopsis*. I only know for sure that the species does not belong to the Sepsidae or Megamerinidae. In view of the morphology of the copulation apparatus, there would hardly be anything to object to your view that it is a diopsid. However, I would like to reserve my judgment for the time being. On the *Centrioncus* head 1 vertical seta and 1 fronto-orbital seta are present, while the thorax counts 1 notopleural seta (the posterior one), 1 supra-alar seta and 1 postalar seta.]

**Figure 152.**
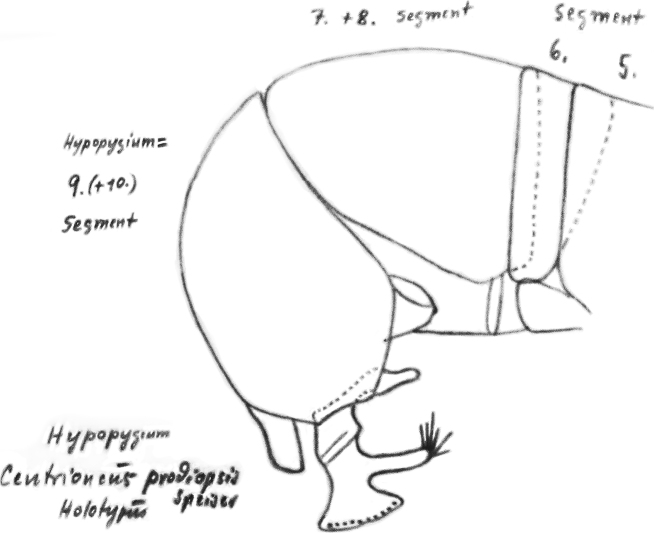
*Centrioncusprodiopsis*, ♂, abdomen segments 5–8, epandrium and surstylus, lateral view. This is a (restored) drawing made by Willi Hennig in the late 1930s. Hennig was probably not aware of the three ZMHB specimens and assumed the NHRS specimen to be the holotype. The NHRS specimen is now designated as paralectotype.

From this letter, it became obvious that the fly without abdomen in ZMHB is a male, so the fly without abdomen in NHRS has to be a female. Hennig indicated the ZMHB fly as the holotype. However, Speiser did not designate a holotype. All four specimens of the type series were labelled as syntypes (Feijen 1983). It seems likely that Hennig was not aware of the three NHRS flies. As such, the designation of the lectotype by Feijen (1983) remains valid. Hennig’s drawings of the ZMHB fly showed that this male was definitely conspecific with the lectotype. As, since the type series of 1905–1906, male specimens of *C.prodiopsis* have apparently never been collected again, it is considered useful to copy Hennig’s drawing of the male postabdomen (Fig. 152) as an addition to Feijen’s (1983) drawings and for historical reasons. In this drawing, Hennig’s original labels are indicated. The ZMHB specimen is now designated as male paralectotype.

**Figures 153, 154.**
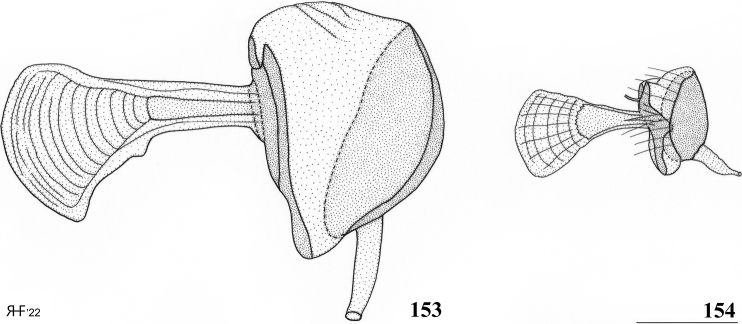
Ejaculatory apodeme + sac **153***Centrioncusdecoronotus*, paratype, Chania Falls **154***Teloglabrussanorum*, paratype, Drakensberg. Both drawings were processed based on original pencil drawings from Feijen (1983: figs 115, 126) and are drawn to the same scale. Scale bar: 0.2 mm.

Lonsdale (2013), in a review of Tanypezidae and Strongylophthalmyiidae, includes for the phylogenetic analysis in the character matrix as out-group representatives for the Diopsidae*Sphyracephalasubbifasciata* Fitch and *Centrioncusprodiopsis*. However, the origin of the single *Centrioncus* studied was not given, though for the genitalia character states, Feijen (1983) was used. The two specimens from Kenya were, after 106 years, the first *C.prodiopsis* collected since the type series. Literature references to additional *C.prodiopsis* records related to other *Centrioncus* species or to *Teloglabrus* species (Feijen 1983).

## ﻿Discussion

### ﻿Taxonomic position of the stalkless Diopsidae

Whether it will be necessary to keep Centrioncidae as a separate family remains to be seen. There are obviously striking differences compared to the stalk-eyed Diopsidae: no eye stalks, pubescent arista, ventrally extended funiculus, lanceolate basiliform prosternum, absence of pleurotergal spines, vein CuA+CuP reaching wing margin, presence of tubercles on hind femora, very large inverted male sternite 8 on both sides fused to sternite 7 which forms a complete ventral band of sclerotisation, and very different male genitalia (trilobed surstylus, presence of subepandrial clasper, small phallapodeme with posterior two-thirds fused to hypandrium and solid phallus with a complex distal section versus an open, delicate structure of sclerites and stylets coming together on a basal ring). On the other hand, Lonsdale (2020) lists as synapomorphies of the Diopsidae (including Centrioncinae) a scutellum with long apical spines, a bulbous katatergite, tarsi with dark “sawlines” lengthwise along the tarsomeres, absence of bm-m, a porrect antenna, raptorial fore legs, and only one outer vertical and one fronto-orbital seta. As far as DNA analyses is concerned, it appears that Centrioncinae and the stalk-eyed Diopsidae must have separated between 43 and 63 million years ago (F. A. A. Feijen, pers. comm. (2022); in Feijen et al. (2018) an introduction was given to her molecular analyses, but a full treatment is pending).

At present, we distinguish three subfamilies in the Diopsidae: Centrioncinae, Sphyracephalinae and Diopsinae (i.e., Diopsinae s.s.). This system was already proposed by Shillito (1971) and used by Feijen (1989) and Feijen and Feijen (2021). Hennig (1965) subdivided the Diopsidae into two subfamilies, Centrioncinae and Diopsinae (i.e., Diopsinae s.l.), while subdividing the latter subfamily into Sphyracephalini and Diopsini. Hennig’s system was followed by Steyskal (1972) and Meier and Baker (2002). Hennig (1965) regarded his subdivisions as relative in nature and thought it possible that Centrioncinae and Diopsinae s.l. could be regarded as families or tribes. For an absolute allocation of category in the Acalyptratae, Hennig thought it best to consider subfamilies the narrowest monophyletic groups originating from before the appearance of Baltic Amber (Eocene). From the presence of *Prosphyracephalasuccini* (Loew) in Baltic Amber, Hennig concluded that the sister-group relationship between Centrioncinae and Diopsinae s.l. originated from before this period. Based on what was then (1965) known about fossil diopsids, Hennig considered it as quite likely (“durchaus wahrscheinlich”) that in the Baltic Amber period only two diopsids were present: *P.succini* and a closely related (“sehr ähnlich”) species from which the Diopsini originated. Based on several plesiomorphic characters in the Diopsini genus *Diopsina* Curran which are not present in *Prosphyracephala* Hennig, Feijen (1981) considered that both Sphyracephalini and Diopsini originated from before the Eocene and consequently should be given subfamily rank. Hennig’s Centrioncinae and Diopsinae s.l. should then be elevated to family level.

Hennig’s (1965) assumption of only two diopsids present in the Baltic Amber period has now become dated. Lewis (1971) described the compression fossil *Prosphyracephalarubiensis* from the USA, while Kotrba (2009) described *Prosphyracephalakerneggeri* from Baltic Amber. A new development is that we are now in the possession of a Baltic Amber diopsid with large eye stalks for which it appears necessary to erect a new genus as a possible ancestor of Diopsinae s.s. This, in its turn would support the view to elevate Hennig’s Centrioncinae and Diopsinae s.l. to family level. Furthermore, photographic evidence is available for yet another undescribed *Prosphyracephala* species from Baltic Amber.

### ﻿Distinctive characters for *Centrioncus* and *Teloglabrus*

In Table 1, the differential character states for the genera *Centrioncus* and *Teloglabrus* are listed. The most important differences are indicated with an asterisk. Major apomorphic characters states for *Teloglabrus* are formed by the reduction of the anterior sclerite of female sternite 7 to two narrow elongate plates anteriorly connected on the meson, by the reduction of the posterior sclerite of female sternite 7 to two sclerites that are small to absent, and by the absence of microtrichia on the outer side of the outer and median arms of the surstylus. Major apomorphic characters states for *Centrioncus* are formed by the amazingly large (9.3–16.5% of body length) ejaculatory apodeme + sac and by the proximal section of ejaculatory duct turned perpendicular to the apodeme. The two genera are clearly allopatric in distribution.

McAlpine (1997) recommended treating *Teloglabrus* as junior synonym of *Centrioncus*, a view followed by Marshall et al. (2009) and Lonsdale (2020). Other authors (e.g., Baker 1999; Baker et al. 2001; Meier and Baker 2002; Carr 2008; Marshall 2012; Jackson 2019; Grace and Carr 2020) used *Teloglabrus* without discussing its status. De Meyer (2004) discussed both genera and noted the absence of synapomorphic characters for *Centrioncus*. He stated that his *C.bytebieri* “takes a somewhat intermediate position between the two genera”. Synapomorphies are now provided for *Centrioncus*, while *C.bytebieri* is confirmed as *Centrioncus*. Feijen and Feijen (2021), in a treatment of Afrotropical Diopsidae, provide a key to the genera and a synopsis of each genus.

Consensus on the status of *Teloglabrus* does not yet exist, but we think that the present paper supplies additional data to support the recognition of *Teloglabrus* as a valid entity. Lonsdale and Eiseman (2021) considered as main criteria for consideration in the erection of genera 1) monophyly and 2) utility. They considered that at the very minimum a genus should “represent a single monophyletic lineage that does not render other such groups non-monophyletic by its presence.” Beyond the issue of monophyly, the usefulness of a higher taxon was stated to be “linked to its practical utility in diagnostics, ...”. Such taxa should be ideally “diagnosed by multiple complex characters that are easily observed and present in all species”. We fully agree that pragmatism should be a fundamental element in classification. For the Centrioncinae, external characters are present but subtle. However, for the two genera differences between male and female genitalia are large. The generic differences in female sternite 7 can easily be observed and do not require dissection. The giant ejaculatory apodeme + sac of *Centrioncus* certainly requires dissection, but is unmistakable.

In Diopsidae, external morphological differences between species or genera are often subtle. We can refer to the differences between *Teleopsis* Rondani and *Megalabops* Frey (Feijen 2011; Feijen and Feijen 2019). Besides the molecular evidence, these genera are supported as distinct by large differences in the male and female genitalia. In addition, there are substantial differences in allometric data, *Teleopsis* being sexually dimorphic with regard to eye span, while *Megalabops* is monomorphic in this regard. However, other external morphological differences certainly exist but are usually not prominent. In the rather inaccessible genus *Diasemopsis* Rondani, external differences are often subtle but genital differences and allometric differences are pronounced.

As far as a pragmatic system should also allow for “predictiveness” (Lonsdale and Eiseman 2021), it can be stated that although the allopatric distribution of *Centrioncus* and *Teloglabrus* does not form an argument as such for the status of these genera, it can safely be predicted that the six undescribed Southern African species that we know off, fall within the concept of *Teloglabrus*.

### ﻿Intrageneric relationships in *Centrioncus*

Although the present key to species largely reflects the assumed relationships between *Centrioncus* species, in this discussion we will elaborate upon additional characters that were excluded from the key so as not to make it unwieldy. Based on the sets of differential characters presented in the species diagnoses, three groups of species can be distinguished. The first group can be referred to as the *C.aberrans* species-group. It includes *C.aberrans* and *C.crassifemur* sp. nov., and most probably *C.angusticercus*. Distantly related to this group are most likely *C.bururiensis* sp. nov. and *C.decellei*. It is unfortunate that in this group three species are only known from the female holotypes, and two holotypes were not available for the present study. The second group is named the *C.prodiopsis* species-group and comprises *C.prodiopsis* and *C.copelandi* sp. nov. The third group is the *Centrioncusdecoronotus* group and includes *C.decoronotus*, *C.jacobae*, and *C.bytebieri*.

The species of the *Centrioncusaberrans* group (including *C.bururiensis* sp. nov. and *C.decellei*) all have a blackish brown apex on the fore femur, while the five species in the *C.prodiopsis* and *C.decoronotus* groups have a brown stripe on the inner side of the fore femur. The three core species of the *C.aberrans* group have a high w/l ratio of the anterior sclerite of female sternite 7 and the posterior sclerite of female sternite 7 is trapezoidal to rectangular in shape. The seven other species have a lower w/l ratio of the anterior sclerite of female sternite 7 and the posterior sclerite of female sternite 7 is distinctly U-shaped. The two species of the *C.aberrans* group for which the male genitalia are known, *C.aberrans* and *C.bururiensis* sp. nov., have a very characteristic shape for the surstylus (Figs 21–24, 51), with a triangular outer arm with a base much broader than the apex, and a very broad median arm. The question is whether the three species *C.angusticercus*, *C.decellei* and *C.crassifemur* sp. nov. also have similar male genitalia. In contrast to the males of the *C.aberrans* group, the males of the *C.prodiopsis* group and the *C.decoronotus* group have all a deeply constricted base of the outer arm (Figs 69, 88, 122, 138, 150) and a slender median arm. Another difference between group 1 and groups 2 and 3 can be found in the male cercus. In the males of the *C.aberrans* group, the cercus gradually broadens distally, but there is no abrupt distal lateral extension (Figs 17, 54). That gives for this group a cercus with a length/greatest width ratio of 2.4–2.6. Unlike the males in the *C.aberrans* group, the males in groups 2 and 3 have a very distinct distal lateral extension of the cercus (Figs 71, 89, 123), with a length/greatest width ratio of 1.3–1.7.

Group 2, the *Centrioncusprodiopsis* species-group, is the most distinct one. The two species are characterised by the very large central wing spot, an absence of distal spots on the tergites, sternite 5 has a large mesal invagination anteriorly (Figs 80, 146), the female cercus has a l/w ratio of 4.0–4.4, there is an apical row of 16 or 17 tubercles on the outer arm of the surstylus, an absence of spinous setae on the median arm (Figs 88, 150), and an elongate subepandrial clasper (Figs 90, 151). In contrast, the species of group 3, the *C.decoronotus* species-group, have a small to large central wing spot, distal pale spots on the tergites, sternite 5 has a straight anterior edge (Figs 72, 118, 132), the female cercus has a l/w ratio of 2.3–3.6, there is an apical row of 4–9 tubercles on the outer arm of the surstylus, there are 3–6 spinous setae on the median arm (Figs 69, 122, 138), and a trapezoidal to triangular subepandrial clasper (Figs 69, 124, 138).

Within group 3, *Centrioncusdecoronotus* and *C.jacobae* appear to be more closely related based on the following character states: scutum with pattern of blackish brown and chestnut brown including brown humeral calli, fore femora with l/w ratio of 2.78–2.89, wing spot large and reaching crossvein dm-m in cell r4+5, sternite 4 rectangular, sternites 4 and 5 with heavily sclerotised areas, posterior sclerite of female sternite 7 without posterolateral extensions, subanal plate pentagonal, outer arm of surstylus rounded and subepandrial clasper strongly constricted basally. The same characters show the following states in *C.bytebieri*: scutum blackish brown including humeral calli, fore femora with l/w ratio of 3.30, wing spot small and not reaching crossvein dm-m in cell r4+5, sternite 4 trapezoidal, sternites 4 and 5 without heavily sclerotised areas, posterior sclerite of female sternite 7 with large posterolateral extensions, subanal plate triangular, outer arm of surstylus gradually tapering towards base and subepandrial clasper basally slightly constricted. These differences between *C.bytebieri* and the other two species appear convincing. However, there are also some similarities between *C.bytebieri* and *C.decoronotus* that are not found in *C.jacobae*, like larger scutellar spines (scutellar spine/length of body ratio 0.90–0.95 vs. 0.78), a shorter, narrower stripe on the fore femur, small posterolateral spots on tergite 2 (vs. large spots), and a long and slender common base of the outer and median arms of surstylus (vs. a short, broad base).

### ﻿Measurements and ratios in *Centrioncus*

It is obvious that for differential characters in the Centrioncinae, male and female genitalia and other abdominal structures must be used. Specific differences in other morphological differences are small, although there are exceptions, such as the size and strikingly aberrant colour in *Teloglabrusstuckenbergi* Feijen. However, even when very characteristic scutal patterns exist, as in *Centrioncusdecoronotus* and *C.jacobae*, these are often obscured by greasiness or other causes. Therefore, measurements and various ratios can give additional useful differential characters. As shown in Tables 6, 7, quite a number of these characters show distinct differences. Compared with other Diopsidae taxa, there is a remarkable lack of difference between the sexes in measurements, body ratios and number of spines and tubercles (Tables 2–5). Several of the more important differential characters regarding measurements and ratios are highlighted below.

For *Centrioncuscrassifemur* sp. nov., its most striking external character is formed by its incrassate fore femur with a l/w ratio of 2.36. This is based on a single specimen, but is clearly distinct from the ratio in *C.aberrans* of 2.75 ± SE 0.01 (range 2.68–2.84, *n* = 22), *C.bytebieri* of 3.30 ± 0.02 (range 3.17–3.43, *n* = 16), *C.decoronotus* of 2.89 ± 0.01 (range 2.75–3.05, *n* = 43) and *C.jacobae* of 2.78 ± 0.02 (range 2.64–2.92, *n* = 18). This set of ratios also clearly shows that the fore femur in *C.bytebieri* is less incrassate than the other three species with large data sets. Comparing the four sets of ranges for the ratios in the four species shows that there is not even any overlap in the ratios for *C.bytebieri* and those of the other three species. In other taxa of Diopsidae, the l/w ratios of the fore femur have also proven to be very useful (see e.g., Feijen et al. 2018).

Examination of Tables 6, 7 clearly shows *Centrioncusbytebieri* as the largest species with a body length of 5.55 mm ± SE 0.09, against 5.16 mm ± 0.04 for *C.aberrans*, 5.15 mm ± 0.04 for *C.decoronotus* and 4.97 mm ± 0.06 for *C.jacobae*. The larger body length for *C.bytebieri* corresponds to other larger absolute size data such as for wing, scutellum, scutellar spine and apical seta (Tables 6, 7). However, that does not run true for all quantitative characters. The width of head is, for instance, largest in *C.aberrans*, while the number of tubercles on the fore femur and the number of spinous setae on the fore femur are the lowest in *C.bytebieri*. For relative data, useful differences can also be found. The scutellar spine, as compared to both the length of the scutellum and the body length, is, for instance, distinctly smaller in *C.aberrans* and *C.jacobae* than in *C.bytebieri* and *C.decoronotus* (Tables 6, 7).

For postabdominal characters, quantitative particulars can be very useful. The w/l ratio for the anterior sclerite of female sternite 7 forms an important differential character at species level, but also at species-group level. This ratio is high in the core species of the *Centrioncusaberrans* group with values between 4.4 and 8.6, while in other species the ratio is 2.5–3.9 (Table 8). In the distant relatives in the *C.aberrans* group, *C.decellei* and *C.bururiensis* sp. nov., the score is only 2.6–2.7. Especially *C.crassifemur* sp. nov. has a very wide and short anterior sclerite with a ratio of 8.6. In the *C.decoronotus* species-group, *C.decoronotus* has a high score of 3.9, while the other two species, *C.bytebieri* and *C.jacobae* have both a score of 2.6. The l/w ratio of the female cercus is another useful quantitative character (Table 8). These ratios partly correspond to the values for the ratio width/length for the anterior sclerite of female sternite 7. *Centrioncusangusticercus* and *C.aberrans* have high scores of 5.1–5.4, while the species of the *C.decoronotus* group have low values of 2.3–3.6. The shape of the male cercus has, in the first place, to be described qualitatively with the large and highly unusual lateral extension in the distal third in species of the *C.prodiopsis* group and the *C.decoronotus* group. Just as well, the quantitative length/greatest width ratio is also useful with values for the males in the *C.aberrans* group of 2.4–2.6 and values in the other two species-groups of 1.4–1.7 (Table 8).

An important quantitative character is formed by the relative size of the ejaculatory apodeme + sac as compared to the body length (Table 9). While making genital preparations, the giant ejaculatory apodeme + sac in *Centrioncus* can be observed with the naked eye. In *Centrioncusdecoronotus*, the size comes close to 1 millimetre (Table 9). This quantitative character forms a main difference between the genera *Centrioncus* and *Teloglabrus* with ejaculatory apodeme + sac/length of body ratios for *Centrioncus* of 9.3–16.5 and for *Teloglabrus* of 5.6–8.4 (usually 5.6–7.3). The difference between *C.decoronotus* and *Teloglabrussanorum* Feijen for the size of ejaculatory apodeme + sac is striking (Figs 153, 154). Feijen (1983: figs 94, 95, 115–133) illustrated the ejaculatory apodeme + sac of many *Centrioncus* and *Teloglabrus*. Now that *C.aberrans* has also been shown to have a large ejaculatory apodeme + sac, the large size of ejaculatory apodeme + sac can be considered an autapomorphic character of *Centrioncus*. Some intraspecific variation occurs for this quantitative character (see Table 9), but this can be due to the ejaculatory sac being fully extended or not.

### ﻿Colour patterns in *Centrioncus*

Smithers (1958) and Feijen (1983) remarked on the special tendency of Centrioncinae to discolour after death. Colour contrasts can become accentuated by preservation in alcohol. In pinned specimens the flies can become darker and “greasy” if some time lapses between collecting and pinning, especially when kept in a closed container. This tendency to become greasy has never been observed in stalk-eyed Diopsidae and might form an indication for a major physiological difference. In *Centrioncus*, *C.decoronotus* and *C.jacobae* can have striking pattern of brown and blackish brown on the thorax (Figs 112, 127). However, this pattern is not always visible in collection specimens, while it can even be much less pronounced in live specimens.

The abdomen is usually blackish brown, but can have pale posterolateral spots and/or whitish microtrichose posterior edges. Large and striking posterolateral spots occur on tergite 2 only in *Centrioncusjacobae* (Fig. 135). The colour pattern of the fore femur is a useful and stable differential character. The basic colour is pale yellowish brown, but the apex can be dark brown in varying degrees or have a dark brown stripe on the inner side.

Wing patterns form a differential character in the Centrioncinae, but it should be stressed that the patterns are quite vague, certainly if compared with the often very dark spots in many stalk-eyed diopsids. As such, it is usually necessary to prepare wing slides to properly see the spots. In Centrioncinae, the central wing spot forms a differential character at the genus level. It is present in all *Centrioncus* and in only two *Teloglabrus*. However, De Meyer (2004) stated that no central wing spot occurs in *C.bytebieri*. After cursory examination of paratypes at MRAC, we initially agreed to this observation, but from examination of additional specimens it became clear that *C.bytebieri* has a central wing spot (Fig. 3).

### ﻿Antennae in Diopsidae

For the Diopsidae, Feijen (1983: fig. 4) illustrated the antenna of *Teloglabrussanorum*, *Sphyracephalabeccarii* (Rondani) and a *Diopsis* of the *cruciata* species-group (wrongly identified as *D.phlogodes*). Feijen (1984: fig. 1) illustrated the antenna of a *Diopsina*, while Feijen (1989: figs 18, 46) provided drawings of the antennae of two Nearctic *Sphyracephala* Say. Feijen et al. (2018) illustrated the antenna and basal arista for six Madagascar diopsids. McAlpine (2011: figs 122–125), in an extensive study on antennal morphology in Diptera, described and illustrated the antennae of *Centrioncusdecoronotus* and *S.beccarii* and briefly described the antenna of a species of *Cyrtodiopsis*. McAlpine described the antenna of *Centrioncus* as follows: “the conus [fig. 122] is deep, somewhat bilaterally compressed, and asymmetrical, with laterally facing preapical foramen; segment 3 [fig. 124] has its basal foramen inside the basal hollow on its lateral wall; the arista is inserted slightly laterally to the dorsal margin of segment 3.” On the antenna of Centrioncinae he remarked that it is “more like that of various basal schizophoran types found in the Sciomyzoidea and Heteromyzoidea than is that of the Diopsinae, though it may partly retain the plesiomorphic structure from which that of the Diopsinae was derived.” The antenna of the Centrioncinae is indeed quite different from those of the stalk-eyed Diopsidae with a pubescent arista, a distally triangular pedicellus projecting into the dorsal section of the funiculus, and the ventrally strongly extended funiculus (Fig. 38).

### ﻿Facial sulcus in Diopsidae

The median suture-like groove of the facial region in many stalk-eyed Diopsidae has been referred to as the facial sulcus (Shillito 1971; Feijen 1989). According to McAlpine (1997), the facial sulcus does not divide the face on the meson, but “the facial sulcus is actually the face, which has become greatly narrowed in more advanced diopsids”. The facial sulcus is absent in Centrioncinae (Figs 11, 95). In the fossil genus *Prosphyracephala*, no facial sulcus is found, but a large, triangular plate is present centrally in the face (e.g., Kotrba 2009: fig. 7). This plate can even be somewhat elevated. The facial sulcus is absent or reduced in several *Sphyracephala* and *Cladodiopsis* (e.g., Feijen and Feijen 2019: figs 4, 5). McAlpine (1997) assumed the “supposed absence of the sulcus in species of *Sphyracephala*” to be due to “smaller difference in degree of sclerotisation between parafacial and face and less narrowing of the face”. McAlpine’s explanation of the facial sulcus is very interesting but would require more study of its distribution and internal structure among the stalk-eyed Diopsidae, especially the Sphyracephalinae.

### ﻿Scutal setae and supra-alar ridge (carina) in Diopsidae

Feijen (1983) listed three scutal setae for Centrioncinae: a presutural seta, a supra-alar seta (SA) and an infra-alar seta (IA). However, the correct name for the presutural seta is posterior notopleural seta (PNS) according to McAlpine (1997). The IA is the largest followed by the PNS. The SA was described as rather small and standing on a small ridge. Feijen (1983) also remarked: “The SA also occurs in various *Diopsina* ...., although in *Centrioncus* this seta stands on a small ridge and as such perhaps might not be homologous to the SA in *Diopsina*.” Feijen (1989) listed the various scutal setae in the Diopsidae. An SA is only present in three stalk-eyed diopsids: *Diopsinanitida* (Adams), *Diopsinadraconigena* Feijen and *Diopsinafluegeli* Feijen & Feijen (Feijen and Feijen 2013). In addition, the stalk-eyed genera *Teleopsis* Rondani and *Megalabops* Frey have a pair of scutal spines which are referred to as supra-alar spines. McAlpine (1997) considered the supra-alar ridge (named carina) as a structure which is peculiar to the Syringogastridae and Diopsidae. This carina was illustrated for *Centrioncusdecoronotus* (McAlpine 1997: fig. 35). McAlpine considered the carina “probably at its most primitive condition in *Centrioncus* (including *Teloglabrus* ...), where it consists of a well-defined ridge on the surface of the mesoscutum passing posterodorsally from the postnotopleural ridge ... to the base of the supra-alar bristle”. McAlpine further stated that the supra-alar seta is vestigial or absent in Syringogastridae and most Diopsinae, but the carina remains. According to McAlpine the carina, as such, is not present in the genus *Teleopsis* but “the summit of the supra-alar carina is produced as a large spinous process (the supra-alar spine) and the lower part of the carina is more or less obsolete. Given the differing views on the placement of Syringogastridae and Diopsidae (Meier and Baker 2002 and Marshall et al. 2009 vs. Wiegmann et al. 2011), the question is whether the carinas in Syringogastridae and Diopsidae s.l. can be considered synapomorphies.

### ﻿Pleurotergite in Diopsidae

Shillito (1950) in his paper transferring *Centrioncus* to the Diopsidae remarked “The pleurotergal spine characterizing the Diopsidae is replaced by a dome-like swelling” and in his revised family definition: “The thorax shows modification of the pleurotergite into a dome-shaped swelling or a well-developed spine”. The suggestion that the swelling is likely replaced by a spine is not correct. In the stalk-eyed Diopsidae, a bulbous pleurotergite occurs just like in the Centrioncinae. In the stalk-eyed Diopsidae there is, in addition, a pleurotergal spine (sometimes called metapleural by mistake) centrally or dorsally on this bulbous pleurotergite. Size and direction of this spine are major differential characters in the stalk-eyed Diopsidae. Spine and swelling have usually been labelled as pleurotergal, but Lonsdale (2020) places these structures on the katatergite, the ventral section of the pleurotergite.

### ﻿Sternopleura (= katepisternum) in Diopsoinea

McAlpine (1997) noted the pitting along the median ventral suture of the sternopleura in some taxa of Diopsoinea (Syringogastridae + Diopsidae) as an interesting character. For *Syringogaster* sp., McAlpine described a single median series of relatively few, spaced, deep pits. In *Centrioncus* spp., he found a similar condition, but the pits were more numerous. In most stalk-eyed Diopsidae the median sternopleural suture was externally visible as a groove or ridge, but without associated pits associated. Only in *Sphyracephala* sp., McAlpine found a series of pits along each side of a slight ridge.

### ﻿Preabdominal sternites in *Centrioncus*

In most *Centrioncus*, sternite 1 is a short, rectangular sclerite with somewhat concave anterior and posterior sides. Sternite 2 is the longest sternite and is usually rectangular. In many stalk-eyed Diopsidae a tiny, strongly sclerotised sternite occurs in between sternites 1 and 2. This sclerite is referred to as intersternite 1–2. This sclerite has been regularly noted and illustrated, and was specifically discussed by Feijen (1989) and Feijen et al. (2018). In the latter paper, it was confirmed that this sclerite originates from sternite 2. Its shape can be a useful differential character even at genus-level. In most stalk-eyed flies it is not connected to sternite 2, but in *Gracilopsina* Feijen, Feijen and Feijen it is laterally connected to sternite 2. Feijen et al. (2018) considered the character state in *Gracilopsina* as the plesiomorphic state. Intersternite 1–2 is usually a small line-like sclerite with no connection to sternite 2. It can also be absent. In *Sphyracephalanigrimana* Loew, Feijen et al. (2018), found a situation comparable to the one in *Gracilopsina*, while “in two other *Sphyracephala* the mesal anterior edge of sternum 2 is more sclerotised”. Feijen et al. (2018) stated that “In the Centrioncinae intersternite 1–2 is absent”. However, this remark is now shown to be mistaken. In *Centrioncusbururiensis* sp. nov., *C.copelandi* sp. nov., *C.decoronotus*, *C.jacobae* and *C.prodiopsis*, spindle-shaped well-sclerotised central sections anteriorly of main sternite 2 have short lateral connections to sternite 2 (Figs 41, 80, 118, 132, 146). These sections can be referred to as intersternite 1–2. *Centrioncusaberrans* (Fig. 13) also has an intersternite 1–2, but lateral connections to main sternite are long. In *C.bytebieri* and *C.crassifemur* sp. nov., the well-sclerotised slender mesal anterior section of sternite 2 forms an integral part of sternite 2 (Figs 72, 101), a situation like the one in the two *Sphyracephala*. The state in the two *Sphyracephala* and the two *Centrioncus* can be considered the most plesiomorphic state, while the intersternites with lateral connections form the less plesiomorphic state. The presence of an intersternite, although considered apomorphic, was already verified in a Baltic amber diopsid with long eyestalks.

Sternite 3 is rectangular in most species and square in two species. Sternite 4 shows more variation and can be square, square to rounded or trapezoidal. Five species (*Centrioncusbururiensis* sp. nov., *C.copelandi* sp. nov., *C.decoronotus*, *C.jacobae*, and *C.prodiopsis*) have anteriorly one or two pairs of small, heavily sclerotised areas in sternite 4 (Figs 41, 80, 118, 132, 146). Sternite 5 shows also much interspecific variation. It can be rectangular, square or trapezoidal. In *C.copelandi* sp. nov. and *C.prodiopsis*, sternite 5 is anteriorly strongly invaginated on the meson, while five species (*C.bururiensis* sp. nov., *C.copelandi* sp. nov., *C.decoronotus*, *C.jacobae*, and *C.prodiopsis*) have anteriorly one pair of small, heavily sclerotised areas (Figs 41, 80, 118, 132, 146). Sternite 6 is in most species short, trapezoidal and broad. *Centrioncusdecoronotus* is the only species in which sternite 6 is not so broad, while it also has an anterior pair of small, heavily sclerotised areas (Fig. 118).

### ﻿Tarsal and tibial sawlines in Diopsidae

McAlpine (1997) stressed the phylogenetic importance of tarsal sawlines in the Diopsoidea. According to McAlpine, a tarsal saw line consists of a “well defined linear longitudinal series of short, compressed cuneate setulae situated on either the anterior or posterior side of a tarsal segment”. For *Centrioncusdecoronotus*, McAlpine (1997: fig. 42) presented a highly magnified (x 1740) part of the posterior sawline of the mid basitarsus. For the combination of Syringogastridae and Diopsidae, the presence of a tarsal saw line, at least on the posterior side of the mid basitarsus, was considered a synapomorphy, a view followed by Lonsdale (2020). Within the Diopsidae, McAlpine noted differences with regard to the presence of tarsal sawlines. In *Centrioncus*, sawlines were only present on the posterior surfaces of mid tarsal segments 1–4, extending along most of the length of the mid tibia on the posterior surface. For the stalk-eyed Diopsidae, various *Sphyracephala* were found to be different in the absence of sawlines on the hind tarsus, whereas other genera had at least the anterior side of hind tarsal segment 1 with a sawline. McAlpine considered the reduction of sawlines in *Sphyracephala* an apparently derived state within the Syringogastridae + Diopsidae. A sawline on the fore tarsus was according to McAlpine (1997) only present in, what he called, the advanced diopsine genera, like *Diasemopsis* Rondani, *Cyrtodiopsis* Frey, *Teleopsis*, and *Diopsis*.

### ﻿Setal formula for legs

Feijen (1983) used a “setal formula” to describe the numbers of spinous seta and tubercles on the legs of Centrioncinae. In, for instance, *Centrioncusaberrans* a setal formula of 4.1, 4.9, 18.2, 17.2, 6.5 is given. The numbers refer to, respectively, mean of F1 bristles on outer row, mean of F1 bristles on inner row, mean of F1 tubercles on outer row, mean of F1 tubercles on inner row and mean of tubercles on F3. This formula represents a useful character for species. However, given the large ranges in numbers of tubercles and spinous setae (see Table 2), it must be stressed that this setal formula is only useful if based on large series of specimens. In stalk-eyed Diopsidae, the number of spinous seta and tubercles on the fore femur also forms an important character at species and genus level.

### ﻿Subcostal cell

In *Centrioncusaberrans*, the subcostal cell is not visible in most specimens as in the flies of the type series and in all other *Centrioncus*. However, the subcostal cell is visible in one specimen from Rwanda and one specimen of Mt. Elgon. The visibility of the subcostal cell can therefore no longer be regarded as a reliable character as stated in Feijen (1983).

### ﻿Molecular analyses for Diopsidae

Molecular systematics for Diopsidae started with the Ph.D. thesis of Baker (1999), which presented a phylogenetic analysis of 33 diopsid species using a large molecular data set. This study included representatives of *Cyrtodiopsis*, *Diasemopsis*, *Diopsis*, *Eurydiopsis* Frey, *Sphyracephala*, and *Teleopsis*, while the Centrioncinae were represented by *Teloglabrusentabenensis* Feijen and *Teloglabrusmilleri* Feijen. After Baker (1999) and Baker et al. (2001), molecular studies on Diopsidae were usually based on the same data set, with additional data on some other Diopsidae species. For Centrioncinae, only Jackson (2019) added extra information for a *Teloglabrus* not identified to species level. To date, molecular data on *Centrioncus* have not been published.

### ﻿Sex ratio in *Centrioncus*

In the *Centrioncus* species of which larger series are available, an even sex ratio was found: 36 ♀ and 32 ♂ in *C.bytebieri*, 56 ♀ and 53 ♂ in *C.decoronotus* and 32 ♀ and 39 ♂ in *C.jacobae*. For all known *Centrioncus* flies taken together we found 146 ♀ and 147 ♂. In the stalk-eyed Diopsidae aberrant sex ratios, usually favouring the females, are often encountered (Burkhardt and de la Motte 1985; Feijen 1989; Wilkinson et al. 1998; Paczolt et al. 2017). Burkhardt and de la Motte found that “the sex ratio of freshly emerged dimorphic flies deviated significantly from the 1:1 ratio in favour of the females” while in “cultures of the homomorphic species no significant deviations were found”. Wilkinson et al. stated that “By comparing sex-ratio distributions in stalk-eyed fly (*Cyrtodiopsis*) progeny we found that female-biased sex ratios occur in species exhibiting eye-stalk sexual dimorphism and female preferences for long eye span.” It is certainly not the case that all dimorphic (with regards to eye span) Diopsidae have aberrant sex ratios as indicated by Feijen (1989). In *Centrioncus*, homomorphic with regard to head morphology, no aberrant sex ratios appear to occur.

### ﻿Fungal parasites (Laboulbeniales) of Centrioncinae

Feijen (1983) discussed the presence of fungal parasites (Ascomycota, Laboulbeniales) in *Centrioncus* and *Teloglabrus*. From these two genera, only the genus *Rhizomyces* Thaxter was known, but the species could not be determined. Rossi and Feijen (2018) described *Rhizomycesforcipatus* from *C.decoronotus*, *C.jacobae*, *C.decellei*, *Teloglabrus* sp. from South Africa, and *Teloglabrusaustralis* Feijen. Rossi and Feijen also noted infestation by this fungal parasite for *Centrioncusprodiopsis*, *Teloglabruscurvipes* Feijen, *Teloglabrusduplospinosus* Feijen, *Teloglabruspelecyformis* Feijen, *Teloglabrussanorum*, *Teloglabrustsitsikamensis* Feijen, *Teloglabrustrituberculatus* Feijen, and *Teloglabrusvumbensis* Feijen. Information was supplied on the prevalence of *R.forcipatus* on its various hosts. Rossi and Feijen indicated that *R.forcipatus* belongs to the group of *Rhizomyces* species lacking haustoria. The species of this group are only known to occur in *Centrioncus*, *Teloglabrus* and the *Diasemopsis* genus-group.

## Supplementary Material

XML Treatment for Diopsidae

XML Treatment for Centrioncinae

XML Treatment for Centrioncus

XML Treatment for Centrioncus aberrans

XML Treatment for Centrioncus angusticercus

XML Treatment for Centrioncus bururiensis

XML Treatment for Centrioncus bytebieri

XML Treatment for Centrioncus copelandi

XML Treatment for Centrioncus crassifemur

XML Treatment for Centrioncus decellei

XML Treatment for Centrioncus decoronotus

XML Treatment for Centrioncus jacobae

XML Treatment for Centrioncus prodiopsis
